# Which exercise modality is most effective for improving cardiac function in patients with myocardial infarction? A network meta-analysis

**DOI:** 10.3389/fcvm.2025.1623727

**Published:** 2025-10-30

**Authors:** Bo Yu, Linlin Zhao, Liangyi Huang, Maolin Zhang

**Affiliations:** ^1^ School of Wushu, Shandong Sport University, Jinan, China; ^2^ Traditional Sport Institute, Harbin Sport University, Harbin, China; ^3^ Zaoyang Children’s Sports School, Zaoyang, China

**Keywords:** myocardial infarction, exercise training, cardiac function, cardiac rehabilitation, network meta-analysis

## Abstract

**Objective:**

This study aimed to compare the efficacy of different exercise modalities on cardiac function in patients with myocardial infarction (MI), providing evidence-based recommendations for optimal cardiac rehabilitation programming.

**Methods:**

We conducted a systematic search of seven Chinese and English databases, including CNKI and Web of Science, to identify eligible studies. A network meta-analysis based on the frequency framework was performed using STATA 14.0.

**Results:**

A total of 69 studies involving 5,044 participants were included. Compared to the control group, all exercise interventions significantly improved 6-minute walk test (6MWT) scores in MI patients, with mean differences (MDs) and 95% confidence intervals (CIs) ranging from 57.61 (34.87, 80.36) for aerobic exercise (AE) to 144.38 (110.78, 177.98) for resistance exercise (RE). All modalities enhanced left ventricular ejection fraction (LVEF), with MDs (95% CI) from 4.75 (3.42, 6.09) for AE to 8.75 (5.72, 11.77) for RE. Except for AE, all interventions reduced left ventricular end-diastolic diameter (LVEDD), with MDs (95% CI) from −4.01 (−6.42, −1.59) for multi-component exercise training (MCET) to −6.40 (−9.24, −3.56) for RE. All exercises improved left ventricular end-systolic diameter (LVESD), with MDs (95% CI) from −1.89 (−3.27, −0.51) for AE to −7.33 (−9.62, −5.03) for RE. RE consistently showed a high probability of relatively high efficacy rankings across outcomes (SUCRA: 93.2–99.8).

**Conclusion:**

RE appeared to have a high probability of being a highly effective single modality for improving post-MI cardiac function and remodeling. MCET and mind-body training also offer notable advantages, particularly in reducing ventricular size. Ultimately, rehabilitation programs should be tailored by considering the modality-specific benefits, patient's clinical profile, and functional capacity to optimize outcomes.

**Systematic Review Registration:**

https://inplasy.com/inplasy-2024-11-0016/, identifier INPLASY2024110016.

## 1 Introduction

Myocardial infarction (MI) is a myocardial necrosis event caused by unstable ischemic syndromes (1), and it remains the leading cause of mortality among cardiovascular diseases (2). This high mortality rate is primarily due to coronary artery stenosis and occlusion, which lead to acute or sustained myocardial ischemia and hypoxia, ultimately resulting in myocardial infarction (3, 4). As one of the major causes of death from coronary heart disease (CHD), MI accounts for over 4 million deaths in Europe and Northeast Asia and is responsible for more than a third of all annual deaths in developed countries (5). In China alone, approximately 2.5 million individuals currently live with MI, with projections estimating an additional 7.5 million cases in the next 15 years (6), and a concerning trend toward younger onset ages (7, 8). Studies show that adverse left ventricular remodeling and heart failure following MI significantly impair patients' quality of life (9). Standard treatments for MI typically include percutaneous coronary intervention (PCI) and coronary artery bypass grafting (CABG), which improve clinical outcomes, increase survival rates, and reduce mortality (10). However, long-term prognosis—such as effectively managing risk factors, enhancing quality of life, and reducing the recurrence of acute cardiac events—relies heavily on exercise-based cardiac rehabilitation (CR) (11). Therefore, improving the long-term health outcomes and prognosis for MI survivors represents a critical global public health challenge and a focal point in cardiovascular medicine.

Recent studies have demonstrated that regular physical activity is an effective behavioral intervention for improving heart health (12), serving as a key protective factor for MI patients in achieving favorable recovery, low incidence, and reduced mortality risk. Regular exercise exerts an anti-atherosclerotic effect on the vascular system, improves autonomic balance (which lowers the likelihood of dangerous arrhythmias), and promotes myocardial safeguarding from ischemia-reperfusion damage (13). Several studies have shown that exercise-based CR can delay the progression of coronary atherosclerosis, improve long-term mortality in cardiovascular patients, enhance aerobic capacity, and increase quality of life (14, 15). While the benefits of exercise-based CR for CVD patients are widely recognized, the optimal exercise modalities and intensities remain a subject of debate. Some research suggests that aerobic exercise (AE) is an effective form of rehabilitation for enhancing cardiovascular and cardiopulmonary health, with most clinical studies favoring low-intensity, long-duration exercise as the standard for cardiac rehabilitation (16). However, the growing attention given to high-intensity interval training (HIIT) has led to substantial evidence indicating that HIIT is particularly effective in the cardiac rehabilitation of cardiovascular patients, offering advantages over moderate-intensity continuous training (MICT) (17, 18). Furthermore, recent meta-analyses suggest that mind-body exercises (MBE), such as Tai Chi, Baduanjin, and Qigong, are effective in improving cardiac rehabilitation and enhancing cardiopulmonary health in MI patients (19–21). Additionally, recent research has confirmed that resistance exercise (RE) is safe for patients with stable heart failure and has beneficial effects in preventing muscle atrophy and increasing muscle strength and endurance (22).

To date, existing meta-analyses have primarily focused on the effects of single interventions, such as AE (11, 19, 23) or HIIT (24), without conducting a systematic review of how various exercise modalities influence cardiac function in MI patients. More importantly, it is still uncertain which exercise type is the most effective at enhancing cardiac function in these patients. Network meta-analysis (NMA), often referred to as multiple treatment comparison meta-analysis, facilitates a simultaneous comparison of three or more interventions, expanding the scope beyond traditional pairwise analysis. Even in the absence of direct comparisons between two interventions, NMA enables the estimation of the relative effectiveness of all interventions and ranks them accordingly, significantly enhancing the precision of the results (25). Therefore, this study aims to perform an NMA of relevant randomized controlled trials (RCTs) to provide a comprehensive assessment of the effects of mind-body exercise, AE, RE, HIIT, and combined exercise on cardiac function in MI patients, offering stronger evidence to guide the selection of effective cardiac rehabilitation strategies for this population.

## 2 Methods

This study adhered to the PRISMA (Preferred Reporting Items for Systematic Reviews and Meta-Analyses) Extension Statement (Supplementary Appendix 1) and has been registered with INPLASY (International Platform of Registered Systematic Review and Meta-analysis Protocols) (Registration Number: INPLASY2024110016).

### 2.1 Search strategy

We conducted a comprehensive search of eight major Chinese and English databases: China National Knowledge Infrastructure (CNKI), Wanfang Database, Chinese Science and Technology Periodical Database (CSTJ), China Biomedical Database, PubMed, Web of Science, Embase, and The Cochrane Library. The search period spanned from the inception of each database to January 10, 2025. A combination of subject headings and free-text terms was used, with primary search terms including “myocardial infarction”, “cardiovascular strokes”, “exercise”, and “cardiac function”. Detailed search strategies for each database are presented in Supplementary Appendix 2.

### 2.2 Inclusion criteria

Eligible studies were those that fulfilled these criteria: (1) participants: Adults over the age of 18 who have been diagnosed with myocardial infarction based on clinical examinations such as PCI, dynamic electrocardiogram monitoring, serum or enzymatic tests, x-rays, echocardiography, or coronary angiography (26). No restrictions were placed on participants' race, nationality, or region. (2) Interventions: The control group was administered conventional treatments, including regular medication, typical care, and verbal instruction. The experimental group received exercise interventions in addition to the control treatments, including AE, RE, MBE, HIIT, and multi-component exercise training (MCET). Studies comparing different exercise modalities were also considered. Specific definitions and examples of the various exercise forms are provided in Supplementary Appendix 3. (3) Outcome Measures: Cardiac function was assessed using the six-minute walk test (6WMT), left ventricular ejection fraction (LVEF), left ventricular end-diastolic diameter (LVEDD), and left ventricular end-systolic diameter (LVESD). (4) Study Design: RCTs.

Exclusion criteria included: non-randomized controlled trials; duplicate publications; animal studies; mechanistic pharmacology or drug synthesis research; reviews; studies without clear descriptions of exercise interventions; studies involving participants who were not MI patients; and studies with incomplete data.

### 2.3 Study selection and data collection

Two researchers conducted screenings of the literature independently to assess eligibility. Duplicate records were removed using reference management software. Afterward, titles and abstracts were reviewed for an initial selection, and the full texts of the remaining articles were downloaded to confirm eligibility for inclusion in the analysis. Discrepancies were resolved through discussion, or a third researcher acted as an arbitrator to determine whether a study should be included.

A pre-designed data extraction form was utilized to systematically collect and organize pertinent information from the studies included in this analysis. The collected data encompassed the author(s), year of publication, average age of participants, gender distribution, specific interventions implemented in both the experimental and control groups, as well as the means and standard deviations recorded prior to and following the interventions, in addition to the sample size. In instances where data were found to be incomplete, the authors of the respective studies were contacted to obtain the necessary information.

### 2.4 Risk of bias and quality of evidence assessment

Two researchers assessed the risk of bias in the included studies using the Revised Cochrane Risk-of-Bias Tool for Randomized Trials (ROB2) (27). The risk of bias was evaluated across five domains: bias arising from the randomization process; bias due to deviations from the intended interventions; bias from missing outcome data; bias in outcome measurement; and bias due to selective reporting. The overall risk of bias for each study was determined by synthesizing the results from these five domains. Each domain was classified as having high, low, or some risk of bias.

The quality of the evidence was assessed using the CINeMA online tool (28). CINeMA evaluated the risk of bias across six domains: within-study bias, between-study bias, indirectness, imprecision, heterogeneity, and inconsistency. Based on these assessments, the quality of evidence was classified as high, moderate, low, or very low (29). Detailed assessment methods for each domain are provided in Supplementary Appendix 7.

### 2.5 Statistical analysis

Given that all outcome measures were continuous variables employing identical measurement techniques and units, mean differences (MD) along with their associated 95% confidence intervals (CIs) were utilized to evaluate effect sizes. The NMA was conducted using a frequentist framework in Stata 14.0 (30). A random-effects model was applied to account for heterogeneity across studies due to various factors, providing more conservative confidence intervals (31). This model has been widely utilized in previous studies, and its effectiveness has been verified (32, 33). Network plots were used to visualize the comparisons between interventions, and both the design-by-treatment interaction model and side-splitting methods were employed to assess global and local inconsistency (34, 35). When the global inconsistency test showed no significant results, a consistency model was used for analysis. The Surface Under the Cumulative Ranking (SUCRA) was calculated to determine the relative effectiveness of different interventions, with higher SUCRA values indicating better treatment efficacy (36). It is important to note that the ranking probabilities estimated by SUCRA values should not be interpreted in isolation but rather as one component of a comprehensive assessment that gives greater weight to the magnitude of the MDs, the precision of these estimates (95% CIs), and the overall quality of evidence as assessed by CINeMA. Additionally, network meta-regression was conducted to explore the potential impact of participant age, exercise intervention duration, and baseline severity on the study outcomes. Sensitivity analysis was conducted by excluding studies with a high risk of bias to assess their impact on the results of the NMA. Publication bias was assessed by visual inspection of the comparison-adjusted funnel plot for each outcome network.

## 3 Results

### 3.1 Characteristics of included studies

**Figure 1:**
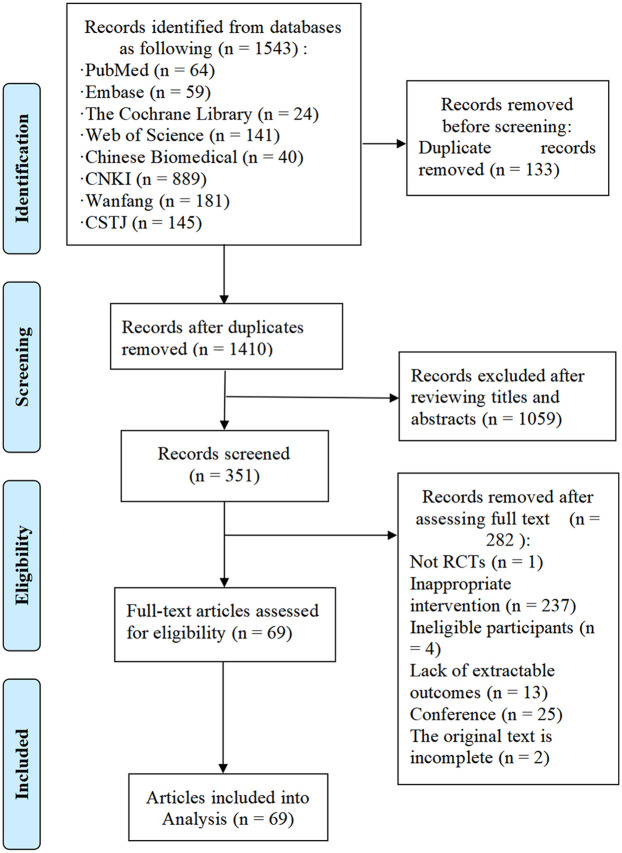
Flow diagram.

A total of 1,543 studies were initially retrieved from the databases, with 133 duplicates removed. After screening titles and abstracts, 351 studies were considered potentially eligible. Following full-text review, 69 studies were deemed to meet the inclusion criteria. Figure 1 illustrates the detailed literature selection process and Table 1 presents the brief characteristics of the included studies. The majority of the included studies were two-arm trials, comparing different interventions, with only two studies being three-arm trials (37, 38). Among the interventions, AE and mind-body exercise were the most commonly used. Additionally, most studies involving MCET focused on the combined effects of AE and RE. The mean intervention durations were approximately 15.4 weeks for RE, 16.5 weeks for AE, 13.5 weeks for mind-body exercise, 11.8 weeks for HIIT, and 15.2 weeks for MCET. Regarding the assessment of cardiac function outcomes, only two studies, Jonathan Myers et al. (39) and Schmid et al. (40), utilized cardiac magnetic resonance (cardiac MRI); all other studies used echocardiography for these measurements. Detailed characteristics of the included studies are provided in Supplementary Appendix 4.

**Table 1:** Brief characteristics of the included studies.

Author/Year	Country	Sample size	Gender (Male/Female)	Age (*X¯* ± *s*)	Intervention	Length (week)	Frequency (times/week)	Duration/Per session (min)	Outcomes
Ding et al. 2024	China	45	33/12	64.83 ± 4.63	RE	12	7	20	6MWT/LVEF/LVESD/LVEDD
	China	45	32/13	64.17 ± 4.51	Control (B)	12			
Du et al. 2023	China	40	22/18	64.37 ± 3.22	RE	24	7	25	LVEF/6WMT/LVESD/LVEDD
	China	40	23/17	65.23 ± 3.11	MCET	24	7	25–50	
Jia 2018	China	58	31/27	53.64 ± 6.18	RE	12	7	25	LVEF/6WMT/LVESD/LVEDD
	China	58	32/26	63.7 ± 6.21	Control (B)	12			
Yang et al. 2022	China	57	37/20	56.03 ± 5.67	RE	12	3	60	LVEF/6MWT/LVESD/LVEDD
	China	56	35/21	55.50 ± 5.22	AE	12	7	Unclear	
Wu et al. 2017	China	30	26/4	60∼80	RE	24	3	50–60	6MWT
	China	34	27/7	60∼80	Control	24			
Zhu et al. 2023	China	35	18/17	60.22 ± 5.22	MBE	4	Unclear	Unclear	LVEF/6MWT
	China	35	19/16	60.11 ± 4.89	Control	4			
Yu et al. 2022	China	53	45/8	60.4 ± 11.37	MBE	24	7	30	LVEF/6MWT/LVEDD/LVESD
	China	53	44/9	61.13 ± 11.06	Control	24			
Gui 2022	China	55	35/20	57.50 ± 5.59	MBE	Unclear	7	30	LVEF/LVEDD/LVESD
	China	55	34/21	57.68 ± 5.64	Control	Unclear			
Chen et al. 2021	China	38	24/14	67.03 ± 7.26	MBE	12	4	20	6WMT/LVEF
	China	37	23/14	66.37 ± 7.45	Control	12			
Lu 2022	China	48	25/23	65.3 ± 2.45	MBE	12	3	15–30	6WMT/LVEF/LVEDD/LVESD
	China	48	27/21	66.07 ± 2.84	Control	12			
Deng et al. 2018	China	57	31/26	64.7 ± 4.2	MBE	24	5	40–50	6WMT/LVEF
	China	56	29/27	57.2 ± 4.9	Control	24			
Feng et al. 2021	China	60	38/22	60.1 ± 4.9	MBE	24	5	30–45	LVEF/6MWT/LVEDD
	China	60	35/25	60.5 ± 5.4	MCET	24	Unclear	Unclear	
liu 2023	China	45	32/10	55.38 ± 9.02	MBE	12	3–5	50	6MW/LVEF
	China	45	30/12	52.05 ± 9.81	AE	12	3–5	50	
Zhou et al. 2021	China	50	27/23	57.87 ± 4.61	MBE	12	7	30	6MWT/LVEF/LVEDD/LVESD
	China	50	31/19	58.41 ± 4.52	Control	12			
Li et al. 2018	China	53	28/25	61.27 ± 10.39	MBE	4	6	40	LVEF
	China	53	30/23	61.38 ± 10.21	Control	4			
Wu et al. 2023	China	60	34/26	55.38 ± 1.05	MBE	4	1–2	20–30	LVEF/LVEDD/LVESD
	China	60	35/25	55.35 ± 1.02	MCET	4	1–2	20–30	
Zong et al. 2022	China	50	23/27	57.26 ± 6.84	MBE	2	7	30	LVEF/LVEDD/LVESD
	China	50	21/29	56.91 ± 7.34	MCET	2	7	5–25	
Guo 2023	China	30	21/9	64.10 ± 5.58	MBE	12	14	25	LVEF/6MWT
	China	30	23/7	59.60 ± 5.78	Control	12			
Zhang et al. 2023	China	60	30/30	53.8 ± 14.2	MBE	12	3–5	40	LVEF
	China	60	30/30	52.4 ± 13.6	Control	12			
Liu 2023	China	44	23/21	67.24 ± 8.28	MBE	4	3–4	20	LVEF/LVEDD
	China	43	28/15	67.31 ± 8.34	Control	4			
Yao 2023	China	56	34/22	67.75 ± 7.13	MBE	24	7	30–60	LVEF/LVEDD/LVESD/6MWT
	China	56	32/24	67.43 ± 7.01	Control	24			
Ming-Gui Chen et al. 2020	China	43	29/14	59.98 ± 10.86	MBE	24	5	30	LVEF
	China	39	30/9	61.49 ± 11.54	Control	24			
Małgorzata Grabara et.al 2020	Poland	35	35/0	57.1 ± 5.3	MBE	6	7	30	LVEF/LVEDD/LVESD
	Poland	35	35/0	49.6 ± 5.03	Control	6			
Shi et al. 2022	China	103	76/27	65.27 ± 9.87	AE	12	7	20	LVEF/6MWD/LVEDD
	China	103	70/33	65.18 ± 9.91	Control	12			
Peng et al. 2023	China	65	26/29	48.64 ± 8.27	AE	24	10	30	LVEF/LVEDD/LVESD
	China	55	28/27	49.53 ± 8.25	Control	24			
Zhou et al. 2016	China	24	15/9	60.82 ± 8.37	AE	24	4–5	Unclear	LVEF/LVESD
	China	20	14/6	61.6 ± 8.71	Control	24			
Yan et al. 2021	China	41	22/19	60.03 ± 9.13	AE	Unclear	Unclear	Unclear	LVEF/6MWD/LVEDD/LVESD
	China	41	26/15	59.40 ± 7.66	Control	Unclear			
Zhou et al. 2017	China	25	9/16	54.82 ± 8.37	AE	12	4	20	LVEF/LVESD
	China	25	6/19	59.60 ± 8.71	Control	12			
Ji et al. 2017	China	36	20/16	50.9 ± 6.6	AE	12	3	30	LVEF/6MWD/LVESD
	China	40	26/14	50.8 ± 6.5	Control	12			
Zhou et al. 2021	China	61	34/27	57.62 ± 5.79	AE	12	10–14	40	LVEF/6MWT
	China	61	33/28	53.67 ± 5.93	Control	12			
Jiang et al. 2022	China	31	17/14	65.48 ± 3.29	AE	Unclear	4	20–30	LVEF/LVESD
	China	31	16/15	65.87 ± 3.33	Control	Unclear			
Chen et al. 2019	China	51	32/19	49.8 ± 6.5	AE	48	14	30	LVEF/LVESD
	China	51	31/20	49.7 ± 6.4	Control	48			
Yan et al. 2021	China	44	26/18	60.21 ± 8.93	AE	4	3	20	LVEF/LVESD
	China	44	27/17	60.39 ± 8.43	Control	4			
Hu et al. 2024	China	40	25/15	55.20 ± 5.84	AE	12	3	40	LVEF/LVEDD
	China	40	26/14	57.23 ± 6.32	Control	12			
Yao 2019	China	42	1.33	58.14 ± 6.43	AE	Unclear	14	10	LVEF
	China	42	23/19	58.36 ± 6.29	Control	Unclear			
Qin et al. 2023	China	54	29/25	67.46 ± 10.08	AE	24	3	30	LVEF/LVESD/LVESD/
	China	54	28/26	69.14 ± 10.13	Control	24			
Jiang et al. 2006	China	35	Unclear	Unclear	AE	12	5	20–30	LVEF/LVDD/
	China	29	Unclear	Unclear	Control	12			
Muthukrishnan et al. 2021	UAE	12	11/1	49 ± 8.43	AE	4	3	40–70	LVEF/6MWT
	UAE	12	11/1	48.41 ± 6.69	Control	4			
Gremeaux et al. 2011	French	8	7/1	65.8 ± 9	AE	6	3	80	6MWT/VO2peak
	French	9	7/2	59.2 ± 8.1	HIIT	6	3	80	
Zhang et al. 2022	China	42	24/18	55.24 ± 9.75	AE	4	3–5	20	LVEF/6MWT/LVEDD/LVESD
	China	42	26/16	54.91 ± 9.82	Control	4			
Bruna C.Matos-Garcia et al. 2022	Brazil	31	22/9	55.90 ± 14.60	AE	8	4	60	6MWT
	Brazil	23	17/6	55.80 ± 7.50	Control	8			
Thatiana C.A.Peixoto, MSc et al. 2015	Brazil	45	33/12	56.80 ± 10.80	AE	4	4	35–40	6MWT
	Brazil	43	29/14	56.00 ± 9.60	Control	4			
Cai et al. 2021	China	30	30/0	55 ± 9	AE	24	Unclear	Unclear	LVEF
	China	30	30/0	58 ± 8	Control	24			
Minghui Jiang et al. 2021	China	49	31/18	58.79 ± 9.36	MCET	24	7	Unclear	LVEF
	China	49	33/16	59.62 ± 8.98	Control	24			
Jonathan Myers et.al 2002	American	12	10/2	52.8 ± 12	AE	8	5	45	LVEF
	American	12	10/2	58.2 ± 6	Control	8			
Romualdo Belardinelli et.al 2001	American	59	49/10	53 ± 11	AE	24	3	30	LVEF/LVEDD/LVESD
	American	59	50/9	59 ± 10	Control	24			
FRANCESCO GIALLAURIA et.al 2006	Italy	20	16/4	68.6 ± 2.3	AE	12	3	30	LVEF
	Italy	20	17/3	68.2 ± 2.6	Control	12			
Francesco Giallauria et.al 2008	Italy	30	23/7	59 ± 3	AE	24	3	30	LVEF
	Italy	30	24/6	58 ± 4	Control	24			
Zheng et.al 2008	China	27	Unclear	Unclear	AE	24	3	30	LVEF
	China	30	Unclear	Unclear	Control	24			
Tomomi Koizumi et.al 2003	Japan	14	13/1	54 ± 12	AE	12	7	30	LVEF
	Japan	15	13/2	54 ± 12	Control	12			
Cha et al. 2016	China	40	27/13	46.26 ± 4.85	AE	12	3	30	LVEF
	China	40	25/15	46.35 ± 5.12	Control	12			
Fatemeh Basati et al. 2012	UAE	15	15/0	54.2 ± 9.04	AE	8	3	60–90	LVEF/LVEDD/LVESD
	UAE	14	14/0	51.71 ± 6.98	Control	8			
Mei 2022	China	42	23/19	55.18 ± 4.69	HIIT	24	3	45	LVEF/6MWT/LVEDD/LVESD
	China	42	20/22	56.39 ± 5.31	AE	24	7	30	
Yi et al. 2021	China	39	21/18	56. 38 ± 7.06	HIIT	12	3	40	LVEF/6MWT/LVEDD/LVESD
	China	31	18/13	55.63 ± 6.37	AE	12	7–21	5–30	
Zeng et al. 2024	China	45	22/23	63.02 ± 8.54	HIIT	12	7	40	LVEF/LVEDD/LVESD
	China	45	25/20	61.78 ± 9.05	AE	12	7	40	
	China	45	23/22	62.45 ± 8.37	Control	12			
Yoon Cho et al. 2018	MD	23	21/2	53 ± 6.84	HIIT	9	2	48	6MWT
	MD	21	18/3	57.31 ± 12.62	AE	9	2	48	
Antonello D'Andrea et.al 2022	Italy	75	43/32	62.3 ± 8.3	HIIT	8	2	Unclear	LVEF/LVEDD/LVESD
	Italy	50	30/20	59.3 ± 15.4	AE	8	2	Unclear	
Pu et al. 2017	China	41	0/41	73.7 ± 7.3	MCET	24	3–5	30–90	LVEF/6MWT
	China	48	0/48	70.09 ± 6.9	Control	24			LVEF/6MWT
Jiang et al. 2021	China	63	37/26	64.53 ± 5.39	MCET	Unclear	7	30–45	LVEF/6MWT
	China	63	35/28	64.09 ± 5.47	Control	Unclear			
Dong et al. 2023	China	55	34/21	55.67 ± 2.34	MCET	8	Unclear	30	LVEF/6MWT/LVEDD/LVESD
	China	55	35/20	55.69 ± 2.36	Control	8			
He et al. 2023	China	55	30/25	50.34 ± 12.08	MCET	24	14	30	LVEF/6MWT/LVEDD/LVESD
	China	51	28/23	48.63 ± 11.27	Control	24			
Wang 2022	China	40	24/16	58.94 ± 5.32	MCET	12	14	30	LVEF/6MWT/LVEDD/LVESD
	China	40	23/17	57.19 ± 5.47	AE	12	14	30	
Schmid et al. 2008	CH	17	15/2	54.7 ± 9.4	MCET	12	6	Unclear	LVEF
	CH	21	17/4	57 ± 9.6	AE	12	6	Unclear	
Chen et al. 2020	China	42	24/18	60.32 ± 7.11	MCET	24	7	40	LVEF
	China	42	26/16	60.39 ± 7.14	Control	24			
Tang et al. 2019	China	30	19/11	67.25 ± 3.15	MCET	Unclear	3	50	LVEF
	China	30	17/13	68.23 ± 2.13	AE	Unclear	3	50	
	China	30	21/9	68.35 ± 3.03	Control	Unclear			
Feng 2023	China	56	29/27	56.18 ± 4.67	RE	12	14–21	Unclear	LVEF/LVEDD/LVESD
	China	56	30/26	55.67 ± 4.48	AE	12	7	Unclear	
Xu et al. 2022	China	37	20/17	54.28 ± 5.43	RE	12	14–21	50	LVEF/LVEDD/LVESD
	China	37	22/15	54.35 ± 5.35	AE	12	3–4	30	
Chen et al. 2024	China	43	24/19	68.22 ± 4.41	MCET	12	2–4	10–25	LVEF/6MWT/LVEDD/LVESD
	China	43	25/18	67.69 ± 4.52	Control	12			
Ye 2023	China	30	21/9	52.44 ± 3.16	MCET	12	7	30–50	LVEF/LVEDD/LVESD
	China	30	23/7	52.28 ± 4.04	Control	12			

AE, aerobic exercise; RE, resistance exercise; HIIT, high-intensity interval training; MBE, mind-body exercise; MCET, multi-component exercise; 6MWT, 6-minute walk test; LVEF, left ventricular ejection fraction; LVEDD, left ventricular end-diastolic diameter; LVESD, left ventricular end-systolic diameter.

The detailed information and citations of each study are shown in Supplementary Appendices 4 and 10.

### 3.2 Risk of bias

**Figure 2:**
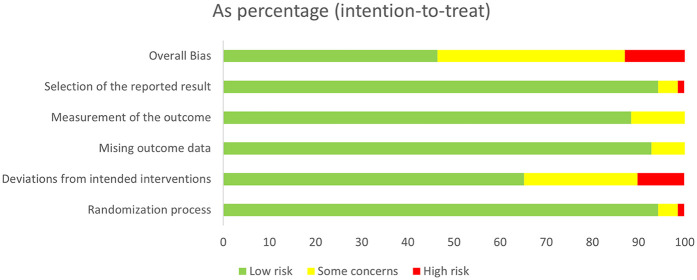
Risk of bias.

Figure 2 shows the overall risk of bias for the included studies. 33 studies were assessed as having low risk, 28 studies as having some risk, and 8 studies as having high risk. Among all the domains assessed, deviation from the intended interventions was the primary factor influencing study quality. Detailed information on each study's risk of bias in the various domains is presented in Supplementary Appendix 5.

### 3.3 Network meta-analysis

**Figure 3:**
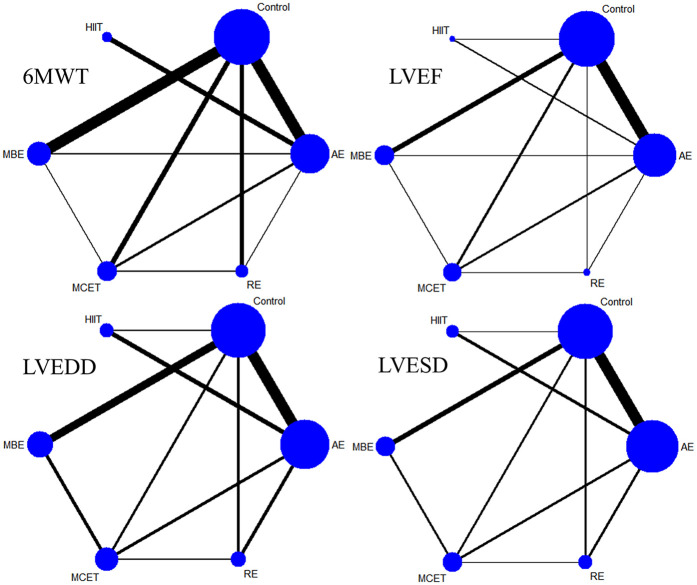
Network plot. AE, aerobic exercise; MBE, mind-body exercise; HIIT, high-intensity interval training; MCET, multi-component exercise; RE, resistance exercise; 6MWT, 6-minute walk test; LVEF, left ventricular ejection fraction; LVEDD, left ventricular end-diastolic diameter; LVESD, left ventricular end-systolic diameter.

**Figure 4:**
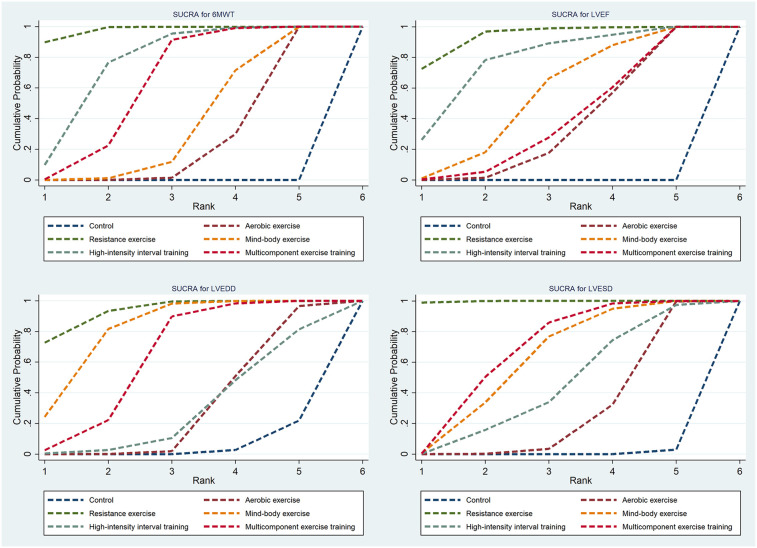
Cumulative ranking probability plots.

Thirty-three studies reported the 6MWT outcomes, involving 2,613 participants. Figure 3 presents the comparisons between different interventions, with global inconsistency tests indicating no significant inconsistency (*P* > 0.05). The NMA showed that, compared to the control group, all exercise interventions significantly improved the 6MWT scores in MI patients. The MDs (95% CI) ranged from 57.61 (34.87, 80.36) for AE to 144.38 (110.78, 177.98) for RE (low to moderate evidence quality). Additionally, RE demonstrated significantly greater efficacy than mind-body exercise (MD: 77.83, 95% CI: 37.38, 118.28, low evidence quality) and MCET (MD: 54.16, 95% CI: 15.18, 93.13, low evidence quality). The efficacy of AE was significantly worse than that of RE (MD: −86.76, 95% CI: −123.84, −49.69, very low evidence quality), HIIT (MD: −51.35, 95% CI: −90.95, −11.76, very low evidence quality), and MCET (MD: −32.60, 95% CI: −63.87, −1.34, low evidence quality) (Table 2, Supplementary Appendix 9.4). The probability-based ranking provided by the SUCRA analysis positioned RE as the modality most likely to be the most effective (SUCRA: 97.9), followed by HIIT (SUCRA: 76.2) and MCET (SUCRA: 62.7) (Table 3; Figure 4). Network meta-regression analysis indicated that the average age of participants and intervention duration may influence the efficacy of RE and HIIT, which lowered the quality of evidence for these comparisons (Supplementary Appendix 9).

**Table 2:** Network meta-analysis results for each outcome.

Outcome	Comparison					
6MWT	AE					
	**−86.76** **(−123.84,−49.69)**	RE				
	−8.93 (−39.85,21.99)	**77.83** (**37.38,118.28)**	MBE			
	**−51.35** (**−90.95,−11.76)**	35.41 (−18.83,89.65)	−42.42 (−92.66,7.81)	HIIT		
	**−32.60** (**−63.87,−1.34)**	**54.16** (**15.18,93.13)**	−23.67 (−57.58,10.23)	18.75 (−31.69,69.19)	MCET	
	**57.61** (**34.87,80.36)**	**144.38** (**110.78,177.98)**	**66.55** (**42.66,90.43)**	**108.97** (**63.31,154.63)**	**90.22 (62.66,117.78)**	Control
LVEF	AE					
	**−3.99** (**−6.99,−1.00)**	RE				
	−0.84 (−2.95,1.27)	3.15 (−0.28,6.58)	MBE			
	−2.61 (−6.09,0.86)	1.38 (−3.18,5.94)	−1.77 (−5.78,2.23)	HIIT		
	−0.10 (−2.21,2.01)	**3.89** (**0.56,7.22)**	0.74 (−1.61,3.09)	2.51 (−1.51,6.53)	MCET	
	**4.75** (**3.42,6.09)**	**8.75** (**5.72,11.77)**	**5.60** (**3.84,7.35)**	**7.37** (**3.72,11.02)**	**4.86 (2.89,6.82)**	Control
LVEDD	AE					
	**4.86** (**2.10,7.61)**	RE				
	**3.69** (**1.00,6.38)**	−1.17 (−4.56,2.22)	MBE			
	−0.05 (−3.09,3.00)	**−4.91** (**−8.96,−0.86)**	−3.74 (−7.68,0.21)	HIIT		
	2.47 (−0.07,5.00)	−2.39 (−5.61,0.82)	−1.22 (−3.83,1.38)	2.51 (−1.37,6.40)	MCET	
	−1.54 (−3.45,0.37)	**−6.40** (**−9.24,−3.56)**	**−5.23** (**−7.36,−3.10)**	−1.49 (−4.94,1.95)	**−4.01 (−6.42,−1.59)**	Control
LVESD	AE					
	**5.44** (**3.21,7.66)**	RE				
	1.75 (−0.48,3.98)	**−3.69** (**−6.55,−0.82)**	MBE			
	0.78 (−1.70,3.27)	**−4.65** (**−7.95,−1.35)**	−0.97 (−4.23,2.29)	HIIT		
	1.98 (−0.04,4.01)	**−3.45** (**−6.08,−0.82)**	0.23 (−2.10,2.57)	1.20 (−1.96,4.36)	MCET	
	**−1.89** (**−3.27,−0.51)**	**−7.33** (**−9.62,−5.03)**	**−3.64** (**−5.52,−1.76)**	−2.67 (−5.41,0.06)	**−3.87 (−5.87,−1.88)**	Control

AE, aerobic exercise; MBE, mind-body exercise; HIIT, high-intensity interval training; MCET, multi-component exercise; RE, resistance exercise; 6MWT, 6-minute walk test; LVEF, left ventricular ejection fraction; LVEDD, left ventricular end-diastolic diameter; LVESD, left ventricular end-systolic diameter. Bold font indicates significant differences.

**Table 3:** SUCRA values for each exercise.

Treatment	Outcome			
	6MWT	LVEF	LVEDD	LVESD
Control	0	0	4.9	0.6
AE	26.3	35.2	29.9	27.1
RE	97.9	93.7	93.2	99.8
MBE	36.9	54.7	80.8	61.2
HIIT	76.2	77.7	28.7	44.3
MCET	62.7	38.8	62.5	67

AE, aerobic exercise; MBE, mind-body exercise; HIIT, high-intensity interval training; MCET, multi-component exercise; RE, resistance exercise; 6MWT, 6-minute walk test; LVEF, left ventricular ejection fraction; LVEDD, left ventricular end-diastolic diameter; LVESD, left ventricular end-systolic diameter.

Sixty-four studies reported LVEF, involving 4,919 participants. The network plots of comparisons between different interventions are shown in Figure 3, with no significant global inconsistency (*P* > 0.05). Compared to the control group, all exercise interventions improved LVEF in MI patients, with MDs (95% CI) ranging from 4.75 (3.42, 6.09) for AE to 8.75 (5.72, 11.77) for RE (very low to moderate evidence quality). Furthermore, RE showed significantly greater efficacy than MCET (MD: 3.89, 95% CI: 0.56, 7.22, moderate evidence quality) and AE (MD: −3.99, 95% CI: −6.99, −1.00, low evidence quality), while no significant differences were found between other intervention pairs (Table 2, Supplementary Appendix 9.4). The probabilistic SUCRA analysis was consistent with these findings, indicating that RE had the highest probability of being the most effective intervention (SUCRA: 93.7), followed closely by HIIT (SUCRA: 77.7) and mind-body exercise (SUCRA: 54.7) (Table 3; Figure 4). Regression analysis suggested that baseline severity might be a potential factor influencing RE efficacy (Supplementary Appendix 9).

Thirty-three studies reported LVEDD, involving 2,818 participants. The network plot is shown in Figure 3. Compared to the control group, all interventions, except AE and HIIT, significantly reduced LVEDD in MI patients. Specifically, RE (MD: −6.40, 95% CI: −9.24, −3.56, moderate evidence quality), mind-body exercise (MD: −5.23, 95% CI: −7.36, −3.10, very low evidence quality), and MCET (MD: −4.01, 95% CI: −6.42, −1.59, very low evidence quality) were significantly effective in reducing LVEDD. AE was less effective than RE (MD: 4.86, 95% CI: 2.10, 7.61, low evidence quality) and mind-body exercise (MD: 3.69, 95% CI: 1.00, 6.38, low evidence quality), while RE was significantly more effective than MCET (MD: 3.89, 95% CI: 0.56, 7.22, moderate evidence quality) (Table 2, Supplementary Appendix 9.4). The SUCRA rankings reflected this, with RE (SUCRA: 93.2), mind-body exercise (SUCRA: 80.8) and MCET (SUCRA: 62.5) having relatively high probabilities of being effective interventions for promoting favorable diastolic remodeling (Table 3; Figure 4). Regression analysis suggested that participant age and intervention duration may influence the efficacy of mind-body exercise and MCET (Supplementary Appendix 9).

Thirty-four studies reported LVESD, involving 2,683 participants. The network plot in Figure 3 depicts the comparisons between interventions. The results show that all exercise interventions, except HIIT, were significantly more effective than the control group for improving LVESD in MI patients, with MDs (95% CI) ranging from −1.89 (−3.27, −0.51) for AE to −7.33 (−9.62, −5.03) for RE (very low to moderate evidence quality). Additionally, RE demonstrated greater efficacy compared to AE (MD: 5.44, 95% CI: 3.21, 7.66, low evidence quality), mind-body exercise (MD: −3.69, 95% CI: −6.55, −0.82, moderate evidence quality), HIIT (MD: −4.65, 95% CI: −7.95, −1.35, low evidence quality), and MCET (MD: −3.45, 95% CI: −6.08, −0.82, moderate evidence quality) (Table 2, Supplementary Appendix 9.4). The SUCRA value for RE (99.8) further corroborated its high probability of being a highly effective intervention. MCET (SUCRA: 67.0) and mind-body exercise (SUCRA: 61.2) were ranked as the next potentially effective interventions, although their effect sizes were considerably smaller than that of RE (Table 3; Figure 4). Regression analysis suggested that baseline severity and participant age might be potential factors influencing the efficacy of RE and mind-body exercise (Supplementary Appendix 9).

### 3.4 Sensitivity analysis

To assess the robustness of our findings, we performed a sensitivity analysis by excluding studies with a high risk of bias, which was primarily due to deviations from intended interventions. The results of this analysis were largely consistent with the primary analysis, although the effect estimates for some between-intervention comparisons were slightly attenuated. This suggests that our main conclusions are robust, even with the inclusion of these higher-risk studies (full results are available in Supplementary Appendix 7).

### 3.5 Publication bias

Visual inspection of the comparison-adjusted funnel plots for 6MWT, LVEF, LVEDD, and LVESD revealed a relatively symmetrical distribution of study points, indicating no strong evidence of significant publication bias across the network (funnel plots are shown in Supplementary Appendix 8).

## 4 Discussion

Exercise-based interventions for improving cardiac function in MI patients are widely recognized as beneficial for cardiac function (16). This study is the first to use network meta-analysis to systematically review eligible studies and determine the effects of mind-body exercise, AE, RE, HIIT, and MCET on cardiac function in MI patients. Additionally, regression analysis was employed to explore potential factors influencing exercise efficacy. The aim is to provide a reference for decision-making in exercise interventions within CR for this population. The findings suggest that all types of exercise interventions are likely to effectively improve cardiac function in MI patients, with RE appearing to have a high probability of being a particularly effective intervention for enhancing 6MWT, LVEF, LVEDD, and LVESD. Additionally, HIIT showed notable improvements in 6MWT and LVEF. For LVEF and LVEDD, mind-body exercise was found to be the next most effective after RE.

### 4.1 The effect of different exercise modalities on cardiac function

A noteworthy finding of this study is the potential superiority of RE over traditional aerobic training in improving the 6MWT distance. While this phenomenon may not be universally applicable, it underscores the necessity of personalized rehabilitation, the core mechanism of which lies in precisely targeting the “weak links” that limit functional capacity in specific patient subgroups. Specifically, for many post-MI patients who are elderly, frail, or have significant sarcopenia, the bottleneck for their functional capacity has shifted from the central cardiopulmonary system to the peripheral skeletal muscle system (41). In these individuals, exercise cessation is often due to lower limb muscle fatigue, insufficient strength, or poor balance, rather than reaching the limits of their cardiopulmonary endurance. RE directly addresses this fundamental peripheral limiting factor by enhancing muscle mass, strength, and neuromuscular coordination (42). This perspective is supported by the findings of a recent network meta-analysis, which not only observed an overall advantage of RE in improving the 6MWT but also revealed through its meta-regression analysis that this advantage was particularly pronounced in older patients or those with poorer baseline functional status (43). The benefits of RE on LVEF and LVESD may be more apparent in study populations with higher baseline disease severity, whereas its impact on the 6MWT is influenced by participant age (44). This finding resonates strongly with the evolving clinical paradigm for frail individuals in post-MI rehabilitation, a population often characterized by advanced age, sarcopenia, and more severe cardiac dysfunction. For these patients, a “resistance-first” approach is increasingly advocated (45). Therefore, it is crucial to emphasize that the “superiority” of RE is conditional; its value does not negate the cornerstone status of aerobic exercise in cardiac rehabilitation but rather positions it as a vital synergistic or preparatory strategy. By strengthening the peripheral musculature first, it not only directly enhances patients' functional performance and sense of security but also lays a solid foundation for them to subsequently engage in more effective and beneficial aerobic training (42). Furthermore, this result reflects the comprehensive nature of the 6MWT as a functional assessment tool. Performance in the 6MWT is not solely dependent on cardiovascular endurance but is also significantly influenced by peripheral factors such as lower limb muscle strength, walking economy, and patient self-efficacy (46). For untrained or frail patients, peripheral muscle fatigue often becomes a limiting factor earlier than For untrained or frail patients, peripheral muscle fatigue often becomes a limiting factor in submaximal exercise earlier than cardiac output (47). By precisely ameliorating this limitation, RE leads to a significant increase in walking distance, which is sensitively captured by the functional endpoint of the 6MWT (48). In terms of assessment methods, although Cardiopulmonary Exercise Testing (CPET) and its parameters (e.g., peak oxygen uptake, VO₂peak; first ventilatory threshold, VT1) represent the “gold standard” for evaluating physiological adaptations, their limited availability in primary care settings restricts their widespread clinical application (49). While the 6MWT may be less sensitive, its simplicity and ease of implementation render it more valuable in real-world clinical practice. Due to the lack of consistent CPET data reporting in the included literature, our study was unable to analyze these more precise physiological indicators, which highlights a direction for future research.

Beyond its direct effects on peripheral muscles, RE also exerts positive influences on the heart itself, consistent with previous research. Studies have demonstrated that RE can enhance cardiac function by improving myocardial contractility, autonomic nervous function, and neuro-cardiovascular stress responses (50). Mechanistically, RE helps improve diastolic function, reduce left ventricular stiffness and filling pressure (51), and positively influences post-MI cardiac remodeling without inducing adverse left ventricular dilation (52). Moreover, by increasing cardiac pressure load, RE can improve subendocardial blood perfusion and decrease myocardial oxygen consumption, thereby alleviating myocardial ischemia (53).These multifaceted benefits collectively support the view that RE should be a core component of comprehensive management in post-MI cardiac rehabilitation.

HIIT has garnered significant attention for its effectiveness in improving patients' LVEF and exercise tolerance. Previous studies have shown that HIIT can mitigate adverse cardiac remodeling and enhance myocardial contractile function by improving glucose and lipid metabolism, reducing oxidative stress, and inhibiting myocardial fibrosis and apoptosis (18, 54, 55). Recent research has further revealed that HIIT can activate the mechano-growth factor (MGF)-related signaling pathway, thereby reducing infarct size and improving cardiac function (56). However, the choice of exercise intensity and modality is critical. Exhausting exercise may impair myocardial contractile function (57, 58), whereas moderate-intensity interval exercise can improve both systolic and diastolic function by optimizing the kinetics of cardiomyocyte calcium transients (59, 60).

Our study found that compared to AE, HIIT, and multicomponent exercise, mind-body exercise demonstrated a superior effect in improving LVEDD and LVESD. This suggests that mind-body exercise, as an effective rehabilitation therapy, may improve cardiac structure and function in MI patients through its unique mechanisms. Mind-body exercise emphasizes the integration of mind and body, promoting physical and mental relaxation and improving myocardial blood supply and oxygenation through coordinated physical movements, rhythmic breathing control, and mental focus (11). Its gentle, rhythmic motions may help optimize diastolic filling efficiency, thereby improving cardiac function and exercise tolerance. Notably, AE did not significantly reduce LVEDD in our analysis. One possible explanation is that the AE protocols in the included studies failed to reach the stimulatory threshold in intensity or duration required to induce beneficial cardiac remodeling (61). Cardiac reverse remodeling is a long-term process that requires a sufficient and sustained stimulus. Another explanation involves the distinct hemodynamic effects of different exercise modalities (45). AE primarily imposes a sustained volume load, whereas other effective interventions (such as resistance or multicomponent training) may confer benefits through different mechanisms (62). For instance, these modalities, by increasing skeletal muscle mass and improving peripheral vascular function, may lead to a long-term reduction in systemic vascular resistance, i.e., a decrease in cardiac afterload. A reduction in afterload is a potent stimulus for decreasing left ventricular dimensions, an effect that may have been less pronounced with the AE protocols analyzed in our study, possibly explaining its non-significant impact on LVEDD. Our results highlight the potential of mind-body exercise and AE as adjunct therapies in cardiac rehabilitation for MI patients, particularly given their safety and convenience. However, definitive conclusions cannot be drawn at this stage due to limitations in the intervention protocols, heterogeneity, and sample sizes of existing studies. Future large-scale, rigorously designed randomized controlled trials are urgently needed to further validate the clinical benefits of mind-body exercise and AE.

### 4.2 Analysis of sources of heterogeneity

To investigate the potential sources of heterogeneity in our findings, we conducted a network meta-regression analysis (see Supplementary Appendix 7.3). The results revealed that baseline disease severity was a key moderator of the efficacy of RE in improving LVEF and LVESD. Furthermore, the mean age of participants moderated the effect of mind-body exercise on LVEDD and LVESD, as well as the impact of RE on the 6MWT. These findings underscore the importance of comprehensive baseline assessments (including medical history, physical examination, and electrocardiogram) in future studies. This is not only crucial for ensuring the homogeneity of the study population but is also a prerequisite for ensuring the safety of exercise interventions (63). The meta-regression analysis also indicated that the “duration” of the intervention was another significant factor influencing the efficacy of HIIT and MCET on the 6MWT and LVEDD outcomes. The data showed that the intervention period for HIIT was relatively short (mean 11.8 weeks), suggesting that improving cardiac function and exercise tolerance in MI patients through exercise may be a long-term process requiring a longer duration.

The heterogeneity in this study may also stem from the variability in control group interventions. We found that the control groups in the vast majority of studies employed a mixture of various interventions, making a clear, non-overlapping classification extremely difficult. Although we attempted a more granular stratification of the control groups, for example, by creating a separate subgroup for studies using only pharmacological treatment (64, 65), the sample size of such subgroups was too small, leading to insufficient statistical power and potentially misleading results. This inherent variability in control conditions inevitably affects the precise estimation of the relative efficacy of each active intervention, thereby reducing the certainty of our study's conclusions. Additionally, the vast majority of studies (*n* = 60) explicitly stated that interventions were supervised. Although the remaining nine studies did not specify supervision status, their hospital or rehabilitation center settings suggest that supervised implementation was highly probable (Supplementary Appendix 4). These methodological ambiguities collectively point to an urgent need: future clinical trials in cardiac rehabilitation must precisely define and report the components of control group interventions to facilitate more robust and meaningful evidence synthesis.

The primary source of bias in the included studies was the risk of bias related to deviations from intended interventions, along with the failure to transparently report the random sequence generation process. We conducted a sensitivity analysis by excluding studies with a high risk of bias. The results indicated that the significance of comparisons between different exercise interventions for all outcomes remained unchanged, and their relative ranking of superiority remained stable. This finding from the sensitivity analysis suggests that the core conclusions of our study were not unduly influenced by a few studies of questionable methodological quality. It reflects a degree of consistency and internal homogeneity within the existing body of evidence, where studies from different settings and with varying designs converge towards a clear and robust conclusion. Future research should still aim to optimize current methodological weaknesses, such as reporting of random sequences, standardization of intervention delivery, and blinding of subjective outcomes, as well as increase sample sizes and geographical coverage to further enhance evidence quality and generalizability.

### 4.3 Limitations

This study has several limitations: (1) although the diagnosis of myocardial infarction in each study was clinically valid, we were unable to assess the differential effects of various exercise modalities on patients with different types of myocardial infarction (e.g., STEMI and NSTEMI) due to insufficient reporting in the original studies. This clinical heterogeneity could affect patient prognosis and response to exercise. (2) The risk of bias, particularly that related to deviations from intended interventions, was a concern in many of the included studies. Although the sensitivity analysis indicated the robustness of our pooled results, the cumulative effect of these biases may have slightly inflated the comparative advantages of some interventions. This potential influence should be considered when interpreting the results and highlights the need for more standardized implementation and reporting in future research. (3) There was considerable heterogeneity in the implementation of exercise interventions across the included studies. For instance, many studies used only qualitative descriptions such as “moderate intensity” or “conventional rehabilitation training” without providing quantifiable parameters like target heart rate zones, percentage of maximal oxygen uptake, or ratings of perceived exertion. This precluded subgroup analyses and network meta-regression based on high- vs. low-to-moderate-intensity interventions. While this issue appears to be common in the field, as observed in similar high-quality studies (32, 66), the unmeasured variability in intensity should be considered with caution when interpreting the results. Future studies could consider using MET intensities to quantify the dose of different exercise interventions for more precise comparisons (67). (4) Although we employed a global search strategy, the included RCTs were predominantly from studies published in China. While this objectively reflects the current distribution of research in this field, it may limit the external validity and global generalizability of our conclusions. Readers should interpret the findings in the context of local clinical practices and population characteristics when extrapolating them to other regions or populations.These limitations somewhat diminish the confidence in the quality of the evidence. Therefore, future research should target these qaps by refining subtype-specific analyses,standardizing quantitative reporting of exercise interventions, and expanding data from diverse regions toenhance the reliability and clinical applicability of evidence in this field.

## 5 Conclusion

This network meta-analysis evaluated the effects of five different exercise interventions on cardiac function in patients after myocardial infarction. Our findings suggest that RE may be the most effective single modality for improving cardiac function and promoting favorable remodeling post-MI. Its benefits may be particularly pronounced in older or more severely deconditioned patients, supporting a “resistance-first” rehabilitation strategy for this vulnerable population. While MCET and mind-body exercise also offer significant advantages, particularly in reducing ventricular size, claims of any single modality's superiority should be interpreted with caution. The choice of exercise must be individualized, considering the variable quality of evidence for certain comparisons, the patient's specific clinical status, functional capacity, and personal preferences. Ultimately, healthcare professionals should use these findings to guide the design of more tailored and effective rehabilitation programs, moving towards a more personalized exercise prescription approach for post-MI patients.

## Data Availability

The raw data supporting the conclusions of this article will be made available by the authors, without undue reservation.
