# Supplementary material for: Which exercise modality is most effective for improving cardiac function in patients with myocardial infarction? A network meta-analysis
Source: Front Cardiovasc Med. 2025 Oct 30;12:1623727. doi: 10.3389/fcvm.2025.1623727 (PMC12611906; doi:10.3389/fcvm.2025.1623727)
Supplement: Supplementary file 1 [file Supplementaryfile1.docx]

Contents

[Appendix 1. PRISMA-NMA Checklist 1](#_Toc22868)

[Appendix 2. Search Strategy 5](#_Toc1055)

[Appendix 3. Definition of exercise type and control group 13](#_Toc22310)

[Appendix 4. Detailed characteristics of the included studies 14](#_Toc15047)

[Appendix 5. Summary of risk of bias 49](#_Toc13557)

[Appendix 6. Local inconsistency test 50](#_Toc26025)

[Appendix 7. Sensitivity Analysis 52](#_Toc2694)

[Appendix 8. Comparison-adjusted funnel plots 53](#_Toc30967)

[Appendix 9. Assessment of evidence quality 54](#_Toc20665)

[Appendix 9.1. Description of the assessment criteria for CINeMA 54](#_Toc29090)

[Appendix 9.2. Network plot with risk of bias 55](#_Toc8713)

[Appendix 9.3. Results of the network meta-regression 56](#_Toc29788)

[Appendix 9.4. Details of evidence quality assessment 57](#_Toc15355)

[Appendix 10. Citation information for included studies 61](#_Toc11724)

Appendix 1. PRISMA-NMA Checklist

| **Section/Topic** | **Item #** | **Checklist Item** | **Reported on Page #** |
| --- | --- | --- | --- |
| **TITLE** |  |  |  |
| Title | 1 | Identify the report as a systematic review *incorporating a network meta-analysis (or related form of meta-analysis).* | Title |
|  |  |  |  |
| **ABSTRACT** |  |  |  |
| Structured summary | 2 | Provide a structured summary including, as applicable:  **Background:** main objectives  **Methods:** data sources; study eligibility criteria, participants, and interventions; study appraisal; and *synthesis methods, such as network meta-analysis.*  **Results:** number of studies and participants identified; summary estimates with corresponding confidence/credible intervals; *treatment rankings may also be discussed. Authors may choose to summarize pairwise comparisons against a chosen treatment included in their analyses for brevity.*  **Discussion/Conclusions:** limitations; conclusions and implications of findings.  **Other:** primary source of funding; systematic review registration number with registry name. | Abstract |
|  |  |  |  |
| **INTRODUCTION** |  |  |  |
| Rationale | 3 | Describe the rationale for the review in the context of what is already known*, including mention of why a network meta-analysis has been conducted.* | ***1. Introduction*** |
| Objectives | 4 | Provide an explicit statement of questions being addressed, with reference to participants, interventions, comparisons, outcomes, and study design (PICOS). | 1. Introduction |
|  |  |  |  |
| **METHODS** |  |  |  |
| Protocol and registration | 5 | Indicate whether a review protocol exists and if and where it can be accessed (e.g., Web address); and, if available, provide registration information, including registration number. | 2. Methods |
| Eligibility criteria | 6 | Specify study characteristics (e.g., PICOS, length of follow-up) and report characteristics (e.g., years considered, language, publication status) used as criteria for eligibility, giving rationale. *Clearly describe eligible treatments included in the treatment network, and note whether any have been clustered or merged into the same node (with justification).* | ***2.2. Inclusion Criteria*** |
| Information sources | 7 | Describe all information sources (e.g., databases with dates of coverage, contact with study authors to identify additional studies) in the search and date last searched. | 2.1. Search Strategy |
| Search | 8 | Present full electronic search strategy for at least one database, including any limits used, such that it could be repeated. | Appendix 2 |
| Study selection | 9 | State the process for selecting studies (i.e., screening, eligibility, included in systematic review, and, if applicable, included in the meta-analysis). | 2.3. Study Selection and Data Collection |
| Data collection process | 10 | Describe method of data extraction from reports (e.g., piloted forms, independently, in duplicate) and any processes for obtaining and confirming data from investigators. | 2.3. Study Selection and Data Collection |
| Data items | 11 | List and define all variables for which data were sought (e.g., PICOS, funding sources) and any assumptions and simplifications made. | 2.2. Inclusion Criteria, Appendix 3 |
| **Geometry of the network** | **S1** | Describe methods used to explore the geometry of the treatment network under study and potential biases related to it. This should include how the evidence base has been graphically summarized for presentation, and what characteristics were compiled and used to describe the evidence base to readers. | ***2.5. Statistical Analysis*** |
| Risk of bias within individual studies | 12 | Describe methods used for assessing risk of bias of individual studies (including specification of whether this was done at the study or outcome level), and how this information is to be used in any data synthesis. | 2.4. Risk of Bias and Quality of Evidence Assessment |
| Summary measures | 13 | State the principal summary measures (e.g., risk ratio, difference in means). *Also describe the use of additional summary measures assessed, such as treatment rankings and surface under the cumulative ranking curve (SUCRA) values, as well as modified approaches used to present summary findings from meta-analyses.* | 2.5. Statistical Analysis |
| Planned methods of analysis | 14 | Describe the methods of handling data and combining results of studies for each network meta-analysis. This should include, but not be limited to:   - *Handling of multi-arm trials;* - *Selection of variance structure;* - *Selection of prior distributions in Bayesian analyses; and* - *Assessment of model fit.* | 2.5. Statistical Analysis |
| **Assessment of Inconsistency** | **S2** | Describe the statistical methods used to evaluate the agreement of direct and indirect evidence in the treatment network(s) studied. Describe efforts taken to address its presence when found. | 2.5. Statistical Analysis |
| Risk of bias across studies | 15 | Specify any assessment of risk of bias that may affect the cumulative evidence (e.g., publication bias, selective reporting within studies). | **2.4. Risk of Bias and Quality of Evidence Assessment** |
| Additional analyses | 16 | Describe methods of additional analyses if done, indicating which were pre-specified. This may include, but not be limited to, the following:   - Sensitivity or subgroup analyses; - Meta-regression analyses; - *Alternative formulations of the treatment network; and* - *Use of alternative prior distributions for Bayesian analyses (if applicable).* | ***2.5. Statistical Analysis*** |
|  |  |  |  |
| **RESULTS†** |  |  |  |
| Study selection | 17 | Give numbers of studies screened, assessed for eligibility, and included in the review, with reasons for exclusions at each stage, ideally with a flow diagram. | 3.1. Characteristics of Included Studies |
| **Presentation of network structure** | **S3** | Provide a network graph of the included studies to enable visualization of the geometry of the treatment network. | ***Figure 1*** |
| **Summary of network geometry** | **S4** | Provide a brief overview of characteristics of the treatment network. This may include commentary on the abundance of trials and randomized patients for the different interventions and pairwise comparisons in the network, gaps of evidence in the treatment network, and potential biases reflected by the network structure. | ***3.3. Network Meta-Analysis*** |
| Study characteristics | 18 | For each study, present characteristics for which data were extracted (e.g., study size, PICOS, follow-up period) and provide the citations. | Table 1, Appendix 4 |
| Risk of bias within studies | 19 | Present data on risk of bias of each study and, if available, any outcome level assessment. | 3.2. Risk of Bias, Appendix 5 |
| Results of individual studies | 20 | For all outcomes considered (benefits or harms), present, for each study: 1) simple summary data for each intervention group, and 2) effect estimates and confidence intervals. *Modified approaches may be needed to deal with information from larger networks.* | ***Table 1, Appendix 4*** |
| Synthesis of results | 21 | Present results of each meta-analysis done, including confidence/credible intervals. *In larger networks, authors may focus on comparisons versus a particular comparator (e.g. placebo or standard care), with full findings presented in an appendix. League tables and forest plots may be considered to summarize pairwise comparisons.* If additional summary measures were explored (such as treatment rankings), these should also be presented. | ***3.3. Network Meta-Analysis, Appendix 7*** |
| **Exploration for inconsistency** | **S5** | Describe results from investigations of inconsistency. This may include such information as measures of model fit to compare consistency and inconsistency models, *P* values from statistical tests, or summary of inconsistency estimates from different parts of the treatment network. | ***3.3. Network Meta-Analysis, Appendix 6*** |
| Risk of bias across studies | 22 | Present results of any assessment of risk of bias across studies for the evidence base being studied. | 3.2. Risk of Bias, Appendix 5 |
| Results of additional analyses | 23 | Give results of additional analyses, if done (e.g., sensitivity or subgroup analyses, meta-regression analyses*, alternative network geometries studied, alternative choice of prior distributions for Bayesian analyses,* and so forth). | 3.4. Sensitivity Analysis, Appendix 9.3 |
|  |  |  |  |
| **DISCUSSION** |  |  |  |
| Summary of evidence | 24 | Summarize the main findings, including the strength of evidence for each main outcome; consider their relevance to key groups (e.g., healthcare providers, users, and policy-makers). | 4. Discussion |
| Limitations | 25 | Discuss limitations at study and outcome level (e.g., risk of bias), and at review level (e.g., incomplete retrieval of identified research, reporting bias). *Comment on the validity of the assumptions, such as transitivity and consistency. Comment on any concerns regarding network geometry (e.g., avoidance of certain comparisons).* | 4. Discussion |
| Conclusions | 26 | Provide a general interpretation of the results in the context of other evidence, and implications for future research. | 5. Conclusion |
|  |  |  |  |
| **FUNDING** |  |  |  |
| Funding | 27 | Describe sources of funding for the systematic review and other support (e.g., supply of data); role of funders for the systematic review. This should also include information regarding whether funding has been received from manufacturers of treatments in the network and/or whether some of the authors are content experts with professional conflicts of interest that could affect use of treatments in the network. | ***Funding*** |

Appendix 2. Search Strategy

| Databases | Terms |
| --- | --- |
| The Cochrane Library | #1 MeSH descriptor: [Myocardial Infarction] explode all trees  #2 ("Infarct, Myocardial" OR "Cardiovascular Strokes" OR "Infarction, Myocardial" OR "Myocardial Infarcts" OR "Strokes, Cardiovascular" OR " Heart Attack" OR "Stroke, Cardiovascular" OR "Heart Attacks" OR "Infarcts, Myocardial" OR "Infarctions, Myocardial" OR "Myocardial Infarctions" OR "Cardiovascular Stroke" OR "Myocardial Infarct"):ti,ab,kw  #3 ("Resistance training" or "Training, Resistance" or "Strength Training" or "Training, Strength" or "Weight-Lifting Strengthening Program" or "Strengthening Program,Weight-Lifting" or "Strengthening Programs,Weight-Lifting" or "Weight LiftingStrengtheningProgram" or "Weight Lifting Strengthening Programs" or "Weight-Lifting Exercise Program" or "Exercise Program,Weight-Lifting" or "Exercise Programs, Weight-Lifting" or "Weight LiftingExerciseProgram" or "Weight-Lifting Exercise Programs" or "Weight-Bearing Strengthening Program" or "Strengthening Program, Weight-Bearing" or "Strengthening Programs, Weight-Bearing" or "Weight Bearing Strengthening Program" or "Weight-Bearing Strengthening Programs" or "Weight-Bearing Exercise Program"):ti,ab,kw  #4 ("mind-body exercises" or "Tai Chi" or "Tai Ji" or "Tai Chi Exercise" or "Tai Ji Chuan" or "Tai Ji Quan" or "Tai Chi Chuan" or "Tai Chi Quan" or "Taichi" or "Taichi quan" or ‘baduanjin’ or "ba duan jin" OR "eight section brocades" OR "eight trigrams boxing" OR "eight-treasured exercises" OR "eight pieces of brocade" OR "eight brocade section" OR "eight-section brocade" or Yoga or "Five-animal exercises" or "Five Animal Exercise" or "Five animal Frolics" or "movements of five animals" or "Five mimic-animal boxing" or "Wuqinxi" or "wu qin xi" or "Liuzijue" or "liu zi jue" or "six-character formula" or "Six Healing Sounds" or Yijinjing or "yi jin jing"):ti,ab,kw  #5 ("aerobic exercise" or "aerobic training" or "Flexibility training" or "multidisciplinary exercise program" or "Nordic Walking" or "Physiotherapy" or "pilates" or "power training" or "treadmill training" or "walking"):ti,ab,kw  #6 ("High Intensity Interval Training" or "High-Intensity Interval Trainings" or "IntervalTraining,High-Intensity" or "Training,High-Intensity Interval" or "Trainings, High-Intensity Interval" or "High-Intensity Intermittent Exercise" or "Exercise, High-Intensity Intermittent" or "Exercises, High-Intensity Intermittent" or "High-Intensity Intermittent Exercises" or "SprintIntervalTraining" or "SprintIntervalTrainings"):ti,ab,kw  #7 ("respiratory muscle" or "Respiratory Muscle Training" OR "Expiratory muscle training" OR "Training, Respiratory Muscle"):ti,ab,kw  #8 ("water-based" OR "aquatic" OR "Hydrotherapies" OR "Whirlpool Baths" OR "Bath, Whirlpool"):ti,ab,kw  #9 (Exercise or Exercises or "Exercise, Physical" or "Exercises, Physical" or "Physical Exercise" or "Physical Exercises" or "Physical Activity" or "Activities, Physical" or "Activity, Physical" or "Physical Activities" or "Exercise, Aerobic" or "Aerobic Exercise" or "Aerobic Exercises" or "Exercises, Aerobic" or "Exercise, Isometric" or "Exercises, Isometric" or "Isometric Exercises" or "Isometric Exercise" or "Acute Exercise" or "Acute Exercises" or "Exercise, Acute" or "Exercises, Acute" or "Exercise Training" or "Exercise Trainings" or "Training, Exercise" or "Trainings, Exercise"):ti,ab,kw  #10 ('heart function tests' OR 'function test, heart' OR 'function tests, heart' OR 'heart function test' OR 'test, heart function' OR 'tests, heart function' OR 'cardiac function tests' OR 'cardiac function test' OR 'function test, cardiac' OR 'function tests, cardiac' OR 'test, cardiac function' OR 'tests, cardiac function' OR 'peak vo2' OR '6mwt' OR 'lvef'):ti,ab,kw  #11 ("controlled trial, randomized" OR "controlled trial, randomized" OR "randomised controlled study" OR "randomised controlled study" OR "randomised controlled trial" OR "randomised controlled trial" OR "randomized controlled study" OR "randomized controlled study" OR "randomized controlled trial" OR "randomized controlled trial" OR "trial, randomized controlled" OR "trial, randomized controlled"):ti,ab,kw  #12 #1 or #2  #13 #3 or #4 or #5 or #6 or #7 or #8 or #9  #14 #10 and #11 and #12 and #13 |
| Embase | #1 'myocardial infarction':ti,ab,kw  #2 'infarct, myocardial':ti,ab,kw OR 'cardiovascular strokes':ti,ab,kw OR 'infarction, myocardial':ti,ab,kw OR 'myocardial infarcts':ti,ab,kw OR 'strokes, cardiovascular':ti,ab,kw OR 'heart attack':ti,ab,kw OR 'stroke, cardiovascular':ti,ab,kw OR 'heart attacks':ti,ab,kw OR 'infarcts, myocardial':ti,ab,kw OR 'infarctions, myocardial':ti,ab,kw OR 'myocardial infarctions':ti,ab,kw OR 'cardiovascular stroke':ti,ab,kw OR 'myocardial infarct':ti,ab,kw  #3 'resistance training':ti,ab,kw OR 'training, resistance':ti,ab,kw OR 'strength training':ti,ab,kw OR 'training, strength':ti,ab,kw OR 'weight-lifting strengthening program':ti,ab,kw OR 'strengthening program,weight-lifting':ti,ab,kw OR 'strengthening programs,weight-lifting':ti,ab,kw OR 'weight liftingstrengtheningprogram':ti,ab,kw OR 'weight lifting strengthening programs':ti,ab,kw OR 'weight-lifting exercise program':ti,ab,kw OR 'exercise program,weight-lifting':ti,ab,kw OR 'exercise programs, weight-lifting':ti,ab,kw OR 'weight liftingexerciseprogram':ti,ab,kw OR 'weight-lifting exercise programs':ti,ab,kw OR 'weight-bearing strengthening program':ti,ab,kw OR 'strengthening program, weight-bearing':ti,ab,kw OR 'strengthening programs, weight-bearing':ti,ab,kw OR 'weight bearing strengthening program':ti,ab,kw OR 'weight-bearing strengthening programs':ti,ab,kw OR 'weight-bearing exercise program':ti,ab,kw  #4 'aerobicexercise':ti,ab,kw OR 'aerobictraining':ti,ab,kw OR 'flexibilitytraining':ti,ab,kw OR 'multidisciplinary exercise program':ti,ab,kw OR 'nordic walking':ti,ab,kw OR 'physiotherapy':ti,ab,kw OR 'pilates':ti,ab,kw OR 'power training':ti,ab,kw OR 'treadmill training':ti,ab,kw OR 'walking':ti,ab,kw  #5 'high intensity interval training':ti,ab,kw OR 'high-intensity interval trainings':ti,ab,kw OR 'intervaltraining,high-intensity':ti,ab,kw OR 'training,high-intensity interval':ti,ab,kw OR 'trainings, high-intensity interval':ti,ab,kw OR 'high-intensity intermittent exercise':ti,ab,kw OR 'exercise, high-intensity intermittent':ti,ab,kw OR 'exercises, high-intensity intermittent':ti,ab,kw OR 'high-intensity intermittent exercises':ti,ab,kw OR 'sprintintervaltraining':ti,ab,kw OR 'sprintintervaltrainings':ti,ab,kw  #6 'mind-body exercises':ti,ab,kw OR 'tai chi':ti,ab,kw OR 'tai ji':ti,ab,kw OR 'tai chi exercise':ti,ab,kw OR 'tai ji chuan':ti,ab,kw OR 'tai ji quan':ti,ab,kw OR 'tai chi chuan':ti,ab,kw OR 'tai chi quan':ti,ab,kw OR 'taichi':ti,ab,kw OR 'taichi quan':ti,ab,kw OR 'baduanjin':ti,ab,kw OR 'ba duan jin':ti,ab,kw OR 'eight section brocades':ti,ab,kw OR 'eight trigrams boxing':ti,ab,kw OR 'eight-treasured exercises':ti,ab,kw OR 'eight pieces of brocade':ti,ab,kw OR 'eight brocade section':ti,ab,kw OR 'eight-section brocade':ti,ab,kw OR yoga:ti,ab,kw OR 'five-animal exercises':ti,ab,kw OR 'five animal exercise':ti,ab,kw OR 'five animal frolics':ti,ab,kw OR 'movements of five animals':ti,ab,kw OR 'five mimic-animal boxing':ti,ab,kw OR 'wuqinxi':ti,ab,kw OR 'wu qin xi':ti,ab,kw OR 'liuzijue':ti,ab,kw OR 'liu zi jue':ti,ab,kw OR 'six-character formula':ti,ab,kw OR 'six healing sounds':ti,ab,kw OR yijinjing:ti,ab,kw OR 'yi jin jing':ti,ab,kw  #7 'respiratory muscle':ti,ab,kw OR 'respiratory muscle training':ti,ab,kw OR 'expiratory muscle training':ti,ab,kw OR 'training, respiratory muscle':ti,ab,kw  #8 'water-based':ti,ab,kw OR 'aquatic':ti,ab,kw OR 'hydrotherapies':ti,ab,kw OR 'whirlpool baths':ti,ab,kw OR 'bath, whirlpool':ti,ab,kw  #9 exercise:ti,ab,kw OR exercises:ti,ab,kw OR 'exercise, physical':ti,ab,kw OR 'exercises, physical':ti,ab,kw OR 'physical exercise':ti,ab,kw OR 'physical exercises':ti,ab,kw OR 'physical activity':ti,ab,kw OR 'activities, physical':ti,ab,kw OR 'activity, physical':ti,ab,kw OR 'physical activities':ti,ab,kw OR 'exercise, aerobic':ti,ab,kw OR 'aerobic exercise':ti,ab,kw OR 'aerobic exercises':ti,ab,kw OR 'exercises, aerobic':ti,ab,kw OR 'exercise, isometric':ti,ab,kw OR 'exercises, isometric':ti,ab,kw OR 'isometric exercises':ti,ab,kw OR 'isometric exercise':ti,ab,kw OR 'acute exercise':ti,ab,kw OR 'acute exercises':ti,ab,kw OR 'exercise, acute':ti,ab,kw OR 'exercises, acute':ti,ab,kw OR 'exercise training':ti,ab,kw OR 'exercise trainings':ti,ab,kw OR 'training, exercise':ti,ab,kw OR 'trainings, exercise':ti,ab,kw  #10 'heart function tests':ti,ab,kw OR 'function test, heart':ti,ab,kw OR 'function tests, heart':ti,ab,kw OR 'heart function test':ti,ab,kw OR 'test, heart function':ti,ab,kw OR 'tests, heart function':ti,ab,kw OR 'cardiac function tests':ti,ab,kw OR 'cardiac function test':ti,ab,kw OR 'function test, cardiac':ti,ab,kw OR 'function tests, cardiac':ti,ab,kw OR 'test, cardiac function':ti,ab,kw OR 'tests, cardiac function':ti,ab,kw OR 'peak vo2':ti,ab,kw OR '6mwt':ti,ab,kw OR 'lvef':ti,ab,kw  #11 ('controlled trial, randomized'/exp OR 'controlled trial, randomized' OR 'randomised controlled study'/exp OR 'randomised controlled study' OR 'randomised controlled trial'/exp OR 'randomised controlled trial' OR 'randomized controlled study'/exp OR 'randomized controlled study' OR 'randomized controlled trial'/exp OR 'randomized controlled trial' OR 'trial, randomized controlled'/exp OR 'trial, randomized controlled') AND [randomized controlled trial]/lim  #12 #1 or #2  #13 #3 OR #4 OR #5 OR #6 OR #7 OR #8 OR #9  #14 #10 AND #11 AND #12 AND #13 |
| Web of Science | #1 TS=(Myocardial Infarction) and Preprint Citation Index (Exclude – Database)  #2 TS=("Infarct, Myocardial" OR "Cardiovascular Strokes" OR "Infarction, Myocardial" OR "Myocardial Infarcts" OR "Strokes, Cardiovascular" OR " Heart Attack" OR "Stroke, Cardiovascular" OR "Heart Attacks" OR "Infarcts, Myocardial" OR "Infarctions, Myocardial" OR "Myocardial Infarctions" OR "Cardiovascular Stroke" OR "Myocardial Infarct") and Preprint Citation Index (Exclude – Database)  #3 TS=("Resistance training" or "Training, Resistance" or "Strength Training" or "Training, Strength" or "Weight-Lifting Strengthening Program" or "Strengthening Program,Weight-Lifting" or "Strengthening Programs,Weight-Lifting" or "Weight LiftingStrengtheningProgram" or "Weight Lifting Strengthening Programs" or "Weight-Lifting Exercise Program" or "Exercise Program,Weight-Lifting" or "Exercise Programs, Weight-Lifting" or "Weight LiftingExerciseProgram" or "Weight-Lifting Exercise Programs" or "Weight-Bearing Strengthening Program" or "Strengthening Program, Weight-Bearing" or "Strengthening Programs, Weight-Bearing" or "Weight Bearing Strengthening Program" or "Weight-Bearing Strengthening Programs" or "Weight-Bearing Exercise Program") and Preprint Citation Index (Exclude – Database)  #4 TS=("mind-body exercises" or "Tai Chi" or "Tai Ji" or "Tai Chi Exercise" or "Tai Ji Chuan" or "Tai Ji Quan" or "Tai Chi Chuan" or "Tai Chi Quan" or "Taichi" or "Taichi quan" or ‘baduanjin’ or "ba duan jin" OR "eight section brocades" OR "eight trigrams boxing" OR "eight-treasured exercises" OR "eight pieces of brocade" OR "eight brocade section" OR "eight-section brocade" or Yoga or "Five-animal exercises" or "Five Animal Exercise" or "Five animal Frolics" or "movements of five animals" or "Five mimic-animal boxing" or "Wuqinxi" or "wu qin xi" or "Liuzijue" or "liu zi jue" or "six-character formula" or "Six Healing Sounds" or Yijinjing or "yi jin jing") and Preprint Citation Index (Exclude – Database)  #5 TS=("aerobicexercise" or "aerobictraining" or "Flexibilitytraining" or "multidisciplinary exercise program" or "Nordic Walking" or "Physiotherapy" or "pilates" or "power training" or "treadmill training" or "walking") and Preprint Citation Index (Exclude – Database)  #6 TS=("High Intensity Interval Training" or "High-Intensity Interval Trainings" or "IntervalTraining,High-Intensity" or "Training,High-Intensity Interval" or "Trainings, High-Intensity Interval" or "High-Intensity Intermittent Exercise" or "Exercise, High-Intensity Intermittent" or "Exercises, High-Intensity Intermittent" or "High-Intensity Intermittent Exercises" or "SprintIntervalTraining" or "SprintIntervalTrainings") and Preprint Citation Index (Exclude – Database)  #7 TS=("respiratory muscle" or "Respiratory Muscle Training" OR "Expiratory muscle training" OR "Training, Respiratory Muscle") and Preprint Citation Index (Exclude – Database)  #8 TS=("water-based" OR "aquatic" OR "Hydrotherapies" OR "Whirlpool Baths" OR "Bath, Whirlpool") and Preprint Citation Index (Exclude – Database)  #9 TS=(Exercise or Exercises or "Exercise, Physical" or "Exercises, Physical" or "Physical Exercise" or "Physical Exercises" or "Physical Activity" or "Activities, Physical" or "Activity, Physical" or "Physical Activities" or "Exercise, Aerobic" or "Aerobic Exercise" or "Aerobic Exercises" or "Exercises, Aerobic" or "Exercise, Isometric" or "Exercises, Isometric" or "Isometric Exercises" or "Isometric Exercise" or "Acute Exercise" or "Acute Exercises" or "Exercise, Acute" or "Exercises, Acute" or "Exercise Training" or "Exercise Trainings" or "Training, Exercise" or "Trainings, Exercise") and Preprint Citation Index (Exclude – Database)  #10 TS=('heart function tests' OR 'function test, heart' OR 'function tests, heart' OR 'heart function test' OR 'test, heart function' OR 'tests, heart function' OR 'cardiac function tests' OR 'cardiac function test' OR 'function test, cardiac' OR 'function tests, cardiac' OR 'test, cardiac function' OR 'tests, cardiac function' OR 'peak vo2' OR '6mwt' OR 'lvef') and Preprint Citation Index (Exclude – Database)  #11 TS=("controlled trial, randomized" OR "controlled trial, randomized" OR "randomised controlled study" OR "randomised controlled study" OR "randomised controlled trial" OR "randomised controlled trial" OR "randomized controlled study" OR "randomized controlled study" OR "randomized controlled trial" OR "randomized controlled trial" OR "trial, randomized controlled" OR "trial, randomized controlled"') and Preprint Citation Index (Exclude – Database)  #12 #1 or #2  #13 #3 or #4 or #5 or #6 or #7 or #8 or #9  #14 #10 and #11 and #12 and #13 |
| Pubmed | #1 Myocardial Infarction[Title/Abstract]  #2 "Infarct, Myocardial"[Title/Abstract] OR "Cardiovascular Strokes"[Title/Abstract] OR "Infarction, Myocardial"[Title/Abstract] OR "Myocardial Infarcts"[Title/Abstract] OR "Strokes, Cardiovascular"[Title/Abstract] OR " Heart Attack"[Title/Abstract] OR "Stroke, Cardiovascular"[Title/Abstract] OR "Heart Attacks"[Title/Abstract] OR "Infarcts, Myocardial"[Title/Abstract] OR "Infarctions, Myocardial"[Title/Abstract] OR "Myocardial Infarctions"[Title/Abstract] OR "Cardiovascular Stroke"[Title/Abstract] OR "Myocardial Infarct"[Title/Abstract]  #3 "Resistance training"[Title/Abstract] OR "Training, Resistance"[Title/Abstract] OR "Strength Training"[Title/Abstract] OR "Training, Strength"[Title/Abstract] OR "Weight-Lifting Strengthening Program"[Title/Abstract] OR "Strengthening Program,Weight-Lifting"[Title/Abstract] OR "Strengthening Programs,Weight-Lifting"[Title/Abstract] OR "Weight LiftingStrengtheningProgram"[Title/Abstract] OR "Weight Lifting Strengthening Programs"[Title/Abstract] OR "Weight-Lifting Exercise Program"[Title/Abstract] OR "Exercise Program,Weight-Lifting"[Title/Abstract] OR "Exercise Programs, Weight-Lifting"[Title/Abstract] OR "Weight LiftingExerciseProgram"[Title/Abstract] OR "Weight-Lifting Exercise Programs"[Title/Abstract] OR "Weight-Bearing Strengthening Program"[Title/Abstract] OR "Strengthening Program, Weight-Bearing"[Title/Abstract] OR "Strengthening Programs, Weight-Bearing"[Title/Abstract] OR "Weight Bearing Strengthening Program"[Title/Abstract] OR "Weight-Bearing Strengthening Programs"[Title/Abstract] OR "Weight-Bearing Exercise Program"[Title/Abstract]  #4 "mind-body exercises"[Title/Abstract] OR "Tai Chi"[Title/Abstract] OR "Tai Ji"[Title/Abstract] OR "Tai Chi Exercise"[Title/Abstract] OR "Tai Ji Chuan"[Title/Abstract] OR "Tai Ji Quan"[Title/Abstract] OR "Tai Chi Chuan"[Title/Abstract] OR "Tai Chi Quan"[Title/Abstract] OR "Taichi"[Title/Abstract] OR "Taichi quan"[Title/Abstract] OR ‘baduanjin’[Title/Abstract] OR ‘ba duan jin’[Title/Abstract] OR ‘eight section brocades’[Title/Abstract] OR ‘eight trigrams boxing’[Title/Abstract] OR ‘eight-treasured exercises’[Title/Abstract] OR ‘eight pieces of brocade’[Title/Abstract] OR ‘eight brocade section’[Title/Abstract] OR ‘eight-section brocade’[Title/Abstract] OR "Five-animal exercises"[Title/Abstract] OR "Five Animal Exercise"[Title/Abstract] OR "Five animal Frolics"[Title/Abstract] OR "movements of five animals"[Title/Abstract] OR "Five mimic-animal boxing"[Title/Abstract] OR "Wuqinxi"[Title/Abstract] OR "wu qin xi"[Title/Abstract] OR "Liuzijue"[Title/Abstract] OR "liu zi jue"[Title/Abstract] OR "six-character formula"[Title/Abstract] OR "Six Healing Sounds"[Title/Abstract] OR Yijinjing[Title/Abstract] OR "yi jin jing"[Title/Abstract]  #5 "aerobicexercise" or "aerobictraining" or "Flexibilitytraining" or "multidisciplinary exercise program" or "Nordic Walking" or "Physiotherapy" or "pilates" or "power training" or "treadmill training" or "walking"  #6 "High Intensity Interval Training"[Title/Abstract] OR "High-Intensity Interval Trainings"[Title/Abstract] OR "IntervalTraining,High-Intensity"[Title/Abstract] OR "Training,High-Intensity Interval"[Title/Abstract] OR "Trainings, High-Intensity Interval"[Title/Abstract] OR "High-Intensity Intermittent Exercise"[Title/Abstract] OR "Exercise, High-Intensity Intermittent"[Title/Abstract] OR "Exercises, High-Intensity Intermittent"[Title/Abstract] OR "High-Intensity Intermittent Exercises"[Title/Abstract] OR "SprintIntervalTraining"[Title/Abstract] OR "SprintIntervalTrainings"[Title/Abstract]  #7 **"respiratory muscle"[Title/Abstract] OR "Respiratory Muscle Training"[Title/Abstract] OR "Expiratory muscle training"[Title/Abstract] OR "Training, Respiratory Muscle"[Title/Abstract]**  #8 "water-based"[Title/Abstract] OR "aquatic"[Title/Abstract] OR "Hydrotherapies"[Title/Abstract] OR "Whirlpool Baths"[Title/Abstract] OR "Bath, Whirlpool"[Title/Abstract]  #9 Exercise[Title/Abstract] OR Exercises[Title/Abstract] OR "Exercise, Physical"[Title/Abstract] OR "Exercises, Physical"[Title/Abstract] OR "Physical Exercise"[Title/Abstract] OR "Physical Exercises"[Title/Abstract] OR "Physical Activity"[Title/Abstract] OR "Activities, Physical"[Title/Abstract] OR "Activity, Physical"[Title/Abstract] OR "Physical Activities"[Title/Abstract] OR "Exercise, Aerobic"[Title/Abstract] OR "Aerobic Exercise"[Title/Abstract] OR "Aerobic Exercises"[Title/Abstract] OR "Exercises, Aerobic"[Title/Abstract] OR "Exercise, Isometric"[Title/Abstract] OR "Exercises, Isometric"[Title/Abstract] OR "Isometric Exercises"[Title/Abstract] OR "Isometric Exercise"[Title/Abstract] OR "Acute Exercise"[Title/Abstract] OR "Acute Exercises"[Title/Abstract] OR "Exercise, Acute"[Title/Abstract] OR "Exercises, Acute"[Title/Abstract] OR "Exercise Training"[Title/Abstract] OR "Exercise Trainings"[Title/Abstract] OR "Training, Exercise"[Title/Abstract] OR "Trainings, Exercise"[Title/Abstract]  #10 "Heart Function Tests"[Title/Abstract] OR "Function Test, Heart"[Title/Abstract] OR "Function Tests, Heart"[Title/Abstract] OR "Heart Function Test"[Title/Abstract] OR "Test, Heart Function"[Title/Abstract] OR "Tests, Heart Function"[Title/Abstract] OR "Cardiac Function Tests"[Title/Abstract] OR "Cardiac Function Test"[Title/Abstract] OR "Function Test, Cardiac"[Title/Abstract] OR "Function Tests, Cardiac"[Title/Abstract] OR "Test, Cardiac Function"[Title/Abstract] OR "Tests, Cardiac Function"[Title/Abstract] OR "peak VO2"[Title/Abstract] OR "6MWT"[Title/Abstract] OR "LVEF"[Title/Abstract]  #11 'randomized controlled trial' OR 'controlled trial, randomized' OR 'randomised controlled study' OR 'randomised controlled trial' OR 'randomized controlled study' OR 'randomized controlled trial' OR 'trial, randomized controlled'  #12 #1 or #2  #13 #3 or #4 or #5 or #6 or #7 or #8 or #9  #14 #10 and #11 and #12 and #13 |
| Chinese Biomedical Database | ( "心肌梗死患者"[中文标题:智能] OR "心梗患者"[中文标题:智能]) AND( "抗阻训练"[中文标题:智能] OR "身心运动"[中文标题:智能] OR "有氧运动"[中文标题:智能] OR "高强度间歇"[中文标题:智能] OR "呼吸肌训练"[中文标题:智能] OR "水中运动"[中文标题:智能] OR "运动"[中文标题:智能] OR "锻炼"[中文标题:智能] OR "太极"[中文标题:智能] OR "八段锦"[中文标题:智能] OR "易筋经"[中文标题:智能] OR "瑜伽"[中文标题:智能] OR "舞蹈"[中文标题:智能] OR "普拉提"[中文标题:智能] OR "五禽戏"[中文标题:智能] OR "六字诀"[中文标题:智能]) AND "心功能"[中文标题:智能] |
| China National Knowledge Infrastructure | 篇关摘:(心肌梗死患者 + 心梗患者) And 篇关摘:(抗阻训练 + 身心运动 + 太极 + 瑜伽 + 八段锦 + 气功 + 普拉提 + 舞蹈 + 易筋经 + 六字诀 + 有氧运动 + 高强度间歇 + 呼吸肌训练 + 水中运动 + 运动 + 锻炼) And 篇关摘:( 心功能 + "峰值摄氧量" + "6分钟步行测试" + "左心室射血分数" + "peak VO2" + "6MWT" + "LVEF" ） |
| Wanfang Database | 题名或关键词:(心肌梗死患者 or 心梗患者) and 题名或关键词:(抗阻训练 or 身心运动 or 太极 or 瑜伽 or 八段锦 or 气功 or 普拉提 or 舞蹈 or 易筋经 or 六字诀 or 有氧运动 or 高强度间歇 or 呼吸肌训练 or 水中运动 or 运动 or 锻炼) and 题名或关键词:(心功能 + "峰值摄氧量" or "6分钟步行测试" or "左心室射血分数" or "peak VO2" or "6MWT" or "LVEF" ) |
| Chinese Science and Technology Periodical Database | （题名或关键词：心肌梗死患者+心梗患者）AND（题名或关键词：抗阻训练+身心运动+有氧运动+高强度间歇+呼吸肌训练+水中运动+运动+锻炼+太极+八段锦+易筋经+瑜伽+舞蹈+普拉提+五禽戏+六字诀）+ （题名或关键词：心功能 + "峰值摄氧量" + "6分钟步行测试" + "左心室射血分数" + "peak VO2" + "6MWT" + "LVEF"） |

Appendix 3. Definition of exercise type and control group

| Exercise type name and abbreviation | Definition |
| --- | --- |
| Aerobic exercises（AE） | AE refers to the use of oxygen in the process of muscle energy production. It can be continuous or intermittent or at different intensities. Walking, |
| Resistance exercises（RE) | RE mainly refers to forms of exercise that strengthen skeletal muscle strength, explosiveness, endurance and volume through resistance training. |
| Mind-body exercises（MBE) | MBE is a type of exercise that emphasises the interconnection and mutual influence of the body and mind, and aims to promote physical and mental health, improve psychological state, and achieve self-awareness and growth through specific physical activities. Tai chi, yoga, etc. are the mainstream forms. |
| High-intensity interval training（HIIT） | HIIT is a training method that combines high-intensity exercise with low-intensity exercise or short breaks. |
| Multicomponent exercise training（MCET) | MCET is a comprehensive form of exercise that organically combines multiple different types of exercise elements, aiming to comprehensively improve various body functions and promote overall health. In this study, MCET is defined as an intervention that includes two or more of the above exercise forms. |
| Control group | The control group was administered conventional treatments, including regular medication, typical care, and verbal instruction. |

Appendix 4. Detailed characteristics of the included studies

| Author/Year | Country | Sample size | Gender  （Male/Female） | Age  (X̅±s) | Intervention | Brief description | Length  (week) | Frequency  (times/week) | Duration/  Per session（min） | Outcomes | Supervision status of intervention measures | Diagnostic criteria for MI | Types of MI | Imaging methods | Background treatment | Adherence rates and adverse events |
| --- | --- | --- | --- | --- | --- | --- | --- | --- | --- | --- | --- | --- | --- | --- | --- | --- |
| Ding et al. 2024 (1) | China | 45 | 33/12 | 64.83±4.63 | RE | ①Lateral flexion: Place one hand on the temple of the head, bend the neck laterally to bring the ear as close as possible to the shoulder, while the hand resists the force of the lateral flexion and strives to maintain this position, alternating sides.② Rotation: Place one hand on the temple of the head, turn the head towards the shoulder, while the hand resists the force of rotation and strives to maintain this position, alternating sides.③ Flexion and extension: Place both hands on the forehead, bending the head forward while the hands resist the force of flexion and strive to maintain this position; then place both hands on the occipital region, extending the head backward while the hands resist the force of extension and strive to maintain this position.④ Backward tilt: The patient lies supine with the head extending beyond the edge of the bed. The caregiver supports the patient’s head with both hands on the occipital region while the patient exerts force to tilt the head backward, and the caregiver resists this backward motion with both hands.Each of the movements from ① to ④ is performed for 15 seconds, with 20 repetitions constituting one set. Each exercise is performed as one set daily, commencing on the seventh day post-surgery.⑤ Walking: During walking, patients may be advised to lift their calves appropriately, use bottled water for added weight, and employ resistance bands to increase resistance. For younger patients, resistance bands may be chosen to increase resistance, provided their physical condition allows. | 12 | 7 | 20 | 6MWT/LVEF/LVESD/LVEDD | Under supervision | European Society of Cardiology Guidelines | AMI | Echocardiography | PCI | NR |
|  | China | 45 | 32/13 | 64.17±4.51 | Control (B) | Perform corresponding passive limb exercises, primarily focusing on elbow flexion and arm extension, with each training session lasting approximately 5 to 10 minutes.Limit the number of passive training sessions to 2 to 3 times per day, and perform appropriate massage on the joint areas after each session.Advise the patient to change positions every 2 hours. | 12 | 7 |  |  |  |  |  |  |  |  |
| Du et al. 2023 (2) | China | 40 | 22/18 | 64.37±3.22 | RE | Resistance Training. Before the training begins, an assessment of the patient's one-repetition maximum (1RM) will be conducted to determine an appropriate training intensity based on the evaluation results. For the first 1 to 6 weeks post-discharge, patients are advised to train at 50% of their maximum load; after 7 weeks post-discharge, the maximum load should be increased to 60%. The primary focus of training will be on the abdominal muscle groups, upper and lower limb muscle groups, and lumbar muscle groups. Training modalities may include the use of dumbbells, sandbags, resistance bands, and other equipment. | 24 | 7 | 25 | LVEF/6WMT/LVESD/LVEDD | Unclear | Guidelines for the Diagnosis and Treatment of Acute Myocardial Infarction | AMI | Echocardiography | PCI | NR |
|  | China | 40 | 23/17 | 65.23±3.11 | MCET | Day 1: Focus on passive activities for major muscle groups and joints while in bed, with a training duration of 5 to 10 minutes per session.Day 2: Emphasize active movements of the contralateral limbs while in bed, with a training duration of 5 to 10 minutes per session.Days 3 to 5: Concentrate on active movements at the bedside, guiding the patient to walk slowly within the ward for distances of 25 to 70 meters, with a training duration of 10 to 20 minutes per session.Days 6 to 7: Guide the patient to walk slowly in the ward for distances of 50 to 100 meters, with a training duration of 15 to 25 minutes per session.Days 8 to 14: Instruct the patient to walk slowly within the ward for distances of 100 to 300 meters or to ascend and descend 8 steps, with a training duration of 25 to 50 minutes per session.Days 15 to 6 Months: Continue to guide the patient to walk slowly for distances of 100 to 300 meters or to ascend and descend 8 steps, with a training duration of 25 to 50 minutes per session. | 24 | 7 | 25-50 |  |  |  |  |  |  |  |
| Jia 2018 (3)^3^ | China | 58 | 31/27 | 53.64±6.18 | RE | Resistance training exercises will typically involve muscle groups of the upper and lower limbs, as well as the lumbar, back, and abdominal regions.A total of six movements will be included: quadriceps stretches, calf raises, shoulder presses, chest presses, triceps stretches, and bicep curls. | 12 | 7 | 25 | LVEF/6WMT/LVESD/LVEDD | Under supervision | Meet the diagnostic criteria for acute myocardial infarction established by the WHO. | MI | Echocardiography | PCI;  taking statin lipid-lowering drugs, antiplatelet drugs, angiotensin-converting enzyme inhibitors, etc. after surgery | The total incidence of cardiovascular events in the observation group was significantly lower than that in the control group (P<0.05) |
|  | China | 58 | 32/26 | 63.7±6.21 | Control (B) | The control group will receive routine continued care after discharge, engaging in daily activities according to their individual needs.Outpatient follow-up will occur once a month to monitor their physical condition and medication usage. | 12 |  |  |  |  |  |  |  |  |  |
| Yang et al. 2022 (4) | China | 57 | 37/20 | 56.03±5.67 | RE | The warm-up exercise duration is 10 minutes.Resistance training will include shoulder joint adduction, abduction, knee extension, and flexion exercises, with each training session lasting 40 minutes.Following resistance training, appropriate stretching activities will be conducted for a duration of 10 minutes. | 12 | 3 | 60 | LVEF/6MWT/LVESD/LVEDD | Under supervision | Guidelines for the Diagnosis and Treatment of Acute ST-Segment Elevation Myocardial Infarction | AMI | Echocardiography | PCI;  routine medications (specific drugs not specified) | There was no significant difference in adverse cardiovascular events (angina pectoris, arrhythmia) between the observation group and the control group. |
|  | China | 56 | 35/21 | 55.50 ±5.22 | AE | Assist the patient in standing at the bedside for 5 minutes each time, with a goal of standing twice a day.Under the supervision of family members, the patient will walk 30 to 50 meters within the ward.Instruct the patient to sit in a chair for 2 to 3 hours. Under the supervision of others, the patient will walk slowly in the corridor for 80 to 100 meters and learn to measure their own pulse.Allow the patient to sit in a chair for 3 to 4 hours. Under supervision, the patient will walk 300 to 350 meters and engage in activities for 30 minutes.Continue the activities from the previous day, increasing walking distance to 400 to 500 meters and climbing two flights of stairs as tolerated. | 12 | 7 | Unclear |  |  |  |  |  |  |  |
| Wu et al. 2017 (5) | China | 30 | 26/4 | 60~80 | RE | Resistance Band Selection: The resistance training modality will utilize resistance bands for exercises.Resistance Band Exercise Protocol: The exercise frequency will be 3 times per week, consisting of three main steps:Warm-up Exercise: 10 minutes.Resistance Training: 30 to 40 minutes. This includes resistance band exercises targeting shoulder joint adduction, abduction, and knee flexion and extension, comprising a total of four movements. Each movement will be performed for 8 to 10 minutes.Cool-down Exercise: 10 minutes. The intensity of the exercise will gradually decrease, incorporating appropriate stretching exercises, which may include some of the movements from the warm-up.Timing for Training: Training will commence 1 month post-percutaneous coronary intervention (PCI) surgery, provided that the electrocardiogram is normal, the patient experiences no chest discomfort, and blood pressure remains stable. | 24 | 3 | 50-60 | 6MWT | Under supervision | Guidelines for the Diagnosis and Treatment of Acute Myocardial Infarction | AMI | Echocardiography | PCI;  taking atorvastatin, antiplatelet drugs, beta-blockers, angiotensin-converting enzyme inhibitors, etc. | The average daily frequency and duration of chest discomfort in the observation group were significantly lower than those in the control group. |
|  | China | 34 | 27/7 | 60~80 | Control (B) | Medication Treatment: Following percutaneous coronary intervention (PCI) and stent placement, patients will be prescribed atorvastatin for long-term use. All patients will routinely receive antiplatelet medications, β-blockers, and angiotensin-converting enzyme (ACE) inhibitors. Postoperatively, regular monitoring of lipid profiles, blood glucose levels, and liver and kidney function will be conducted.Post-PCI Health Management Model: A WeChat group will be established for patients and their families. This platform will be utilized to provide online guidance and address any questions or concerns. | 24 |  |  |  |  |  |  |  |  |  |
| Zhu et al. 2023 (6) | China | 35 | 18/17 | 60.22±5.22 | MBE | Stretching of the Feet: Place both feet comfortably while crossing the hands to support.Rotational Movements: Perform left and right rotational movements.Breath Control with Hand Rubbing: Hold the breath while rubbing the hands to generate warmth, then rub the back and lower abdomen. Visualize a sensation of heat at the navel as you exhale completely.Dragon Stirring Water: Imagine stirring water with the dragon. Practice swishing the saliva thirty-six times, filling the mouth evenly with saliva, and swallowing in three portions, allowing the energy to flow naturally.Gentle Shaking: Slightly sway the body as if shaking the heavenly pillar. Lower the head and twist the neck to look left and right, allowing the shoulders to sway correspondingly, repeating this movement 24 times on each side.Meditative Sitting: Close the eyes and focus inwardly, engaging in deep thought. Tap the teeth thirty-six times, while holding the hands in a protective position, and perform left and right movements to sound the heavenly drum, completing this 24 times. | 4 | Unclear | Unclear | LVEF/6MWT | Under supervision | Meet the confirmed diagnostic criteria for acute myocardial infarction. | AMI | Echocardiography | PCI | NR |
|  | China | 35 | 19/16 | 60.11±4.89 | Control (B) | Standard Cardiac Rehabilitation Care Plan:The care plan will primarily include nutritional guidance, psychological support, medication management, and smoking cessation assistance. | 4 |  |  |  |  |  |  |  |  |  |
| Yu et al. 2022 (7) | China | 53 | 45/8 | 60.4±11.37 | MBE | Observation Group Protocol:In addition to standard rehabilitation, the observation group will commence guided "Seated Ba Duan Jin" rehabilitation therapy 24 hours after emergency PCI surgery. If the patient's condition does not permit, the start of this therapy may be appropriately delayed until 48 hours post-surgery. Training sessions will occur daily from 8:30 AM to 4:30 PM, with each set of exercises lasting approximately 12 minutes, performed twice with a 5-minute rest in between, totaling around half an hour. | 24 | 7 | 30 | LVEF/6MWT/LVEDD/LVESD | Under supervision | Meet the criteria in the "Universal Definition of Myocardial Infarction" | AMI | Echocardiography | PCI | NR |
|  | China | 53 | 44/9 | 61.13±11.06 | Control (B) | Postoperative Standard Rehabilitation Protocol:Rehabilitation will commence 24 hours after emergency PCI surgery, beginning with the patient performing independent turns in bed, sitting up, and transitioning to a chair beside the bed. At 48 hours post-surgery, the patient may be allowed to walk short distances (approximately 2 to 5 meters) and use the restroom. At 72 hours, the patient can walk appropriately in the hospital corridor, aiming for 2 to 3 sessions daily, each lasting 5 to 10 minutes, tailored to the patient's individual condition. After 96 hours, the patient may increase their activity by either ascending one flight of stairs or increasing the frequency of walking in the ward to 3 to 4 times per session. | 24 |  |  |  |  |  |  |  |  |  |
| Gui 2022 (8) | China | 55 | 35/20 | 57.50±5.59 | MBE | Ba Duan Jin Exercise Descriptions:Hands Supporting the Sky: Raise both hands overhead, symbolizing the regulation of the triple warmer (San Jiao).Drawing the Bow: Mimic the action of drawing a bow to strengthen the body, akin to aiming an arrow.Regulating the Spleen and Stomach: Perform movements that focus on single-arm lifts to enhance digestion and strengthen the core.Looking Back at Past Strains: Turn the head back to release tension and address any physical fatigue or discomfort.Shaking Head and Tail: Move the head side to side to eliminate excess heart heat and promote relaxation. Reaching for the Feet: Bend forward to stretch the lower back and strengthen the kidneys.Fist Clenching with Intensity: Form tight fists and focus on generating strength and determination.Seven Bounces Backward: Gently bounce backward to dispel ailments and invigorate overall health. | Unclear | 7 | 30 | LVEF/LVEDD/LVESD | Under supervision | Chinese Guidelines for Percutaneous Coronary Intervention (2016) | MI | Echocardiography | PCI | NR |
|  | China | 55 | 34/21 | 57.68±5.64 | Control (B) | During the patient's hospital stay, informational brochures regarding the disease and PCI treatment will be routinely provided. Patients will be encouraged to read this material independently to enhance their understanding of the relevant topics. | Unclear |  |  |  |  |  |  |  |  |  |
| Chen et al. 2021 (9) | China | 38 | 24/14 | 67.03±7.26 | MBE | Patients will engage in meditation once in the morning and once in the evening for 5 minutes each session. Training will include exercises based on a yoga regimen, lasting 20 minutes per session, conducted four times daily. The training intensity should be adjusted to the patient's tolerance. | 12 | 4 | 20 | 6WMT/LVEF | Under supervision | Guidelines for Integrated Traditional Chinese and Western Medicine in the Diagnosis and Treatment of Acute Myocardial Infarction | AMI | Echocardiography | PCI;  routine medications (specific drugs not specified) | NR |
|  | China | 37 | 23/14 | 66.37±7.45 | Control (B) | The control group will receive standard nursing care. | 12 |  |  |  |  |  |  |  |  |  |
| Lu 2022 (10) | China | 48 | 25/23 | 65.3±2.45 | MBE | In addition to the control group, interventions involving Tai Chi and Ba Duan Jin will be implemented:Days 2 to 3 Postoperatively: Patients will be guided to watch instructional videos on Tai Chi and Ba Duan Jin, learning basic techniques.Days 4 to 7 Postoperatively: Patients will use 0.5 kg and 1.0 kg rubber Tai Chi balls as exercise tools. The regimen will include eight movements: Tai Chi starting pose, Moon-holding pose, Wrist-threading pose, Clock-lifting pose, Line-turning pose, Upper frame pose, Sun-facing pose, and Closing pose, practiced for 15 to 30 minutes per session, three times daily.Day 8 Postoperatively: The Ba Duan Jin routine will commence, comprising eight exercises: Hands supporting the sky, Regulating the spleen and stomach, Drawing the bow, Reaching for the feet, Shaking head and tail, Bouncing backward, Looking back at past strains, and Clenching fists to generate energy, performed for 15 to 30 minutes per session, three times daily.Warm-up and Cool-down: Prior to Tai Chi and Ba Duan Jin sessions, a 10-minute warm-up involving upper and lower limb stretches will be conducted, followed by a 10-minute cool-down after the exercises, each lasting 15 to 30 minutes, three times daily.Discharge Instructions: A video disc featuring Tai Chi and Ba Duan Jin exercises will be provided upon discharge. Patients will be encouraged to exercise at a comfortable level and will receive follow-up phone calls every two weeks to monitor their activity. Patients will be advised to schedule regular outpatient check-ups, with continued intervention lasting for three months. | 12 | 3 | 15-30 | 6WMT/LVEF/LVEDD/LVESD | Under supervision | Guidelines for Integrated Traditional Chinese and Western Medicine in the Diagnosis and Treatment of Acute Myocardial Infarction | AMI | Echocardiography | PCI | incidence of adverse cardiovascular events; compliance not specified |
|  | China | 48 | 27/21 | 66.07±2.84 | Control (B) | Rehabilitation Nursing Interventions:Health Education and Psychological Support: Provide comprehensive health education to patients and offer psychological counseling to address emotional and mental well-being.Cardiac Rehabilitation Exercises: Implement structured cardiac rehabilitation exercises tailored to the patient's condition, promoting physical recovery and overall cardiovascular health. | 12 |  |  |  |  |  |  |  |  |  |
| Deng et al. 2018 (11) | China | 57 | 31/26 | 64.7±4.2 | MBE | 42-Form Chen Style Tai Chi Practice:A combined approach will be adopted, incorporating training sessions in the department alongside the distribution of Tai Chi exercise DVDs for home practice. Initially, patients will practice a set of movements for approximately 10 minutes, during which physicians will monitor heart rate and observe for any discomfort. Gradually, the exercise intensity will be increased. Heart rate will be continuously monitored using a blood pressure device during exercise, with a target intensity set at an increase of 10% to 20% above resting heart rate. Follow-up phone calls will be conducted every two weeks to assess symptoms, outpatient visits, and to supervise exercise adherence. | 24 | 5 | 40-50 | 6WMT/LVEF | Under supervision | Meet the confirmed diagnostic criteria for acute myocardial infarction. | AMI | Echocardiography | PCI | NR |
|  | China | 56 | 29/27 | 57.2±4.9 | Control (B) | The control group will receive lifestyle guidance and recommendations for daily activity exercises in addition to their medication treatment. | 24 |  |  |  |  |  |  |  |  |  |
| Feng et al. 2021 (12) | China | 60 | 38/22 | 60.1±4.9 | MBE | Wuqinxi Micro-Exercises:This program involves mimicking five animals—frog, goose, cat, bear, and chicken—through a series of movements. The routine begins with a 60-second hand-warming exercise, followed by the specific movements: "Gazing at the Sky from the Well," "Swan Playing in Water," "Bear Waist Twists," "Wealth-Cat Stretch," and "Golden Rooster Standing on One Leg." Each movement will last for 30 to 60 seconds. If the patient has poor balance, the "Golden Rooster" exercise can be performed while holding onto a support. The total exercise duration will be approximately 6 minutes per session, with patients encouraged to complete 1 to 2 sets daily. The intensity and duration of the exercises will be gradually increased based on the patient's response and progress. | 24 | 5 | 30-45 | LVEF/6MWT/LVEDD | Under supervision | Guidelines for Integrated Traditional Chinese and Western Medicine in the Diagnosis and Treatment of Acute Myocardial Infarction | AMI | Echocardiography | PCI;  Usual care | In the MCET group, 7 cases experienced discomfort after exercise or under other circumstances, which was relieved after adjustment; in the MBE group, 10 cases experienced discomfort after exercise and other conditions, which was relieved after adjustment. |
|  | China | 60 | 35/25 | 60.5±5.4 | MCET | Aerobic and Resistance Exercises:The rehabilitation program will include aerobic activities such as walking and jogging, alongside resistance exercises using dumbbells and resistance bands. | 24 | Unclear | Unclear |  |  |  |  |  |  |  |
| liu 2023 (13) | China | 45 | 32/10 | 55.38±9.02 | MBE | The "Seated Baduanjin" follows these principles: each day, adjust the movements based on the patient's condition and skill level, gradually increasing the exercises in an orderly manner. The routine includes:Hands supporting the sky to regulate the sanjiao.Left and right bow like shooting an eagle.Single lift to harmonize the spleen and stomach.Look back to alleviate fatigue and injuries.Shake head and tail to eliminate heart fire.Hands grasping feet to strengthen kidneys and lower back.Clenched fists and fierce eyes to boost energy.Seven bumps on the back to dispel illnesses. | 12 | 3-5 | 50 | 6MW/LVEF | Under supervision | Fourth Universal Definition of Myocardial Infarction (2018) | AMI | Echocardiography | PCI;  Usual care | In the AE group, 1 case experienced palpitations during in-hospital phase I rehabilitation, which was relieved after rest. |
|  | China | 45 | 30/12 | 52.05±9.81 | AE | Overall principle: Gradually and systematically increase the amount of exercise, progressing from passive/active movements in bed, to sitting at the bedside, standing by the bed, moving around the bed, and finally exercising within the ward. Activities can be performed once in the morning and once in the afternoon, lasting from a few minutes to over ten minutes each time. The exercise intensity should be enough to feel slightly fatigued, with close monitoring and accompaniment by medical staff. | 12 | 3-5 | 50 |  |  |  |  |  |  |  |
| Zhou et al. 2021 (14) | China | 50 | 27/23 | 57.87± 4.61 | MBE | Baduanjin practice is conducted twice daily, from 8:00 to 8:30 AM and from 6:00 to 6:30 PM, with each session lasting about 30 minutes. Upon discharge, the ward nurses will provide the joint group patients with a homemade "Baduanjin" instructional video, a rehabilitation exercise manual, and a patient exercise log. Patients are advised to exercise once in the morning and once in the afternoon, with each session lasting 30 minutes. | 12 | 7 | 30 | 6MWT/LVEF/LVEDD/LVESD | Under supervision | Chinese Guidelines for the Prevention of Cardiovascular Diseases (2017) | AMI | Echocardiography | PCI | NR |
|  | China | 50 | 31/19 | 58.41±4.52 | Control (B) | For the routine group, patients with heart failure after myocardial infarction will receive standard treatment and care, including basic medical treatment, dietary guidance, and psychological support. | 12 |  |  |  |  |  |  |  |  |  |
| Li et al. 2018 (15) | China | 53 | 28/25 | 61.27±10.39 | MBE | In addition to medication, the exercise regimen includes Baduanjin movements: | 4 | 6 | 40 | LVEF | Under supervision | Guidelines for the Diagnosis and Treatment of Acute Myocardial Infarction | AMI | Echocardiography | All patients were given symptomatic treatments such as angiotensin-converting enzyme inhibitors, statins, nitrates, low-molecular-weight heparin, and antiplatelet aggregation therapy, and the control group was additionally given Tongxinluo. | NR |
|  | China | 53 | 30/23 | 61.38±10.21 | Control (A) | In addition to standard treatment, Tongxinluo capsules will be used. | 4 |  |  |  |  |  |  |  |  |  |
| Wu et al. 2023 (16) | China | 60 | 34/26 | 55.38±1.05 | MBE | The observation group will receive intervention with Baduanjin combined with interactive games, similar to the control group. The Baduanjin exercises include: | 4 | 1-2 | 20-30 | LVEF/LVEDD/LVESD | Under supervision | Guidelines for Integrated Traditional Chinese and Western Medicine in the Diagnosis and Treatment of Acute Myocardial Infarction | AMI | Echocardiography | PCI | NR |
|  | China | 60 | 35/25 | 55.35±1.02 | MCET | Lipped breathing and abdominal breathing exercises will be conducted in the first week of admission, lasting 20 minutes per session, 2-3 times per week. Cycling will simulate environments like buildings and tracks, transitioning from a stationary to an active state with adjusted speeds. Starting in the second week, cycling will last 20-30 minutes, once a week. Additionally, activities like bowling and playing tennis will begin in the second week, with sessions lasting 20-30 minutes, twice a week. | 4 | 1-2 | 20-30 |  |  |  |  |  |  |  |
| Zong et al. 2022 (17) | China | 50 | 23/27 | 57.26±6.84 | MBE | The observation group will receive Baduanjin in addition to the control group’s interventions. The Baduanjin program consists of two phases:Phase 1 (Postoperative Days 1-5): Performed in a supine or seated position, including five movements: left and right bow like shooting an eagle, look back to alleviate fatigue, hands supporting the sky, single lift to harmonize the spleen and stomach, and clenched fists to boost energy. The exercise intensity will be 1-2 METs, with one session of 30 minutes daily.Phase 2 (Postoperative Day 6 to discharge): Performed in a standing position, incorporating all traditional Baduanjin movements. The exercise intensity will be 3-5 METs, with one session of 30 minutes daily, continuing for two weeks post-surgery. | 2 | 7 | 30 | LVEF/LVEDD/LVESD | Under supervision | Guidelines for the Diagnosis and Treatment of Acute ST-Segment Elevation Myocardial Infarction | AMI | Echocardiography | PCI;  routine medications (specific drugs not specified) | incidence of adverse cardiovascular events; compliance not specified |
|  | China | 50 | 21/29 | 56.91±7.34 | MCET | Postoperative Day 1: Health education and psychological intervention. In addition, perform straight leg raises in a supine position with legs apart, lifting to 30°, 5 times per set, once in the morning and once in the afternoon.Postoperative Day 2: Raise both arms to the side of the head while taking deep breaths, exhaling slowly, 5 times per set, once in the morning and once in the afternoon.Postoperative Day 3: Sit up in bed for 5 minutes, twice a day.Postoperative Day 4: Stand by the bed for 5 minutes, twice a day.Postoperative Day 5: Walk by the bed for 5 minutes, twice a day.Postoperative Day 6**: Engage in indoor activities for 10 minutes, twice a day.Postoperative Day 7 until discharge**: Continue psychological intervention and conduct a 6-minute walking test. | 2 | 7 | 5-25 |  |  |  |  |  |  |  |
| Guo 2023 (18) | China | 30 | 21/9 | 64.10±5.58 | MBE | The training for seated Baduanjin starts with 2 sets per session, performed twice daily. If the patient's condition remains stable after two days, they may progress to seated Baduanjin with 4 sets, twice daily. If stable for three days, they can transition to standing Baduanjin, also with 4 sets, twice daily.After that, under medical guidance, the training duration for standing Baduanjin will gradually increase based on the patient's specific condition. One month after heart failure, the training will be standardized to 8 sets of standing Baduanjin, twice daily, continuing this regimen until three months post-heart failure. | 12 | 14 | 25 | LVEF/6MWT | Under supervision | 2020 Expert Consensus on Prevention and Treatment of Heart Failure after Myocardial Infarction | AMI | Echocardiography | PCI;  Usual care | NR |
|  | China | 30 | 23/7 | 59.60±5.78 | Control (A) | Antiplatelet aggregation; lipid regulation to stabilize plaques; inhibition of the RASS system to improve remodeling; use of ACE inhibitors, ARBs, or ARNIs; and individualized treatment based on blood pressure and blood glucose levels. | 12 |  |  |  |  |  |  |  |  |  |
| Zhang et al. 2023 (19) | China | 60 | 30/30 | 53.8±14.2 | MBE | From postoperative days 2 to 5, guide the patient in rehabilitation exercises. On days 6 and 7, instruct them in traditional Baduanjin practice: stand naturally with a calm mind, feet shoulder-width apart and slightly bent, hands in front of the abdomen, breathing naturally and focusing on the lower abdomen.Based on the patient's exercise tolerance, practice 1 to 3 sets per session, 3 times a day, for 20 to 30 minutes each time. After discharge, they should begin independent practice of 2 to 3 sets per session for about 40 minutes, 5 days a week, for a duration of 3 months, with follow-up at 3 months. | 12 | 3-5 | 40 | LVEF | Under supervision | Guidelines for the Diagnosis and Treatment of Acute Myocardial Infarction | AMI | Echocardiography | PCI;  Routine treatment (calcium channel blockers, lipid-lowering drugs, etc.) | NR |
|  | China | 60 | 30/30 | 52.4±13.6 | Control (B) | Calcium channel blockers, lipid-lowering agents, nitrates, and beta-blockers will be administered for continuous treatment over 3 months, with follow-up after 3 months. | 12 |  |  |  |  |  |  |  |  |  |
| Liu  2023 (20) | China | 44 | 23/21 | 67.24±8.28 | MBE | ①Raise and Lower②Left and Right Bow③Forward Bend and Backward Stretch④Heel Raises and Toe Taps | 4 | 3-4 | 20 | LVEF/LVEDD | Unclear | Expert Consensus on Integrated Traditional Chinese and Western Medicine in the Diagnosis and Treatment of Acute Myocardial Infarction | AMI | Echocardiography | PCI | NR |
|  | China | 43 | 28/15 | 67.31±8.34 | Control (B) | The control group will receive standard postoperative interventions, including guidance on routine medication, monitoring of various health indicators, psychological support, health education, and explanations of precautions. | 4 |  |  |  |  |  |  |  |  |  |
| Yao 2023 (21) | China | 56 | 34/22 | 67.75±7.13 | MBE | The simplified version of Baduanjin consists of 8 movements, each performed 8 times, with each session lasting 12 minutes. The total practice time per day should be controlled between 30 to 60 minutes, based on the patient's tolerance. | 24 | 7 | 30-60 | LVEF/LVEDD/LVESD/6MWT | Unclear | Guidelines for the Diagnosis and Treatment of Acute Myocardial Infarction | AMI | Echocardiography | PCI;  Aspirin, clopidogrel, and atorvastatin calcium were administered orally. | NR |
|  | China | 56 | 32/24 | 67.43±7.01 | Control (A) | Aspirin (100 mg once daily), clopidogrel (75 mg once daily), and atorvastatin calcium (10 mg once daily) will be administered orally. After 6 months of continuous treatment, the effectiveness will be assessed. | 24 |  |  |  |  |  |  |  |  |  |
| Ming-Gui Chen et al. 2020 (22) | China | 43 | 29/14 | 59.98±10.86 | MBE | Seated Baduanjin practice will be conducted for 30 minutes per session, twice daily, with each cycle lasting 3 days. After discharge, patients will transition to standing Baduanjin practice for 30 minutes per session, 5 times a week, for a duration of 24 weeks. | 24 | 5 | 30 | LVEF | Under supervision | The Third Universal Definition of Myocardial Infarction | AMI | Echocardiography | PCI | NR |
|  | China | 39 | 30/9 | 61.49±11.54 | Control (B) | Maintain the original living habits | 24 |  |  |  |  |  |  |  |  |  |
| Małgorzata Grabara et.al 2020 (23) | Poland | 35 | 35/0 | 57.1 ± 5.3 | MBE | Simple poses: 1 minute.Breathing exercises (adjusting breath): 5 minutes.Sun Salutation: 5 minutes.Yoga relaxation: 2 minutes.Shoulder stand: 1 minute.Fish pose: 1 minute.Head-to-knee pose: 1 minute.Cobra pose: 1 minute.Seated half spinal twist: 1 minute.Standing forward bend: 1 minute.Extended triangle pose: 1 minute.Yoga relaxation: 5 minutes.Breathing exercises (adjusting breath): 4 minutes.Relaxation pose: 1 minute | 6 | 7 | 30 | LVEF/LVEDD/LVESD | Under supervision | Meets the diagnostic criteria for ST-elevation myocardial infarction | ST- segment elevation MI | Echocardiography | PCI | NR |
|  | Poland | 35 | 35/0 | 49.6 ± 5.03 | Control (B) | According to the routine cardiac rehabilitation program recommended by the European Society of Cardiology (ESC) | 6 |  |  |  |  |  |  |  |  |  |
| Shi et al. 2022 (24) | China | 103 | 76/27 | 65.27±9.87 | AE | (1) Aerobic exercise training should begin as soon as the patient can get out of bed, starting with slow walking at the bedside, assisted by medical staff or family. Walking duration should be 5-10 minutes per session, 1-2 times per day.(2) Based on the patient's recovery and improvements in VO2max, gradually increase exercise intensity, including walking outside the ward or stair climbing, extending training duration to 10-20 minutes per session, 1-2 times per day.(3) After discharge, continue aerobic exercise training. For the first 1-4 weeks post-discharge, set the exercise intensity at 40-50% of the patient's VO2max, combining slow and brisk walking for 20-30 minutes per session, once daily.(4) For weeks 5-8 post-discharge, increase exercise intensity to 50-60% of VO2max, incorporating brisk walking and stationary cycling, with each training session lasting 20-30 minutes, once daily.(5) For weeks 9-12 post-discharge, further increase exercise intensity to 60-70% of VO2max, with each training session lasting 30 minutes, once daily. | 12 | 7 | 20 | LVEF/6MWD/LVEDD | Under supervision | Guidelines for the Diagnosis and Treatment of Acute Myocardial Infarction (2019) | MI | Echocardiography | PCI;  Routine drug therapy (antiplatelet, lipid-lowering, etc.) | NR |
|  | China | 103 | 70/33 | 65.18±9.91 | Control (B) | Strengthen health education, monitoring of physiological indicators, medication guidance, dietary guidance, and routine rehabilitation training guidance. | 12 |  |  |  |  |  |  |  |  |  |
| Peng et al. 2023 (25) | China | 65 | 26/29 | 48.64±8.27 | AE | Aerobic exercise will be conducted using a steady pace walking method on flat surfaces. Before starting, ensure proper warm-up exercises, including 4 minutes for breathing adjustments and stretching. Walk at a speed of approximately 40 meters per minute for 200 meters, followed by a 2-minute rest. Gradually increase the speed to 60-70 meters per minute to walk 2000 meters or maintain continuous exercise for 20-30 minutes, resting for 1 minute after each walking session. Depending on the patient's tolerance, incorporate low-intensity activities like fitness exercises or Tai Chi. After completing the session, focus on calming the breath and stretching for 4 minutes each. It is strictly prohibited to sit, lie down, or take a bath immediately after exercising. | 24 | 10 | 30 | LVEF/LVEDD/LVESD | Unclear | Meets the relevant diagnostic criteria for acute myocardial infarction | AMI | Echocardiography | PCI;  Usual care | NR |
|  | China | 55 | 28/27 | 49.53±8.25 | Control (A) | The control group will receive standard treatment, including anticoagulation and antiplatelet aggregation therapy. | 24 |  |  |  |  |  |  |  |  |  |
| Zhou et al. 2016 (26) | China | 24 | 15/9 | 60.82±8.37 | AE | On postoperative days 1 and 2, passive activities will be conducted: while in bed, the affected limb will be immobilized, and passive movements of the joints will be performed, including ankle and elbow joints once per hour, with 3 movements each time, for a total of 6 times. After immobilization is removed, the patient will sit up in bed 4 times and stand by the bedside 2 times, each for 5 minutes, using a bedside commode.On day 3, the patient will sit by the bedside 4 times, each for 30 minutes, and walk twice daily for 50 meters each time. On day 4, the patient will continue sitting by the bedside 4 times and increase walking to 2-3 times daily for 70 meters each time. All activities will be performed under continuous ECG monitoring.From days 5 to 7, the patient will sit freely and walk 3 times daily for 100 meters each time. On day 8, a cardiopulmonary exercise test (CPET) will be conducted. | 24 | 4-5 | Unclear | LVEF/LVESD | Under supervision | Meet the diagnostic criteria for acute myocardial infarction established by the WHO. | ST- segment elevation MI | Echocardiography | PCI;  routine medications (specific drugs not specified) | NR |
|  | China | 20 | 14/6 | 61.6±8.71 | Control (B) | After removing the immobilization from the surgical site, the patient will be instructed to engage in light, unquantified activities in bed.In-room rehabilitation activities:Days 3-4: The patient may stand by the bedside, sit up, and perform personal hygiene activities in bed.Days 5-7: The patient can leave the bedside for toileting and other activities under the supervision of a caregiver, though the duration is not specified.Day 8: A cardiopulmonary exercise test (CPET) will be conducted. | 24 |  |  |  |  |  |  |  |  |  |
| Yan et al. 2021 (27) | China | 41 | 22/19 | 60.03±9.13 | AE | In addition to standard Western medical treatment, aerobic exercise therapy will be implemented. | Unclear | Unclear | Unclear | LVEF/6MWD/LVEDD/LVESD | Unclear | Guidelines for the Diagnosis and Treatment of Acute ST-Segment Elevation Myocardial Infarction | AMI | Echocardiography | PCI;  Routine medications (Bayaspirin Enteric-coated Tablets, atorvastatin, clopidogrel tablets, enoxaparin sodium injection, etc.) | NR |
|  | China | 41 | 26/15 | 59.40±7.66 | Control (A) | The control group will receive standard Western medical treatment on postoperative day 2. | Unclear |  |  |  |  |  |  |  |  |  |
| Zhou et al. 2017 (28) | China | 25 | 9/16 | 54.82±8.37 | AE | During postoperative days 1-2, the surgical site will be immobilized while the patient is in bed. The patient will walk 2-3 times daily for 70 meters. From postoperative days 5-7, the patient will walk 3 times daily for 100 meters. On day 8, a cardiopulmonary exercise test (CPET) will be conducted. After the assessment, patients in the exercise group will continue aerobic rehabilitation treatment upon discharge, engaging in brisk walking or jogging at a pace above their anaerobic threshold. | 12 | 4 | 20 | LVEF/LVESD | Under supervision | Meet the diagnostic criteria for acute myocardial infarction established by the WHO. | ST- segment elevation MI | Echocardiography | PCI | NR |
|  | China | 25 | 6/19 | 59.60±8.71 | Control (B) | Patients in the control group may engage in daily activities as needed and will have monthly outpatient follow-ups, including physical examinations and medication management. | 12 |  |  |  |  |  |  |  |  |  |
| Ji et al. 2017 (29) | China | 36 | 20/16 | 50.9±6.6 | AE | Before and after aerobic rehabilitation training, there will be 5 minutes dedicated to warm-up and cool-down exercises. The main activity will consist of 30 minutes of aerobic walking, performed 3 times a week for a duration of 3 months. The control group will not participate in any aerobic rehabilitation training. | 12 | 3 | 30 | LVEF/6MWD/LVESD | Under supervision | Guidelines and Consensuses for the Prevention and Treatment of Cardiovascular Diseases (2014 Edition) | MI | Echocardiography | PCI | NR |
|  | China | 40 | 26/14 | 50.8±6.5 | Control (B) | The control group will receive standard treatment, including anticoagulation and antiplatelet aggregation therapy. | 12 |  |  |  |  |  |  |  |  |  |
| Zhou et al. 2021 (30) | China | 61 | 34/27 | 57.62±5.79 | AE | From postoperative days 3 to 6, patients will be guided to perform active movements of joints and limbs, with family members assisting in bedside sitting and standing exercises. After training, massage of the major muscle groups will be encouraged.From days 7 to 9, patients will be guided to independently perform activities such as sitting, standing, personal hygiene, and eating, with assistance for indoor walking training.From days 10 to 15, family members will assist patients in walking in the ward or going up and down stairs.From days 16 to 30, as patients' symptoms significantly improve, they will be encouraged to freely choose aerobic rehabilitation activities, including stair climbing, walking, Tai Chi, jogging, and aerobic exercises. | 12 | 10-14 | 40 | LVEF/6MWT | Under supervision | Diagnosed as acute myocardial infarction | AMI | Echocardiography | PCI;  routine medications (specific drugs not specified) | NR |
|  | China | 61 | 33/28 | 53.67±5.93 | Control (B) | ①Basic Care.②Discharge Instructions: Provide patients with a disease management manual, advising them to follow a low-salt, light diet with small, frequent meals. They should avoid overeating, intense exercise, and emotional stress. If they experience increased pain, they should seek medical attention promptly. | 12 |  |  |  |  |  |  |  |  |  |
| Jiang et al. 2022 (31) | China | 31 | 17/14 | 65.48±3.29 | AE | Aerobic Rehabilitation Exercise.During Hospitalization:Days 1-2 Post-Intervention: Perform passive limb movements, including ankle, knee, and elbow flexion/extension exercises and muscle stretching. Conduct these exercises 6 times a day for 10 minutes each.Bedside Training: Practice sitting up in bed and standing by the bedside twice a day for 5 minutes each.Days 3-4 Post-Intervention: Engage in sitting exercises and walking training in bed, twice a day for 30 minutes each.From Day 5 Onward: Increase the frequency of sitting and walking exercises to 3-4 times a day, extending the duration to 50 minutes each time.After Discharge:Patients will engage in brisk walking or jogging for exercise, 4 times a week for 20-30 minutes per session. Regular follow-ups will be conducted to monitor the patients' exercise progress and overall health status. | Unclear | 4 | 20-30 | LVEF/LVESD | Under supervision | Clinically diagnosed as acute myocardial infarction | AMI | Echocardiography | PCI;  routine medications (specific drugs not specified) | NR |
|  | China | 31 | 16/15 | 65.87±3.33 | Control (B) | Instruct patients to maintain a balanced diet, adhere to prescribed medications, open windows for ventilation at regular times each day, and keep their hospital room clean. | Unclear |  |  |  |  |  |  |  |  |  |
| Chen et al. 2019 (32) | China | 51 | 32/19 | 49.8±6.5 | AE | The exercise plan will be based on the target heart rate for moderate intensity. Prior to and following aerobic rehabilitation training, 5-10 minutes of warm-up and cool-down exercises will be implemented. The main activity will consist of 30 minutes of aerobic walking, performed once every two days. | 48 | 14 | 30 | LVEF/LVESD | Under supervision | Guidelines and Consensuses for the Prevention and Treatment of Cardiovascular Diseases (2014 Edition) | AMI | Echocardiography | PCI;  routine medications (specific drugs not specified) | NR |
|  | China | 51 | 31/20 | 49.7±6.4 | Control (B) | During hospitalization, patients will receive symptomatic treatment. After discharge, they should take medications as prescribed and attend regular follow-up appointments. They should maintain regular bowel movements, avoid emotional stress and excessive fatigue, refrain from smoking and limit alcohol intake, and follow a balanced diet. | 48 |  |  |  |  |  |  |  |  |  |
| Yan et al. 2021 (33) | China | 44 | 26/18 | 60.21±8.93 | AE | The exercise intensity will be based on 80% of the maximum heart rate reserve for cardiovascular patients, with sessions lasting 20 minutes each, conducted 3 times a week. The cycling program is divided into three phases:Preparation Phase: Patients will perform static stretching exercises to provide light stimulation to the neuromuscular system for about 5 minutes.Exercise Phase: Starting with a load of 0 watts, the resistance will be increased by 10 watts every 2-3 minutes until reaching the target heart rate (80% of the maximum heart rate) for a total of 20 minutes.Recovery Phase: Gradually decrease the resistance while continuing to cycle for an additional 3-10 minutes. | 4 | 3 | 20 | LVEF/LVESD | Under supervision | Guidelines and Consensuses for the Prevention and Treatment of Cardiovascular Diseases (2014 Edition) | AMI | Echocardiography | PCI;  Routine medications (aspirin enteric-coated tablets, atorvastatin calcium tablets, clopidogrel hydrogen sulfate tablets, etc.) were used after surgery. | NR |
|  | China | 44 | 27/17 | 60.39±8.43 | Control (A) | The control group will receive standard medication on postoperative day 2, including enteric-coated aspirin 100 mg, atorvastatin 10 mg, and clopidogrel 75 mg, administered once daily. | 4 |  |  |  |  |  |  |  |  |  |
| Hu et al. 2024 (34) | China | 40 | 25/15 | 55.20±5.84 | AE | Group A will undergo an incremental load test followed by aerobic exercise. | 12 | 3 | 40 | LVEF/LVEDD | Under supervision | Guidelines for the Diagnosis and Treatment of Acute ST-Segment Elevation Myocardial Infarction (2019) | ST- segment elevation MI | Echocardiography | PCI;  After surgery, the patients received routine treatments such as antiplatelet therapy, blood pressure lowering, and blood glucose lowering. | NR |
|  | China | 40 | 26/14 | 57.23±6.32 | Control (A) | After PPCI and upon discharge, routine treatments will include antiplatelet therapy, antihypertensive medications, glucose-lowering agents, and interventions to reduce myocardial oxygen demand. Medications will be tailored to the patient's specific conditions, including lipid-lowering treatment with atorvastatin (1 tablet orally every night) and Livalo (Pitavastatin). | 12 |  |  |  |  |  |  |  |  |  |
| Yao 2019 (35) | China | 42 | 1.33 | 58.14±6.43 | AE | Instruct patients to perform passive and active exercises for their limbs while in bed, twice daily for 5-10 minutes each session.Guide patients in sitting up training in bed, twice daily for 5-10 minutes each session.Encourage patients to practice marching in place by the bedside, twice daily for 5-10 minutes each session.Guide patients in bedside walking training, twice daily, covering approximately 30 meters.each time.Instruct patients to engage in walking training, twice daily, covering about 150 meters each time.Guide patients in walking and stair training, twice daily, walking about 200 meters and going up and down half a flight of stairs.Continue to guide patients in walking and stair training, twice daily, covering around 200 meters each time. | Unclear | 14 | 10 | LVEF | Under supervision | Diagnosed as acute myocardial infarction | AMI | Echocardiography | PCI;  Usual care | NR |
|  | China | 42 | 23/19 | 58.36±6.29 | Control (B) | Provide daily care activities (such as managing bowel and bladder needs and personal hygiene). For patients transferred to general wards, guide them in bed activities (like flexing, extending, and elevating limbs) as well as activities out of bed (such as standing by the bedside and walking). | Unclear |  |  |  |  |  |  |  |  |  |
| Qin et al. 2023 (36) | China | 54 | 29/25 | 67.46±10.08 | AE | Based on the patient's age, physical condition, and usual activity level, a tailored exercise plan will be developed. Patients are advised to engage in brisk walking or slow jogging about 1.5 hours after meals. During exercise, monitor heart rate, aiming for a target heart rate of 70% to 85%. Each exercise session should last 30 minutes, occurring 3 times a week for a duration of 6 months. | 24 | 3 | 30 | LVEF/LVESD/LVESD/ | Unclear | Diagnosed as acute myocardial infarction | MI | Echocardiography | Take beta-blockers, aspirin enteric-coated tablets and other drugs regularly. | NR |
|  | China | 54 | 28/26 | 69.14±10.13 | Control (B) | Routine Care:Closely monitor the patient's vital signs.Provide education on disease-related knowledge.Instruct the patient on the correct use of medications, including beta-blockers, enteric-coated aspirin, statins, and clopidogrel.Offer dietary guidance. | 24 |  |  |  |  |  |  |  |  |  |
| Jiang et al. 2006 (37) | China | 35 | Unclear | Unclear | AE | Exercise Plan:Cycling: Start with 6-10 minutes, 2 times a week, gradually increasing to 20-30 minutes, 5-6 times a week.Walking: Incorporate walking sessions alongside cycling to enhance overall fitness. | 12 | 5 | 20-30 | LVEF/LVDD/ | Under supervision | Guidelines and Consensuses for the Prevention and Treatment of Cardiovascular Diseases | AMI | Echocardiography | PCI;  Usual care | NR |
|  | China | 29 | Unclear | Unclear | Control (B) | The control group will receive standard treatment, including anticoagulation and antiplatelet aggregation therapy. | 12 |  |  |  |  |  |  |  |  |  |
| Muthukrishnan et al. 2021 (38) | UAE | 12 | 11/1 | 49±8.43 | AE | All participants in the power walking group are instructed to keep their backs straight, swing their shoulders comfortably, and bend their elbows at 90°. They will gradually increase their speed, starting from a slow pace to brisk walking and then to power walking, initially on flat ground. | 4 | 3 | 40-70 | LVEF/6MWT | Under supervision | Meets the relevant diagnostic criteria for acute myocardial infarction | AMI | Echocardiography | PCI;  Usual care | NR |
|  | UAE | 12 | 11/1 | 48.41±6.69 | Control (B) | The control group will receive standard treatment, including anticoagulation and antiplatelet aggregation therapy. | 4 |  |  |  |  |  |  |  |  |  |
| Gremeaux et al. 2011 (39) | French | 8 | 7/1 | 65.8±9 | AE | For the 6-Minute Walk Test (6MWT), aim for an 18-minute target heart rate (THR) for continuous exercise. | 6 | 3 | 80 | 6MWT/VO2peak | Under supervision | Meets the relevant diagnostic criteria for acute myocardial infarction | ST- segment elevation MI or non -ST- segment elevation MI | Echocardiography | PCI;  Drug therapy (beta-blockers, ACEI/ARB, antiplatelet drugs, statins) | NR |
|  | French | 9 | 7/2 | 59.2±8.1 | HIIT | Use the maximum heart rate (HR) from the 200-meter walk test (mfwt) for the peak intervals in HIIT training, and the maximum HR from the 6-Minute Walk Test (6MWT) for active recovery. After reaching 50% of the graded maximum exercise test HR for 5 minutes, the patient will complete an 18-minute exercise session consisting of three consecutive 6-minute phases. Each phase will include 2 minutes at the 200 mfwt maximum HR, followed by 4 minutes of active recovery at the 6MWT maximum HR. The session will conclude with a 3-minute cool-down period. | 6 | 3 | 80 |  |  |  |  |  |  |  |
| Zhang et al. 2022 (40) | China | 42 | 24/18 | 55.24±9.75 | AE | Aerobic exercise will be conducted using fixed bikes, jogging, and treadmills. Before starting, the 6-Minute Walk Distance (6MWD) test will be used to assess exercise intensity:If 6MWD < 300 m: Walk for 20 minutes, 3 times a week.If 6MWD ≥ 300 m: Use the treadmill or fixed bike for 20 minutes, 5 times a week. | 4 | 3-5 | 20 | LVEF/6MWT/LVEDD/LVESD | Under supervision | Guidelines for the Diagnosis and Treatment of Acute Myocardial Infarction | AMI | Echocardiography | PCI;  anticoagulant therapy | NR |
|  | China | 42 | 26/16 | 54.91±9.82 | Control (B) | The control group will receive standard interventions, which include general assessments, medication management, dietary guidance, psychological support, and exercise interventions. Tailored activity and exercise instructions will be provided based on the individual circumstances of each patient after discharge. | 4 |  |  |  |  |  |  |  |  |  |
| Bruna C.Matos-Garcia et al. 2022 (41) | Brazil | 31 | 22/9 | 55.90±14.60 | AE | In the 60 days following a heart attack, participants should engage in progressive walking on the PEB at least 4 times per week, consisting of 4-5 sessions. The exercise plan includes three phases:Warm-up: 5 minutes.Endurance Training: 20 minutes, increasing the walking duration by 5 minutes each week.Cool-down: 5 minutes.Continue this routine until reaching 60 minutes per session. | 8 | 4 | 60 | 6MWT | Under supervision | Meets the relevant diagnostic criteria for acute myocardial infarction | ST- segment elevation MI or non -ST- segment elevation MI | Echocardiography | PCI;  routine medications (specific drugs not specified) | NR |
|  | Brazil | 23 | 17/6 | 55.80±7.50 | Control (B) | Routine nursing care: guidance on physical activity, nutrition, and medication therapy initiated during hospitalization | 8 |  |  |  |  |  |  |  |  |  |
| Thatiana C.A.Peixoto,MSc et al. 2015 (42) | Brazil | 45 | 33/12 | 56.80±10.80 | AE | To the control group were added ( 1 ) a warm-up period of 5 min; ( 2 ) an endurance training period, in which the walking time was progressively increased from 20 min in week 1 to 25 min in week 2 to 30 ~ 35 min in week 3 and 35 ~ 40 min in the last week; and ( 3 ) a cool-down period of 5 min. | 4 | 4 | 35-40 | 6MWT | Under supervision | Meets the relevant diagnostic criteria for acute myocardial infarction | ST- segment elevation MI or non -ST- segment elevation MI | Echocardiography | PCI;  routine medications (specific drugs not specified) | NR |
|  | Brazil | 43 | 29/14 | 56.00±9.60 | Control (B) | Routine nursing care: guidance on physical activity initiated during hospitalization, without a specific rehabilitation plan | 4 |  |  |  |  |  |  |  |  |  |
| Cai et al. 2021 (43) | China | 30 | 30/0 | 55 ± 9 | AE | Individualized exercise prescription for the patient by a rehabilitation physician based on CPET results | 24 | Unclear | Unclear | LVEF | Under supervision | ACC/AHA Guidelines | ST- segment elevation MI | Echocardiography | PCI;  Routine secondary prevention medications (aspirin, clopidogrel, etc.) | NR |
|  | China | 30 | 30/0 | 58 ± 8 | Control (B) | Patients in the control group were not given any exercise prescription and exercise was not intervened. Patients in both groups were followed up by telephone every 2 weeks and outpatient follow-up once a month until the end of 6 months | 24 |  |  |  |  |  |  |  |  |  |
| Minghui Jiang et al. 2021 (44) | China | 49 | 31/18 | 58.79 ± 9.36 | MCET | ( 1 ) Within 1 week after surgery, the nursing staff demonstrated and instructed the patient to start alternating fast and slow breathing exercises at the bedside, 5 ~ 6 times / min for 10 min.( 2 ) On the 8th day after surgery, the nursing staff continued to carry out breathing exercises with the patient. After the patient's condition was basically stabilized, 3 types of stretching exercises were selected according to their personal preference, 5 min / times. When the patient reaches the tolerance level of the above exercises, increase the strength training, such as squatting, weight-bearing exercises, etc., 10 times/group, 3 groups/d. ( 3 ) Determine the degree of recovery of the patient's cardiac function, and increase the endurance exercises as appropriate, including upper limb and lower limb training. Upper extremity training included raising the arms and outlining the left and right cycles, 15 cycles per repetition, 3 repetitions per group. Lower extremity exercises began with walking 100 m on level ground, and the intensity gradually increased to walking | 24 | 7 | Unclear | LVEF | Under supervision | Meets the relevant diagnostic criteria for acute myocardial infarction | AMI | Echocardiography | PCI;  routine medications (specific drugs not specified) | NR |
|  | China | 49 | 33/16 | 59.62 ± 8.98 | Control (B) | After the patients were admitted to the hospital, the nursing staff distributed health education materials to the patients and their families, prepared the patients in accordance with the requirements of the department before the operation, guided the patients to get out of bed after the operation, provided exercise education before discharge, and instructed the patients to return to the outpatient clinic at the agreed time | 24 |  |  |  |  |  |  |  |  |  |
| Jonathan Myers et.al 2002  (45) | American | 12 | 10/2 | 52.8 ± 12 | AE | Performed five times per week for 45 minutes on a bicycle ergometer; the goal of exercise intensity is to maintain a level commensurate with 60% to 80% of maximal oxygen uptake | 8 | 5 | 45 | LVEF | Under supervision | Meets the relevant diagnostic criteria for acute myocardial infarction | AMI | cardiac MRI | Digoxin, ACE inhibitors, diuretics, beta-blockers and other drugs were used. | NR |
|  | American | 12 | 10/2 | 58.2 ± 6 | Control (B) | The control group will receive standard treatment, including anticoagulation and antiplatelet aggregation therapy. | 8 |  |  |  |  |  |  |  |  |  |
| Romualdo Belardinelli et.al 2001 (46) | American | 59 | 49/10 | 53 ± 11 | AE | The program lasted 6 months, with exercise performed 3 times per week at an intensity of 60% of peak oxygen uptake (V˙O2), and patients pedaled on an electronically braked bicycle ergometer for 30 minutes at a target work rate. | 24 | 3 | 30 | LVEF/LVEDD/LVESD | Under supervision | Meets the relevant diagnostic criteria for acute myocardial infarction | AMI | Echocardiography | PCI;  They received drug treatments such as aspirin, ticlopidine, calcium antagonists, and nitrates. | NR |
|  | American | 59 | 50/9 | 59 ±10 | Control (B) | Only engage in basic daily light physical activities, avoid sports training, and record daily activities. | 24 |  |  |  |  |  |  |  |  |  |
| FRANCESCO GIALLAURIA et.al 2006 (47) | Italy | 20 | 16/4 | 68.6 ± 2.3 | AE | Each session was preceded by a 5-minute warm-up, followed by a 5-minute cool-down, and 30 minutes of pedaling on a bicycle ergometer at 60% of the peak vo2 achieved during the initial symptom-limiting cardiorespiratory exercise test (CPX-1) over a 3-month period. | 12 | 3 | 30 | LVEF | Under supervision | Meets the relevant diagnostic criteria for acute myocardial infarction | AMI | Echocardiography | Drugs such as ACE inhibitors, angiotensin II receptor antagonists, beta-blockers, nitrates, and diuretics were used. | NR |
|  | Italy | 20 | 17/3 | 68.2 ± 2.6 | Control (B) | Patients in the control group were discharged from the hospital with routine instructions to continue physical activity and maintain a proper lifestyle | 12 |  |  |  |  |  |  |  |  |  |
| Francesco Giallauria et.al 2008 (48) | Italy | 30 | 23/7 | 59 ± 3 | AE | 30 minutes of exercise on a cycle ergometer with the goal of achieving 60-70% of peak oxygen consumption at initial symptom-limited CPX monitored by the wearable device, 3 times per week for 6 months. | 24 | 3 | 30 | LVEF | Under supervision | Meets the relevant diagnostic criteria for acute myocardial infarction | ST- segment elevation MI | Echocardiography | PCI;  Beta-blockers, ACE inhibitors, angiotensin receptor antagonists, antiplatelet drugs, statins and other medications were used. | There were no adverse events, and the compliance rate was 88%. |
|  | Italy | 30 | 24/6 | 58 ± 4 | Control (B) | Patients in the control group received routine instructions on continuing physical activity and maintaining a proper lifestyle at the time of discharge from the hospital, and were only observed at the 6-month follow-up visit | 24 |  |  |  |  |  |  |  |  |  |
| Zheng et.al 2008 (49) | China | 27 | Unclear | Unclear | AE | Patients in the exercise group participated in exercise training on an outpatient basis, 3 times per week. Exercise was performed for 30 minutes on a cycle ergometer and the workload was adjusted according to the CPET results of the previous 3 months. | 24 | 3 | 30 | LVEF | Under supervision | Meets the relevant diagnostic criteria for acute myocardial infarction | AMI | Echocardiography | PCI;  Routine medications (beta-blockers, ACEI, etc.) | NR |
|  | China | 30 | Unclear | Unclear | Control (B) | Control group does not practice | 24 |  |  |  |  |  |  |  |  |  |
| Tomomi Koizumi et.al 2003 (50) | Japan | 14 | 13/1 | 54±12 | AE | A 3-month training plan with daily walks of over 30 minutes; no specific walking speed is designated. | 12 | 7 | 30 | LVEF | Under supervision | Meets the relevant diagnostic criteria for acute myocardial infarction | AMI | Echocardiography | PCI;  Drugs such as calcium antagonists, ACE inhibitors, lipid-lowering drugs, ticlopidine, and aspirin were used. | NR |
|  | Japan | 15 | 13/2 | 54±12 | Control (B) | Receiving educational support without a formal exercise plan. | 12 |  |  |  |  |  |  |  |  |  |
| Cha et al. 2016 (51) | China | 40 | 27/13 | 46.26 ± 4.85 | AE | Based on heart rate (HR) for different intensities of aerobic exercise:Effective exercise heart rate (beats/min) for low intensity: (200−age−resting HR)×(40%−49%)+resting HR of low intensity group(200−age−resting HR)×(40%−49%)+resting HR of low intensity groupFor moderate intensity: (200−age−resting HR)×(50%−59%)+resting HR of moderate intensity group(200−age−resting HR)×(50%59%)+resting HR of moderate intensity groupFor high intensity: (200−age−resting HR)×(60%−69%)+resting HR of moderate intensity group(200−age−resting HR)×(6069%)+resting HR of moderate intensity groupHigh intensity aerobic exercise duration is 30 minutes per session, three times a week for 12 weeks. | 12 | 3 | 30 | LVEF | Under supervision | Meet the diagnostic criteria for acute myocardial infarction established by the WHO. | AMI | Echocardiography | PCI;  routine medications (specific drugs not specified) | NR |
|  | China | 40 | 25/15 | 46.35 ± 5.12 | Control (A) | The conventional treatment group receives standard medication and continues daily life without additional aerobic exercise. | 12 |  |  |  |  |  |  |  |  |  |
| Fatemeh Basati et al. 2012 (52) | UAE | 15 | 15/0 | 54.2 ± 9.04 | AE | Each training session lasts 60-90 minutes, including 10-20 minutes of warm-up, 20-40 minutes of aerobic exercise, and a 10-minute cool-down. Additionally, there is a 20-minute relaxation period at the end of each session. Exercise intensity is calculated as 60-85% of the maximum heart rate achieved during the exercise test, based on determined risk levels. | 8 | 3 | 60-90 | LVEF/LVEDD/LVESD | Under supervision | Meets the relevant diagnostic criteria for acute myocardial infarction | MI | Echocardiography | They received treatments such as antiplatelet drugs and lipid-lowering drugs. | NR |
|  | UAE | 14 | 14/0 | 51.71 ± 6.98 | Control (B) | Standardized Outpatient Cardiac Rehabilitation Program (SOCRP) | 8 |  |  |  |  |  |  |  |  |  |
| Mei 2022 (53) | China | 42 | 23/19 | 55.18±4.69 | HIIT | The training time is 5min each time, 3 times a day; 3-5d postoperatively, bedside activities are the main focus, 10min each time, 3 times a day; 6-7d postoperatively, slow walking in the ward is the main focus, 15min each time, 3 times a day, and the walking distance is gradually increased from 25m, but it should be tolerated by the patient; 8-14d postoperatively, the main focus is on the training of walking, and the strength is gradually increased in combination with the patient's condition, 30min each time, 1 time a day, and it continues until 3 months postoperatively. The intensity of the training was gradually increased according to the patient's condition, with each training session lasting for 30min, once a day, until 3 months after surgery. | 24 | 3 | 45 | LVEF/6MWT/LVEDD/LVESD | Under supervision | Meets the relevant diagnostic criteria for acute myocardial infarction | AMI | Echocardiography | PCI | NR |
|  | China | 42 | 20/22 | 56.39±5.31 | AE | For the first 1-2 days post-surgery, passive movements of joints and major muscle groups are performed for 5 minutes, three times a day. From days 3-5, bedside activities are emphasized, with each session lasting 10 minutes, three times daily. On days 6-7, slow walking within the ward is prioritized, with training sessions of 15 minutes, three times a day, gradually increasing the walking distance from 25 meters based on the patient's tolerance. From days 8-14, walking training is the focus, with intensity gradually increased according to the patient’s condition, lasting 30 minutes per session, once daily, continuing for a total of 3 months post-surgery. | 24 | 7 | 30 |  |  |  |  |  |  |  |
| Yi et al. 2021 (54) | China | 39 | 21/18 | 56． 38 ±7． 06 | HIIT | During the first 7 days post-surgery, rehabilitation training methods are the same as the control group. On the 8th day, high-intensity interval training using a cardiopulmonary exercise testing system is initiated, with a rotation speed set at 50-60 rpm and power gradually increasing from 5 W/min to 15 W/min. Training should be stopped if the patient experiences shortness of breath, dizziness, systolic blood pressure exceeding 220 mmHg, or a drop of at least 10 mmHg in systolic pressure. The initial power cycling training is set at 60% of the peak power from the cardiopulmonary exercise test, followed by 1 week of adaptive training. In the second cycle, the exercise load is set at 80% of the peak power, with each training session lasting 3 minutes followed by 1 minute of rest, performing 10 sets per day, 3 times a week, for a total duration of 3 months. | 12 | 3 | 40 | LVEF/6MWT/LVEDD/LVESD | Under supervision | Meets the relevant diagnostic criteria for acute myocardial infarction | AMI | Echocardiography | PCI;  routine medications (specific drugs not specified) | NR |
|  | China | 31 | 18/13 | 55．63 ±6．37 | AE | Post-surgery, for days 1-2, patients focus on passive movements of joints and major muscle groups, with each session lasting 5 minutes, three times daily, and exercise intensity kept within 2 MET. For days 3-5, bedside activities are emphasized, gradually starting slow walking in the ward (based on tolerance), with sessions of 10 minutes, three times a day, at an intensity of about 2 MET. On days 6-7, the focus remains on slow walking in the ward (again, based on tolerance), with each session lasting 15 minutes, three times daily, gradually increasing the distance from 25 meters, at an intensity of approximately 3 MET. From days 8-14, walking training is prioritized, transitioning to cycling rehabilitation, with sessions lasting 30 minutes once daily at an intensity of around 4 MET, maintaining the same intensity for up to 3 months post-surgery. | 12 | 7-21 | 5-30 |  |  |  |  |  |  |  |
| Zeng et al. 2024 (55) | China | 45 | 22/23 | 63.02±8.54 | HIIT | Training includes jogging or brisk walking twice daily for 40 minutes each session, with a 5-10 minute interval between the two. Exercise is scheduled between 7:00 PM and 9:00 PM. Aerobic training lasts for 3 months, with maximum heart rate calculated in the same way as Group B, targeting high-intensity exercise at 71-80% of maximum heart rate. | 12 | 7 | 40 | LVEF/LVEDD/LVESD | Unclear | Meets the relevant diagnostic criteria for acute myocardial infarction | AMI | Echocardiography | Take aspirin enteric-coated tablets, atorvastatin calcium tablets, bisoprolol fumarate tablets, and isosorbide mononitrate tablets orally. | NR |
|  | China | 45 | 25/20 | 61.78±9.05 | AE | Training consists of jogging or brisk walking once daily for 40 minutes, scheduled between 7:00 PM and 9:00 PM. Aerobic training lasts for 3 months, with exercise intensity determined by maximum heart rate, calculated as 220 minus age. Moderate-intensity exercise targets a maximum heart rate of 60-70%. | 12 | 7 | 40 |  |  |  |  |  |  |  |
|  | China | 45 | 23/22 | 62.45±8.37 | Control (A) | Patients receive standard treatment, including oral enteric-coated aspirin (100 mg once daily), atorvastatin calcium (10 mg once daily), bisoprolol fumarate (5 mg once daily), and isosorbide mononitrate (20 mg twice daily). No form of exercise is performed. | 12 |  |  |  |  |  |  |  |  |  |
| Yoon Cho et al. 2018 (56) | MD | 23 | 21/2 | 53±6.84 | HIIT | Patients warm up for about 10 minutes at 40-50% of their maximum heart rate (HRmax). The HIIT consists of four 4-minute exercise intervals at 85-100% HRmax, with recovery periods at 50-60% HRmax for 3 minutes between intervals. The cool-down period is at 40-50% HRmax. Each session includes 10 minutes of stretching, 5 minutes of warm-up, 28 minutes of main exercise, and 5 minutes of cool-down, totaling 48 minutes. | 9 | 2 | 48 | 6MWT | Under supervision | Guidelines for the Diagnosis and Treatment of Acute ST-Segment Elevation Myocardial Infarction | ST- segment elevation MI | Echocardiography | PCI | NR |
|  | MD | 21 | 18/3 | 57.31±12.62 | AE | The intensity is defined as the level at which participants can sustain exercise for an extended period and should be able to converse in complete sentences. During MICT, patients work continuously at 60-70% of their maximum heart rate (HRmax). In the MICT group, the exercise program continues for the same duration, with the main exercise lasting 28 minutes at an intensity of 60-70% HRmax. | 9 | 2 | 48 |  |  |  |  |  |  |  |
| Antonello D’Andrea et.al 2022 (57) | Italy | 75 | 43/32 | 62.3 ± 8.3 | HIIT | It includes approximately 5 minutes of muscle steady-state warm-up, followed by repeated high-intensity exercises (reaching 80-90% of maximum heart rate), interspersed with moderate-intensity recovery exercises. Specifically, it consists of short-duration high-intensity exercises (≥85% peak oxygen uptake, or ≥85% heart rate reserve, or ≥90% peak heart rate) separated by low-intensity exercises. | 8 | 2 | Unclear | LVEF/LVEDD/LVESD | Under supervision | Meets the relevant diagnostic criteria for acute myocardial infarction | ST- segment elevation MI or non -ST- segment elevation MI | Echocardiography | PCI;  Drugs such as diuretics, angiotensin-converting enzyme inhibitors, angiotensin receptor blockers, and beta-blockers were used. | NR |
|  | Italy | 50 | 30/20 | 59.3 ± 15.4 | AE | Exercise at a continuous intensity (50-75% peak oxygen uptake, or 50-75% heart rate reserve, or 50-80% peak heart rate) | 8 | 2 | Unclear |  |  |  |  |  |  |  |
| Pu et al. 2017 (58) | China | 41 | 0/41 | 73.7±7.3 | MCET | Phase I:Step 1: Focus on passive movements, including slow turning, sitting up, transferring to a chair beside the bed, and using a bedside commode, with an intensity of 1 MET.Step 2: Warm-up in a seated position beside the bed and walking nearby, at an intensity of 2 METs.Step 3: Standing warm-up beside the bed and walking in the hall for 5-10 minutes, 2-3 times daily, at an intensity of 3 METs.Step 4: Standing warm-up, walking in the hall for 5-10 minutes, 3-4 times daily, plus climbing one flight of stairs or stationary cycling, with an intensity of 3-4 METs.Phase II: Outpatient Exercise Training for 6 Months:Step 1: Warm-up exercises primarily consisting of low-level aerobic activity for 5-10 minutes.Step 2: Training phase, including aerobic exercise, resistance training, and flexibility exercises, with a total duration of 30-90 minutes. Aerobic exercise forms the foundation, while resistance and flexibility exercises are supplementary:Aerobic exercise: jogging, cycling, swimming, stair climbing, and using machines for walking, cycling, or rowing, for 20-40 minutes per session, 3-5 times a week.Resistance exercise: push-ups, dumbbells or barbells, exercise machines, and resistance bands, with a maximum intensity of 50-80%.Flexibility exercises: stretch each muscle group for 6-15 seconds, gradually extending to 30 seconds; if tolerated, up to 90 seconds. Maintain normal breathing during stretches, with intensity feeling taut but not painful, repeating each stretch 3-5 times for a total of about 10 minutes, 3-5 times a week. Step 3: Relaxation exercises, which may involve continuing low-intensity aerobic activity or flexibility training, lasting 5-10 minutes; the duration may increase based on the patient's condition. | 24 | 3-5 | 30-90 | LVEF/6MWT | Under supervision | Meets the diagnostic criteria for AMI in the 8th edition of Internal Medicine | AMI | Echocardiography | PCI;  routine medications (specific drugs not specified) | NR |
|  | China | 48 | 0/48 | 70.09±6.9 | Control (B) | Both groups of patients receive health education according to the secondary prevention guidelines for coronary heart disease. This includes information on the mechanisms and risk factors for coronary heart disease and myocardial infarction, support for smoking cessation, dietary guidance for low-salt and low-fat intake, and assistance in rebuilding confidence to reintegrate into society. | 24 |  |  | LVEF/6MWT |  |  |  |  |  |  |
| Jiang et al. 2021 (59) | China | 63 | 37/26 | 64.53±5.39 | MCET | Exercise Training Plan:Postoperative 24 hours: Guide passive joint rotation activities for 10-15 minutes, 2-3 times daily.Postoperative 3-4 days: Increase bedside activity to 30 minutes.Postoperative 5-7 days: Begin ambulation with appropriate walking; initial walking distance should be manageable.Relaxation Training:a. Educational Training: Use one-on-one education, multimedia, and printed materials to introduce relaxation techniques, set goals, and encourage patient compliance and participation.b. Muscle Relaxation Training (Visualization-Relaxation Method): Create a quiet, comfortable environment for the patient to sit or lie down. Instruct them to relax all muscles, starting from the feet and moving to the face, ensuring overall relaxation. Guide the patient to breathe through the nose while silently repeating simple words. | Unclear | 7 | 30-45 | LVEF/6MWT | Under supervision | Meets the relevant diagnostic criteria for acute myocardial infarction | AMI | Echocardiography | PCI | NR |
|  | China | 63 | 35/28 | 64.09±5.47 | Control (B) | The control group receives standard intervention, including medication guidance as prescribed, continuous ECG monitoring of vital signs, and a dietary management plan. | Unclear |  |  |  |  |  |  |  |  |  |
| Dong et al. 2023 (60) | China | 55 | 34/21 | 55.67±2.34 | MCET | Aerobic Exercise:Postoperative days 5-7, patients are encouraged to walk independently in the ward for 100-150 meters, 10 minutes per session, 3 times daily. The exercise volume is gradually increased based on the patient's condition, including stair training for 15-30 minutes, 2 times daily.Resistance Training:Before resistance training, assess the patient's one-repetition maximum (1RM). Training primarily involves dumbbells, sandbags, and resistance bands, starting at 50% of 1RM. Exercises target the back, abdomen, and upper/lower limbs, including chest press, bicep curls, shoulder press, tricep extensions, quadriceps extensions, abdominal contractions, lower back extensions, and calf raises.Weeks 1-4: Each exercise performed for 1 set of 10 repetitions. Weeks 5-8: Increase to 15 repetitions for each exercise. Then, reduce the focus to 4 key exercises: bicep curls, shoulder press, tricep extensions, and chest press, performing 2 sets of 10 repetitions with 1 minute of rest between sets. If patients experience difficulty breathing or fatigue, they should stop immediately. | 8 | Unclear | 30 | LVEF/6MWT/LVEDD/LVESD | Unclear | Guidelines for the Diagnosis and Treatment of Acute ST-Segment Elevation Myocardial Infarction (2019) | AMI | Echocardiography | PCI | NR |
|  | China | 55 | 35/20 | 55.69±2.36 | Control (B) | The control group receives standard postoperative rehabilitation, including basic nursing interventions for diet, medication, and activity. | 8 |  |  |  |  |  |  |  |  |  |
| He et al. 2023 (61) | China | 55 | 30/25 | 50.34±12.08 | MCET | The observation group combines resistance training and aerobic exercise with the standard care provided to the control group. Resistance training includes the use of resistance bands, dumbbells, and sandbags, focusing on exercises such as abdominal contractions, chest presses, calf raises, quadriceps extensions, bicep curls, tricep extensions, lower back extensions, shoulder presses, and lat pull-downs. Each resistance training session lasts 10-30 minutes, with resistance increasing gradually from 50% to 80% of the maximum load.Training Schedule:Weeks 1-6: Each exercise is repeated 5 times per week.Weeks 7-12: Increase to 10 repetitions per week for each exercise.Aerobic Exercise: Starting on postoperative day 5, patients primarily use a treadmill, beginning with slow walking and gradually increasing intensity to brisk walking and light jogging. Heart rate must be monitored to maintain levels at 50-60% of maximum heart rate (calculated as 220 minus age). Each aerobic session lasts 5-10 minutes, performed twice daily with a minimum 6-hour interval between sessions. Patients should perform a 5-minute warm-up before aerobic exercise. | 24 | 14 | 30 | LVEF/6MWT/LVEDD/LVESD | Under supervision | Guidelines for the Diagnosis and Treatment of Acute Myocardial Infarction (2019) | AMI | Echocardiography | PCI;  Usual care | NR |
|  | China | 51 | 28/23 | 48.63±11.27 | Control (B) | Under the guidance of a rehabilitation therapist, patients engage in routine rehabilitation exercises. In the early postoperative phase, they start by sitting up at the bedside. Once able to get out of bed, they progress to standing by the bed and slow walking with support. This gradually transitions to walking within the ward and marching in place, with each training session lasting 3-5 minutes, performed three times daily. After discharge, patients are advised to continue daily slow walking for no more than 30 minutes per session, once a day. | 24 |  |  |  |  |  |  |  |  |  |
| Wang 2022 (62) | China | 40 | 24/16 | 58.94±5.32 | MCET | The observation group performs resistance training in addition to the control group's routine. This training includes the use of dumbbells, sandbags, and resistance bands, focusing on exercises such as abdominal contractions, lower back extensions, chest presses, calf raises, quadriceps stretches, bicep curls, lat pull-downs, shoulder presses, and tricep extensions.Training Schedule:Weeks 1-6: Each exercise is repeated 5 times.Weeks 7-12: Increase to 10 repetitions per exercise.Each training session lasts 10-30 minutes, with resistance gradually increasing from 50% to 80% of the maximum load. | 12 | 14 | 30 | LVEF/6MWT/LVEDD/LVESD | Under supervision | Guidelines for the Diagnosis and Treatment of Acute Myocardial Infarction (2019) | AMI | Echocardiography | PCI | NR |
|  | China | 40 | 23/17 | 57.19±5.47 | AE | The control group engages in routine rehabilitation exercises and aerobic training. Once patients are able to get out of bed, they practice standing by the bed and walking with support.Days 3-4 Postoperative: Indoor walking and marching in place for 3-5 minutes per session.Starting Day 5: Aerobic exercise training on a treadmill begins, gradually progressing from slow walking to increased speeds and light jogging. During exercise, patients' heart rates are monitored to maintain levels at 50-60% of their maximum heart rate (calculated as 220 minus age).Each aerobic session lasts 5-10 minutes, performed twice daily with a minimum 6-hour interval between sessions. A 5-minute warm-up is required before each workout. | 12 | 14 | 30 |  |  |  |  |  |  |  |
| Schmid et al. 2008 (63) | CH | 17 | 15/2 | 54.7±9.4 | MCET | In the ET/RT group, two ET sessions are replaced by RT. The intensity of ET is set between 70-85% of peak heart rate. The initial intensity for RT is 40% of one-repetition maximum (1-RM), increasing to 60% of 1-RM over a 4-week period.Each group performs 10 repetitions involving six different muscle groups (leg press, leg curl, back extension, abdominal contraction, oblique contraction, and lat pull-down). Rest periods between sets are 60 seconds. | 12 | 6 | Unclear | LVEF | Unclear | Meets the relevant diagnostic criteria for acute myocardial infarction | AMI | cardiac MRI | PCI | NR |
|  | CH | 21 | 17/4 | 57±9.6 | AE | The ET group completed six ET sessions. | 12 | 6 | Unclear |  |  |  |  |  |  |  |
| Chen et al. 2020 (64) | China | 42 | 24/18 | 60.32±7.11 | MCET | Postoperative Days 1-7: Demonstrate slow and fast breathing exercises beside the bed, 6 times per minute for 10 minutes each session. Assist patients with daily activities like eating, washing, turning, and using the toilet.Starting Day 8: Once the patient's condition stabilizes, continue the above exercises and select 3-4 stretching movements based on the patient's interest, each for 5 minutes. Gradually introduce strength exercises, such as weight training and squats, ensuring those with orthostatic hypotension avoid deep squats. Each exercise should consist of 10 repetitions for 1-3 sets. Assist patients with using a bedside commode and bathing in the ward.Endurance Exercises: Based on the recovery of cardiac function, gradually increase endurance training, including upper and lower limb exercises. Upper limb exercises involve alternating lifts, 15 repetitions for 1-3 sets. For lower limbs, start with 100 meters of normal walking, progress to climbing 1-2 flights of stairs, and then walk 150 meters, repeating this process after stair climbing. Both groups will undergo continuous intervention for 6 months. | 24 | 7 | 40 | LVEF | Under supervision | Guidelines for the Diagnosis and Treatment of Acute Myocardial Infarction (2019) | AMI | Echocardiography | PCI | NR |
|  | China | 42 | 26/16 | 60.39±7.14 | Control (B) | The control group receives standard nursing interventions, which include providing routine health education materials to patients and their families. Nursing staff conduct preoperative preparations, guide postoperative ambulation, and deliver exercise education before discharge according to departmental protocols. | 24 |  |  |  |  |  |  |  |  |  |
| Tang et al. 2019 (65) | China | 30 | 19/11 | 67.25±3.15 | MCET | The aerobic exercise guidance for the combined group is the same as for the aerobic group. For resistance training, patients are instructed to use equipment for chest and back muscles, core muscles, and lower body muscles.Intensity:Chest and back training: 30-40% of 1RM (where 1RM = 1.554 × 10RM weight - 5.181).Core and lower body training: 50-60% of 1RM.Training Schedule: 50 minutes per day, 3 times per week. | Unclear | 3 | 50 | LVEF | Under supervision | Guidelines for the Diagnosis and Treatment of Acute ST-Segment Elevation Myocardial Infarction | ST- segment elevation MI | Echocardiography | PCI;  Antihypertensive, lipid-regulating, antiplatelet and other drug therapies. | NR |
|  | China | 30 | 17/13 | 68.23±2.13 | AE | Patients are guided to use a treadmill (model: SportsArt T652M) and a stationary bike (model: SportsArt C55U) for aerobic exercise. The training heart rate (THR) is calculated based on the patient’s condition:THR Formula:THR = (Peak Heart Rate - Resting Heart Rate) × (60% - 80%) + Resting Heart Rate(Peak Heart Rate is the maximum heart rate achieved during exercise.)Training Schedule: Duration: 50 minutes per dayFrequency: 3 times per week.Warm-up: 10 minutes before starting aerobic exercise. | Unclear | 3 | 50 |  |  |  |  |  |  |  |
|  | China | 30 | 21/9 | 68.35±3.03 | Control (A) | Conventional medication treatment includes antihypertensives, lipid-lowering agents, and antiplatelet therapy. | Unclear |  |  |  |  |  |  |  |  |  |
| Feng 2023 (66) | China | 56 | 29/27 | 56.18±4.67 | RE | Progressive resistance training is guided by nursing staff and utilizes resistance sources such as water bottles, resistance bands, and dumbbells. The targeted muscle groups include the lower back, upper and lower limbs, and abdominal muscles. Exercises consist of free weights, abdominal contractions, back extensions, knee bends, leg lifts, and calf raises.Repetitions: 10 per setFrequency: 2-3 times perdayTraining Start: Begins one week post-surgeryResistance Progression: Start at 50% of the maximum load, gradually increasing to 75% and then 100%.Maximum Load Testing: Patients' individual maximum load capacity is assessed prior to training. | 12 | 14-21 | Unclear | LVEF/LVEDD/LVESD | Under supervision | Guidelines for the Diagnosis and Treatment of Acute ST-Segment Elevation Myocardial Infarction | AMI | Echocardiography | PCI;  routine medications (specific drugs not specified) | For angina pectoris, arrhythmia, etc. |
|  | China | 56 | 30/26 | 55.67±4.48 | AE | Early activities include getting out of bed, standing, and walking with support. On days 3-4 post-surgery, patients begin marching in place and walking exercises. Starting on day 5, aerobic training commences, beginning with slow walking and gradually transitioning to jogging. Heart rate should be maintained at 50-60% of the patient's maximum heart rate, with close monitoring throughout. | 12 | 7 | Unclear |  |  |  |  |  |  |  |
| Xu et al. 2022 (67) | China | 37 | 20/17 | 54.28±5.43 | RE | Post-surgery, progressive resistance training is combined with standard rehabilitation and mindfulness interventions.Progressive Resistance Training:Resistance Sources: Dumbbells, resistance bands, and water bottles.Target Muscle Groups: Abdominal, upper and lower limbs, and lower back.Exercises Include:Seated chest expansionShoulder pressAbdominal contractionsLower back extensions.Quadriceps stretches.Knee bends.Leg lifts.Free weights.Training Structure:Repetitions: 10 per set.Frequency: 2-3 times per day.1RM Testing: Assess individual maximum load capacity before training.Resistance Progression: Start at 50% of 1RM, gradually increasing to 75% and then 100%.Training Duration: Begins one week post-surgery and continues for 12 weeks. | 12 | 14-21 | 50 | LVEF/LVEDD/LVESD | Under supervision | Meets the relevant diagnostic criteria for acute myocardial infarction | AMI | Echocardiography | PCI;  routine medications (specific drugs not specified) | NR |
|  | China | 37 | 22/15 | 54.35±5.35 | AE | From days 4 to 6 post-surgery, the responsible nurse encourages the patient to gradually get out of bed, starting with slow sitting exercises, then progressing to walking beside the bed and indoor walking for 10 minutes per session, 2-3 times a day. From day 7, the activity range increases to outdoor walking for 5-10 minutes per session, 2-3 times a day. After discharge, patients are encouraged to maintain aerobic exercises, such as walking, brisk walking, or cycling for 30 minutes per session, 3-4 times a week. | 12 | 3-4 | 30 |  |  |  |  |  |  |  |
| Chen et al. 2024 (68) | China | 43 | 24/19 | 68.22±4.41 | MCET | Aerobic-Resistance Training:Aerobic Exercise: Focus on brisk walking. Patients start with slow walking on a treadmill, gradually increasing time and intensity until they transition to brisk walking and light jogging. Aim to maintain maximum heart rate at 50%-60% for 10-15 minutes, 2 times a day. After discharge, patients should continue jogging about 30 minutes post-meal.Resistance Training: Includes exercises like abdominal contractions, chest presses, shoulder presses, calf raises, and back extensions, using tools like resistance bands and dumbbells. Each session lasts 15-20 minutes, once daily, for at least 4 days a week. In the first 6 weeks, exercise intensity should be at 50% of 1 repetition maximum (1 RM), gradually increasing to 60% of 1 RM. The intervention lasts for 3 months post-surgery. | 12 | 2-4 | 10-25 | LVEF/6MWT/LVEDD/LVESD | Under supervision | Meets the relevant diagnostic criteria for acute myocardial infarction | AMI | Echocardiography | PCI;  routine medications (specific drugs not specified) | Muscle soreness, dizziness, etc. |
|  | China | 43 | 25/18 | 67.69±4.52 | Control (B) | Health education, preoperative preparation, postoperative activity guidance, and discharge exercise education | 12 |  |  |  |  |  |  |  |  |  |
| Ye 2023 (69) | China | 30 | 21/9 | 52.44±3.16 | MCET | Post-Discharge Rehabilitation Program:Pre-Exercise Warm-Up: At least 5 minutes of warm-up before starting any exercises.Exercise Prescription: Healthcare providers will develop an exercise plan based on the patient's condition.Cardiovascular Training:Duration: 25 minutes of aerobic exercise.Intensity:Weeks 1-6: 50%-60% of peak oxygen uptake (VO2max).Weeks 7-12: 60%-70% of VO2max.Activities: Fast walking, stationary cycling, etc.Resistance Training:Duration: 25 minutes of resistance exercise.Intensity:Weeks 1-6: 50% of one-repetition maximum (1RM).Weeks 7-12: 60% of 1RM.Target Areas: Upper and lower limb muscles, abdominal muscles, and back muscles.Exercises:Bicep curlsTricep extensionsShoulder pressAbdominal contractionsLower back extensionsQuadriceps stretches.Calf raises Lat pull-downs.Repetitions:Weeks 1-6: 10 repetitions per exercise daily.Weeks 7-12: 15 repetitions per exercise daily.Cool Down: 5 minutes of relaxation after all exercises. | 12 | 7 | 30-50 | LVEF/LVEDD/LVESD | Under supervision | Guidelines for the Diagnosis and Treatment of Acute Myocardial Infarction | AMI | Echocardiography | PCI;  routine medications (specific drugs not specified) | NR |
|  | China | 30 | 23/7 | 52.28±4.04 | Control (B) | Standing beside the bed, walking in the ward, etc., without structured exercise arrangements. | 12 |  |  |  |  |  |  |  |  |  |
| AE: aerobic exercise; RE: resistance exercise; HIIT: high-intensity interval training; MBE: mind-body exercise; MCET: multi-component exercise; 6MWT: 6-minute walk test; LVEF: left ventricular ejection fraction; LVEDD: left ventricular end-diastolic diameter; LVESD: left ventricular end-systolic diameter. MI: myocardial infarction; AMI: acute myocardial infarction; PCI: percutaneous coronary intervention; NR: not report | | | | | | | | | | | | | | | | |

Appendix 5. Summary of risk of bias


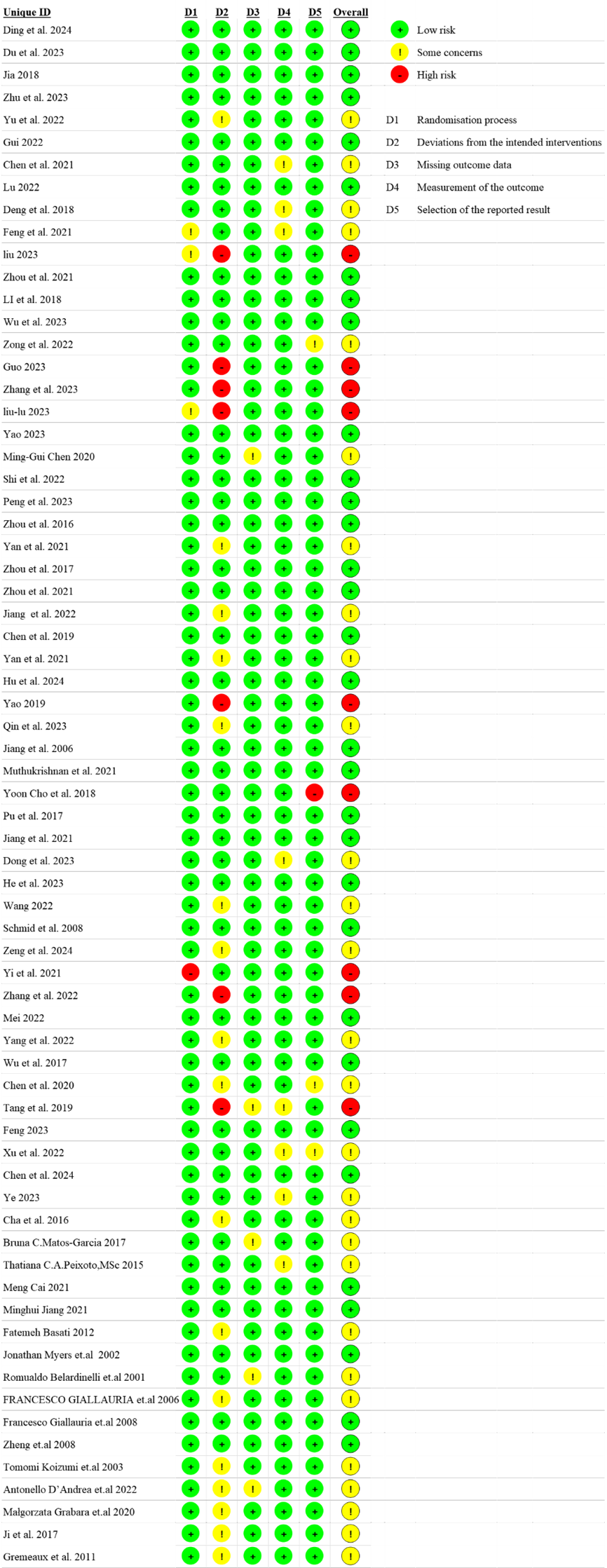


Appendix 6. Local inconsistency test

| Outcome | Side | Direct Coef. | Std. Err. | Indirect Coef. | Std. Err. | Difference Coef. | Std. Err. | P>\|z\| | tau |
| --- | --- | --- | --- | --- | --- | --- | --- | --- | --- |
| 6MWT | A-B | 55.98978 | 13.90938 | 61.71372 | 22.22177 | -5.72394 | 26.21662 | 0.827 | 37.33808 |
|  | A-C | 169.1312 | 20.74448 | 103.4795 | 26.64663 | 65.65165 | 33.77115 | 0.052 | 34.96177 |
|  | A-D | 60.75349 | 13.39361 | 93.71355 | 29.0116 | -32.9601 | 31.95456 | 0.302 | 36.60432 |
|  | A-F | 87.78966 | 19.19699 | 93.21829 | 21.44 | -5.42863 | 28.77897 | 0.85 | 37.36282 |
|  | B-C | 43.86939 | 36.1517 | 102.1642 | 21.68219 | -58.2948 | 42.15524 | 0.167 | 36.04265 |
|  | B-D | -1.240846 | 38.87462 | 11.04053 | 17.59941 | -12.2814 | 42.67289 | 0.773 | 37.34085 |
|  | B-E | 51.38468 | 20.20253 | -118.861 | 1498.123 | 170.2452 | 1498.261 | 0.91 | 36.69178 |
|  | B-F | 54.70988 | 27.38731 | 21.2352 | 19.64219 | 33.47468 | 33.70283 | 0.321 | 36.72004 |
|  | C-F | -29.12004 | 36.97467 | -64.5589 | 23.81947 | 35.43889 | 43.98287 | 0.42 | 36.95648 |
|  | D-F | -33.2799 | 36.86943 | 38.45254 | 18.7453 | -71.7324 | 41.36111 | 0.083 | 35.34725 |
| LVEF | A-B | 5.286521 | 0.733935 | 2.091202 | 1.635621 | 3.195319 | 1.792532 | 0.075 | 3.437101 |
|  | A-C | 7.455667 | 2.596452 | 9.468022 | 1.93792 | -2.01236 | 3.239928 | 0.535 | 3.535799 |
|  | A-D | 4.767774 | 0.976679 | 9.061966 | 1.991419 | -4.29419 | 2.218197 | 0.053 | 3.439085 |
|  | A-E | 5.838091 | 3.641064 | 7.921772 | 2.184428 | -2.08368 | 4.245048 | 0.624 | 3.540961 |
|  | A-F | 5.374319 | 1.398076 | 4.294645 | 1.460416 | 1.079674 | 2.021139 | 0.593 | 3.543459 |
|  | B-C | 4.168758 | 2.13969 | 3.80075 | 2.225569 | 0.368009 | 3.087263 | 0.905 | 3.547903 |
|  | B-D | 1.000021 | 3.733457 | 0.825216 | 1.135055 | 0.174805 | 3.902185 | 0.964 | 3.547273 |
|  | B-E | 2.904547 | 1.857247 | -0.88603 | 6.448968 | 3.79058 | 6.71018 | 0.572 | 3.538794 |
|  | B-F | 3.319408 | 1.690416 | -1.8084 | 1.302198 | 5.127805 | 2.133673 | **0.016** | 3.348381 |
|  | C-F | -5.860004 | 3.574444 | -3.30722 | 1.944361 | -2.55279 | 4.069052 | 0.53 | 3.5361 |
|  | D-F | -4.433364 | 2.049043 | 1.023185 | 1.421775 | -5.45655 | 2.494144 | **0.029** | 3.409172 |
| LVEDD | A-B | -2.092156 | 1.154064 | -0.13138 | 1.843387 | -1.96078 | 2.173442 | 0.367 | 3.148402 |
|  | A-C | -5.300934 | 2.326882 | -7.12006 | 1.887159 | 1.819126 | 2.995956 | 0.544 | 3.172458 |
|  | A-D | -4.380161 | 1.192238 | -8.39467 | 2.303332 | 4.01451 | 2.593556 | 0.122 | 3.05461 |
|  | A-E | -8.044089 | 3.013862 | 1.151576 | 1.894642 | -9.19567 | 3.568816 | **0.01** | 2.830616 |
|  | A-F | -4.156424 | 2.337926 | -3.9508 | 1.486274 | -0.20562 | 2.770368 | 0.941 | 3.194845 |
|  | B-C | -4.780795 | 1.920333 | -4.9525 | 2.143096 | 0.171708 | 2.87759 | 0.952 | 3.195132 |
|  | B-E | 0.7362179 | 1.553436 | -9.68397 | 5.923427 | 10.42018 | 6.129687 | 0.089 | 3.02242 |
|  | B-F | -5.423594 | 1.843908 | -0.15344 | 1.623247 | -5.27015 | 2.456694 | **0.032** | 2.936898 |
|  | C-F | 4.68996 | 3.179785 | 1.543022 | 1.930889 | 3.146938 | 3.720131 | 0.398 | 3.154671 |
|  | D-F | 3.153479 | 1.792689 | -0.8877 | 1.878538 | 4.041175 | 2.596663 | 0.12 | 3.054765 |
| LVESD | A-B | -2.405829 | 0.786217 | -0.02677 | 1.492069 | -2.37906 | 1.686729 | 0.158 | 2.485846 |
|  | A-C | -6.24769 | 1.929293 | -7.98072 | 1.497398 | 1.73303 | 2.441603 | 0.478 | 2.549366 |
|  | A-D | -2.328799 | 0.934276 | -8.74735 | 1.856637 | 6.418547 | 2.078003 | **0.002** | 2.164736 |
|  | A-E | -5.858241 | 2.555855 | -1.39734 | 1.615093 | -4.4609 | 3.024664 | 0.14 | 2.459108 |
|  | A-F | -4.908771 | 1.838262 | -3.40857 | 1.236428 | -1.50021 | 2.215386 | 0.498 | 2.542629 |
|  | B-C | -6.64241 | 1.534236 | -3.98629 | 1.680619 | -2.65612 | 2.275092 | 0.243 | 2.513092 |
|  | B-E | -0.4873366 | 1.321242 | -4.66682 | 4.790607 | 4.179478 | 4.972808 | 0.401 | 2.531491 |
|  | B-F | -3.406129 | 1.527214 | -0.81545 | 1.383647 | -2.59068 | 2.060702 | 0.209 | 2.494133 |
|  | C-F | 2.100005 | 2.563165 | 3.982113 | 1.599898 | -1.88211 | 3.021504 | 0.533 | 2.552997 |
|  | D-F | 3.396677 | 1.558519 | -3.03869 | 1.376621 | 6.43537 | 2.07937 | **0.002** | 2.164817 |

6WMT: six-minute walk test; LVEF: left ventricular ejection fraction; LVEDD: left ventricular end-systolic diameter; LVESD: left ventricular end-diastolic diameter; A: Control; B: Aerobic exercise; C: Resistance exercise; D: Mind-body exercises; E: High-intensity interval training; F: Multicomponent exercise training.

Appendix 7. Sensitivity Analysis

| Outcome | Comparison | | | | | |
| --- | --- | --- | --- | --- | --- | --- |
| 6MWT | AE |  |  |  |  |  |
|  | **-89.50 (-128.45,-50.55)** | RE |  |  |  |  |
|  | -11.30 (-44.87,22.28) | **78.21 (35.43,120.98)** | MBE |  |  |  |
|  | **-51.65 (-100.13,-3.16)** | 37.85 (-24.34,100.05) | -40.35 (-99.32,18.62) | HIIT |  |  |
|  | **-35.11 (-68.01,-2.21)** | **54.39 (13.85,94.93)** | -23.82 (-59.74,12.11) | 16.54 (-42.05,75.13) | MCET |  |
|  | **53.89 (29.07,78.71)** | **143.39 (108.40,178.39)** | **65.19 (39.01,91.37)** | **105.54 (51.07,160.01)** | **89.00 (60.26,117.74)** | Control |
| LVEF | AE |  |  |  |  |  |
|  | **-3.94 (-6.76,-1.12)** | RE |  |  |  |  |
|  | -0.08 (-2.24,2.07) | 3.30 (-0.25, 6.85) | MBE |  |  |  |
|  | -2.30 (-5.98,1.39) | 1.64 (-2.96,6.24) | -2.21 (-6.41,1.98) | HIIT |  |  |
|  | 0.60 (-1.49,2.69) | **4.53 (1.37,7.69)** | 0.68 (-1.65,3.01) | 2.89 (-1.28,7.07) | MCET |  |
|  | **4.57 (3.24,5.90)** | **8.51 (5.66,11.36)** | **4.65 (2.82,6.48)** | **6.87 (3.04,10.69)** | **3.97 (2.04,5.91)** | Control |
| LVEDD | AE |  |  |  |  |  |
|  | **4.79 (2.03,7.56)** | RE |  |  |  |  |
|  | **3.02 (0.20,5.84)** | -1.77 (-5.23,1.69) | MBE |  |  |  |
|  | 0.36 (-3.14,3.85) | **-4.43 (-8.82,-0.05)** | -2.66 (-7.01,1.68) | HIIT |  |  |
|  | 2.21 (-0.35,4.77) | -2.58 (-5.80,0.64) | -0.81 (-3.46,1.84) | 1.85 (-2.38,6.08) | MCET |  |
|  | -1.60 (-3.61,0.40) | **-6.40 (-9.26,-3.54)** | **-4.63 (-6.88,-2.37)** | -1.96 (-5.80,1.87) | **-3.82 (-6.26,-1.37)** | Control |
| LVESD | AE |  |  |  |  |  |
|  | **5.41 (3.10,7.72)** | RE |  |  |  |  |
|  | 1.68 (-0.66,4.01) | **-3.74 (-6.70,-0.77)** | MBE |  |  |  |
|  | 0.77 (-2.18,3.72) | **-4.64 (-8.34,-0.95)** | -0.91 (-4.56,2.75) | HIIT |  |  |
|  | 1.94 (-0.17,4.05) | **-3.47 (-6.19,-0.75)** | 0.27 (-2.15,2.68) | 1.17 (-2.39,4.73) | MCET |  |
|  | **-1.98 (-3.46,-0.50)** | **-7.39 (-9.77,-5.01)** | **-3.65 (-5.60,-1.71)** | -2.75 (-5.91,0.42) | **-3.92 (-5.99,-1.85)** | Control |
| AE: aerobic exercise; RE: resistance exercise; HIIT: high-intensity interval training; MBE: mind-body exercise; MCET: multi-component exercise; 6MWT: 6-minute walk test; LVEF: left ventricular ejection fraction; LVEDD: left ventricular end-diastolic diameter; LVESD: left ventricular end-systolic diameter. | | | | | | |

Appendix 8. Comparison-adjusted funnel plots


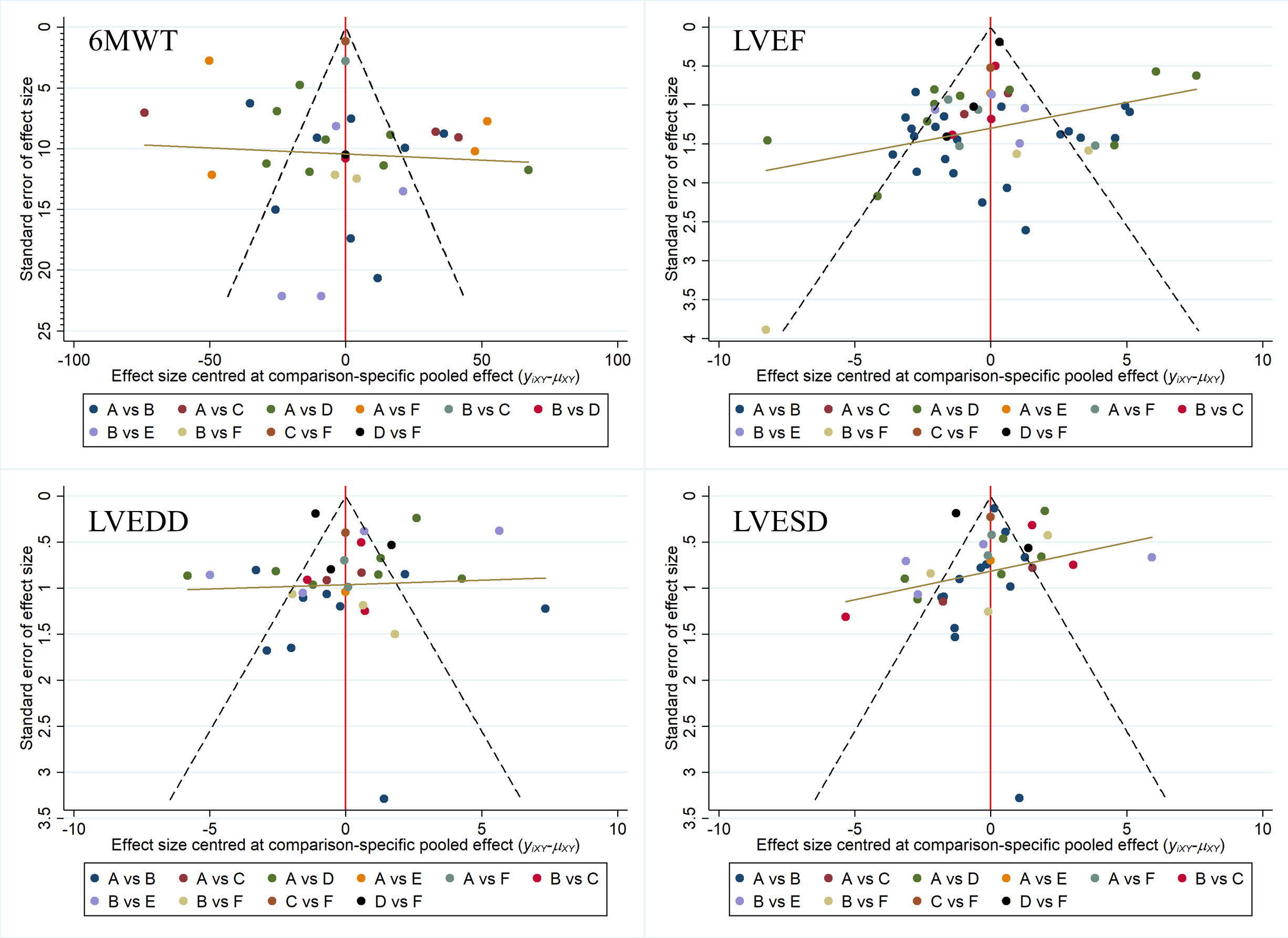


6WMT: six-minute walk test; LVEF: left ventricular ejection fraction; LVEDD: left ventricular end-systolic diameter; LVESD: left ventricular end-diastolic diameter; A: Control; B: Aerobic exercise; C: Resistance exercise; D: Mind-body exercises; E: High-intensity interval training; F: Multicomponent exercise training.

Appendix 9. Assessment of evidence quality

Appendix 9.1. Description of the assessment criteria for CINeMA

| Domains | Brief description |
| --- | --- |
| Within-study bias | The risk of bias for each included study was examined independently using the Cochrane Risk of Bias tool. The ROB for pairwise comparisons was calculated based on the ROB judgments of studies contributing to the direct and indirect meta-analysis, and the study percentage contribution matrix was calculated using the CINeMA web application. |
| Reporting bias | Publication bias was assessed using the egger test when the number of studies compared in pairs was >= 5. Publication bias was considered to exist if the p-value was <= 0.05. |
| Indirectness | Indirectness was assessed by assessing the relevance of each study to the research question. Indirectness was also assessed by detecting the effect of potential effect modifiers (Length of exercise interventions and mean age of the included population versus baseline severity) in each comparison using a reticulated meta-regression analysis. |
| Imprecision | The imprecision of each pairwise comparison is evaluated using the MD minimum significant difference value of 0. |
| Heterogeneity | Heterogeneity was assessed by comparing the 95% confidence intervals of the paired NMA estimates with the prediction intervals of the NMA estimates. |
| Incoherence | Inconsistency in the certainty of the evidence was assessed using global as well as local inconsistency tests. |

Appendix 9.2. Network plot with risk of bias


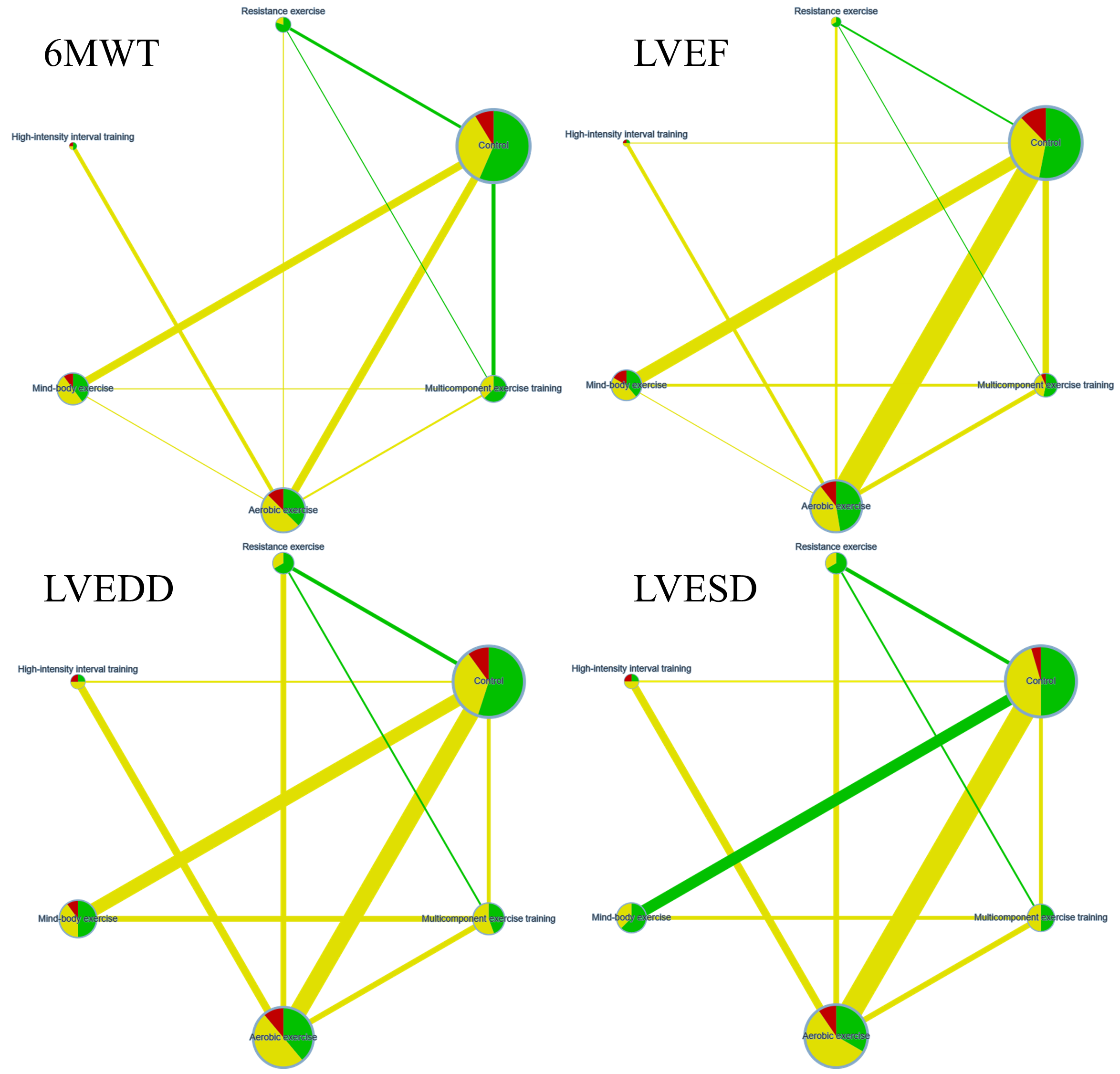


Appendix 9.3. Results of the network meta-regression

| Outcomes | Intervention |  | Covariates (Mean [95% CI]) |  |
| --- | --- | --- | --- | --- |
|  |  | Baseline severity | Mean age | Length |
| 6MWT | A | -53.19(-141.69, 35.48) | -52.725(-81.96, -23.38) | -43.24(-75.891, -11.2) |
|  | C | 90.53(-238.55, 547.51) | **91.702(46.635, 137.33)** | 96.416(-241.45, 556.52) |
|  | D | 1.699(-139.04, 114.27) | 16.116(-22.998, 55.18) | 20.681(-18.349, 59) |
|  | E | 57.957(-173.16, 283.28) | 45.785(-7.062, 98.48) | **53.253(12.446, 93.95)** |
|  | F | 24.998(-175.21, 169.99) | 35.073(-2.123, 72.29) | 34.623(-3.912, 73.46) |
| LVEF | B | 1.4195(-3.35, 6.189) | 1.41(-3.237, 6.057) | 1.4155(-3.343, 6.174) |
|  | C | **8.8567(5.609, 12.122)** | 3.1715(-5.867, 12.21) | 3.2485(-5.471, 11.968) |
|  | D | 1.8745(-3.791, 7.54) | 1.9745(-3.291, 7.24) | 1.9125(-3.591, 7.416) |
|  | E | 3.9995(-3.465, 11.464) | 3.8265(-3.663, 11.316) | 3.861(-3.647, 11.369) |
|  | F | 2.256(-2.565, 7.077) | 2.1265(-2.32, 6.573) | 2.19(-2.969, 7.349) |
| LVEDD | B | -1.154(-3.054, 0.73135) | -1.2725(-3.124, 0.579) | -1.48995(-3.423, 0.4431) |
|  | C | -2.805(-8.76, 3.14949) | -2.7605(-9.18, 3.659) | -2.93235(-9.058, 3.1933) |
|  | D | -2.32573(-7.594, 2.94254) | **-4.5748(-6.77, -2.393)** | -2.19005(-7.419, 3.0389) |
|  | E | -4.106(-8.257, 0.05309) | -0.7175(-4.009, 2.574) | -1.72205(-5.203, 1.7589) |
|  | F | -2.82497(-6.133, 0.48306) | -2.4495(-6.316, 1.417) | **-4.29041(-6.855, -1.7351)** |
| LVESD | B | -1.4046(-3.516, 0.7068) | -1.41465(-3.284, 0.4547) | -1.299875(-3.202, 0.60225) |
|  | C | **-6.8816(-9.211, -4.5808)** | -2.3775(-9.817, 5.062) | -2.235325(-9.998, 5.52735) |
|  | D | -1.92785(-5.847, 1.9913) | **-3.39163(-5.421, -1.372)** | -1.83792(-4.863, 1.18716) |
|  | E | -2.3593(-6.804, 2.0854) | -1.82545(-4.708, 1.0571) | -2.51626(-5.057, 0.02448) |
|  | F | -2.0161(-5.626, 1.5938) | -2.15255(-6.065, 1.7599) | -2.105945(-6.435, 2.22311) |
| 6MWT: 6-minute walk test; LVEF: left ventricular ejection fraction; LVEDD: left ventricular end-diastolic diameter; LVESD: left ventricular end-systolic diameter; A: Control; B: Aerobic exercise; C: Resistance exercise; D: Mind-body exercises; E: High-intensity interval training; F: Multicomponent exercise training；6MWT used B as the common control group, and LVEF, LVEDD, and LVESD used A as the common control group. | | | | |

Appendix 9.4. Details of evidence quality assessment

| Outcomes | Comparison | Within-study bias | Reporting bias | Indirectness | Imprecision | Heterogeneity | Incoherence | Confidence rating |
| --- | --- | --- | --- | --- | --- | --- | --- | --- |
| 6MWT | Aerobic exercise: Control | Some concerns | Low risk | No concerns | No concerns | Some concerns | No concerns | Low |
|  | Aerobic exercise: High-intensity interval training | Some concerns | Low risk | Some concerns | No concerns | Some concerns | No concerns | Very low |
|  | Aerobic exercise: Mind-body exercise | Some concerns | Low risk | No concerns | No concerns | No concerns | No concerns | Moderate |
|  | Aerobic exercise: Multicomponent exercise training | Some concerns | Low risk | No concerns | No concerns | Some concerns | No concerns | Low |
|  | Aerobic exercise: Resistance exercise | Some concerns | Low risk | Some concerns | No concerns | Some concerns | No concerns | Very low |
|  | Control: Mind-body exercise | Some concerns | Some concerns | No concerns | No concerns | Some concerns | No concerns | Low |
|  | Control: Multicomponent exercise training | Some concerns | Low risk | No concerns | No concerns | No concerns | No concerns | Moderate |
|  | Control: Resistance exercise | Some concerns | Low risk | No concerns | No concerns | No concerns | No concerns | Moderate |
|  | Mind-body exercise: Multicomponent exercise training | Some concerns | Low risk | No concerns | No concerns | No concerns | No concerns | Moderate |
|  | Multicomponent exercise training: Resistance exercise | No concerns | Low risk | No concerns | No concerns | Some concerns | No concerns | Low |
|  | Control: High-intensity interval training | Some concerns | Low risk | No concerns | No concerns | No concerns | No concerns | Moderate |
|  | High-intensity interval training: Mind-body exercise | Some concerns | Low risk | No concerns | No concerns | No concerns | No concerns | Moderate |
|  | High-intensity interval training: Multicomponent exercise training | Some concerns | Low risk | No concerns | No concerns | No concerns | No concerns | Moderate |
|  | High-intensity interval training: Resistance exercise | Some concerns | Low risk | No concerns | No concerns | No concerns | No concerns | Moderate |
|  | Mind-body exercise: Resistance exercise | No concerns | Low risk | No concerns | No concerns | Some concerns | No concerns | Low |
| LVEF | Aerobic exercise: Control | Some concerns | Low risk | No concerns | No concerns | Some concerns | No concerns | Low |
|  | Aerobic exercise: High-intensity interval training | Some concerns | Low risk | No concerns | No concerns | No concerns | No concerns | Moderate |
|  | Aerobic exercise: Mind-body exercise | Some concerns | Low risk | No concerns | No concerns | No concerns | No concerns | Moderate |
|  | Aerobic exercise: Multicomponent exercise training | Some concerns | Low risk | No concerns | No concerns | No concerns | Some concerns | Low |
|  | Aerobic exercise: Resistance exercise | Some concerns | Low risk | No concerns | No concerns | Some concerns | No concerns | Low |
|  | Control: High-intensity interval training | Some concerns | Low risk | No concerns | No concerns | Some concerns | No concerns | Low |
|  | Control: Mind-body exercise | Some concerns | Some concerns | No concerns | No concerns | Some concerns | No concerns | Very low |
|  | Control: Multicomponent exercise training | Some concerns | Low risk | No concerns | No concerns | Some concerns | No concerns | Low |
|  | Control: Resistance exercise | No concerns | Low risk | Some concerns | No concerns | No concerns | No concerns | Moderate |
|  | Mind-body exercise: Multicomponent exercise training | Some concerns | Low risk | No concerns | No concerns | No concerns | Some concerns | Low |
|  | Multicomponent exercise training: Resistance exercise | No concerns | Low risk | No concerns | No concerns | Some concerns | No concerns | Moderate |
|  | High-intensity interval training: Mind-body exercise | Some concerns | Low risk | No concerns | No concerns | No concerns | No concerns | Moderate |
|  | High-intensity interval training: Multicomponent exercise training | Some concerns | Low risk | No concerns | No concerns | No concerns | No concerns | Moderate |
|  | High-intensity interval training: Resistance exercise | Some concerns | Low risk | No concerns | No concerns | No concerns | No concerns | Moderate |
|  | Mind-body exercise: Resistance exercise | Some concerns | Low risk | No concerns | No concerns | No concerns | No concerns | Moderate |
| LVEDD | Aerobic exercise: Control | Some concerns | Low risk | No concerns | No concerns | No concerns | No concerns | Moderate |
|  | Aerobic exercise: High-intensity interval training | Some concerns | Low risk | No concerns | No concerns | No concerns | No concerns | Moderate |
|  | Aerobic exercise: Multicomponent exercise training | Some concerns | Low risk | No concerns | No concerns | Some concerns | Some concerns | Very low |
|  | Aerobic exercise: Resistance exercise | Some concerns | Low risk | No concerns | No concerns | Some concerns | No concerns | Low |
|  | Control: High-intensity interval training | Some concerns | Low risk | No concerns | No concerns | No concerns | Some concerns | Low |
|  | Control: Mind-body exercise | Some concerns | Low risk | Some concerns | No concerns | Some concerns | No concerns | Very low |
|  | Control: Multicomponent exercise training | Some concerns | Low risk | Some concerns | No concerns | Some concerns | No concerns | Very low |
|  | Control: Resistance exercise | No concerns | Low risk | No concerns | No concerns | Some concerns | No concerns | Moderate |
|  | Mind-body exercise: Multicomponent exercise training | Some concerns | Low risk | No concerns | No concerns | No concerns | No concerns | Moderate |
|  | Multicomponent exercise training: Resistance exercise | Some concerns | Low risk | No concerns | No concerns | No concerns | No concerns | Moderate |
|  | Aerobic exercise: Mind-body exercise | Some concerns | Low risk | No concerns | No concerns | Some concerns | No concerns | Low |
|  | High-intensity interval training: Mind-body exercise | Some concerns | Low risk | No concerns | No concerns | No concerns | No concerns | Moderate |
|  | High-intensity interval training: Multicomponent exercise training | Some concerns | Low risk | No concerns | No concerns | No concerns | No concerns | Moderate |
|  | High-intensity interval training: Resistance exercise | Some concerns | Low risk | No concerns | No concerns | Some concerns | No concerns | Low |
|  | Mind-body exercise: Resistance exercise | No concerns | Low risk | No concerns | No concerns | No concerns | No concerns | Low |
| LVESD | Aerobic exercise: Control | Some concerns | Low risk | No concerns | No concerns | Some concerns | No concerns | Low |
|  | Aerobic exercise: High-intensity interval training | Some concerns | Low risk | No concerns | No concerns | No concerns | No concerns | Moderate |
|  | Aerobic exercise: Multicomponent exercise training | Some concerns | Low risk | No concerns | No concerns | Some concerns | No concerns | Low |
|  | Aerobic exercise: Resistance exercise | Some concerns | Low risk | No concerns | No concerns | Some concerns | No concerns | Low |
|  | Control: High-intensity interval training | Some concerns | Low risk | No concerns | No concerns | Some concerns | No concerns | Low |
|  | Control: Mind-body exercise | No concerns | Low risk | Some concerns | No concerns | Some concerns | Some concerns | Very low |
|  | Control: Multicomponent exercise training | Some concerns | Low risk | No concerns | No concerns | Some concerns | No concerns | Low |
|  | Control: Resistance exercise | No concerns | Low risk | Some concerns | No concerns | No concerns | No concerns | Moderate |
|  | Mind-body exercise: Multicomponent exercise training | No concerns | Low risk | No concerns | No concerns | No concerns | Some concerns | Moderate |
|  | Multicomponent exercise training: Resistance exercise | No concerns | Low risk | No concerns | No concerns | Some concerns | No concerns | Moderate |
|  | Aerobic exercise: Mind-body exercise | Some concerns | Low risk | No concerns | No concerns | No concerns | No concerns | Moderate |
|  | High-intensity interval training: Mind-body exercise | Some concerns | Low risk | No concerns | No concerns | No concerns | No concerns | Moderate |
|  | High-intensity interval training: Multicomponent exercise training | Some concerns | Low risk | No concerns | No concerns | No concerns | No concerns | Moderate |
|  | High-intensity interval training: Resistance exercise | Some concerns | Low risk | No concerns | No concerns | Some concerns | No concerns | Low |
|  | Mind-body exercise: Resistance exercise | No concerns | Low risk | No concerns | No concerns | Some concerns | No concerns | Moderate |
| 6WMT: six-minute walk test; LVEF: left ventricular ejection fraction; LVEDD: left ventricular end-systolic diameter; LVESD: left ventricular end-diastolic diameter | | | | | | | | |

Appendix 10. Citation information for included studies

**References:**

1. D Ping, C Zhaohan, G Xiaofeng, C Hong, Z Haiyan, H Lihua. Research on the Impact of Mindfulness-Based Stress Reduction and Resistance Training on Cardiac Function and Coronary Microcirculation in Patients with Acute Myocardial Infarction After Percutaneous Coronary Intervention (PCI). *Journal of Medical Forum* (2024) 45(13):1434-8. doi: https://doi.org/10.20159/j.cnki.jmf.2024.13.019.

2. Du Liming, H Xiaojing, C Chao. Effect of Early Intensive Cardiac Rehabilitation Training Combined with Resistance Training on Cardiac Rehabilitation in Patients with Acute Myocardial Infarction Undergoing PCI. *Reflexology and Rehabilitation Medicine* (2023) 4(6):79-82. doi: https://doi.org/https://d.wanfangdata.com.cn/periodical/ChlQZXJpb2RpY2FsQ0hJTmV3UzIwMjQwNzA0EhNmc2hsZnlrZnl4MjAyMzA2MDIyGggzY29xYjE1Mg%3D%3D.

3. J Huijuan. Impact of Resistance Exercise on Cardiac Rehabilitation in Patients with Myocardial Infarction After Percutaneous Coronary Intervention (PCI). *Nursing Practice and Research* (2018) 15(01):8-11. doi: https://doi.org/https://next.cnki.net/middle/abstract?v=Dm4VI7mKrXMX_liZAqx1RvjvApjThwTuZqH2mBcIOIXoa4yXqfZt-TGfbpxIiZZ9oVtsUW0HhuxWDIEioi9YZfXpGf7P14WQ1gWV0gTUVWglI8MGbxliW17aZ6GzxNif5AIwQEsIXAOdQ3kPRrJ7HK1snA8E3TkeSQyH-BKKjt06xdxYHDwiXOx3_mHzFsUcuo4M-gAPNZc=&uniplatform=NZKPT&language=CHS&scence=null.

4. Y Lijun, W Jing, Z Lijun, W Xueli. Impact of Traditional Rehabilitation Training Combined with Elastic Band Resistance Training on Cardiopulmonary Exercise Function and Serum Inflammatory Transcription Factor Levels in Patients with Acute Myocardial Infarction After Percutaneous Coronary Intervention (PCI). *Internal Medicine of China* (2022) 17(03):344-7. doi: https://doi.org/10.16121/j.cnki.cn45-1347/r.2022.03.27.

5. W Keqin, D Jiaojiao. Elastic band resistance training improves the cardiac function in patients with acute myocardial infarction after percutaneous coronary intervention. *Chinese Journal of Geriatrics* (2017) 36(9):966-9. doi: https://doi.org/10.3760/cma.j.issn.0254-9026.2017.09.007.

6. Z Xiaoying, S Qinxia. The Effect of Seated Ba Duan Jin Combined with Cardiac Rehabilitation Program on Patients After PCI for Acute Myocardial Infarction. *YIYAOWEISHENG* (2023)(7):49-52. doi: https://doi.org/https://www.cqvip.com/doc/journal/3223641651.

7. Y Yueyan, J Dandan, Z Yujuan, Z Changlin. The Impact of Seated Ba Duan Jin on Cardiac Function Rehabilitation in Patients After PCI for Acute Anterior Myocardial Infarction. *Chinese Journal of Traditional Medical Science and Technology* (2022) 29(06):988-91. doi: https://doi.org/https://next.cnki.net/middle/abstract?v=Dm4VI7mKrXPrq_6NhYbr9cEAxAEOs8A0Lc675MqlGjUfLrH9kPMT8gcu8GlcQx2bjI7TD6UMkFXcaCTgSFuJGY9f9WSxJP9r1jSbbMBQhQyFg8LifewquCTLaMa6qM6_S5AOjk9Q3TETPVER_4fwSvI_O4A4ZuK3iyUsbvc0PjzhSG6VVy_brlPUDuoo9HrzsrZfDDTMgdw=&uniplatform=NZKPT&language=CHS&scence=null.

8. G Guangxia. The Effect of Traditional Chinese Medicine Emotional Intervention Combined with Ba Duan Jin on Rehabilitation in Patients After Myocardial Infarction Interventional Surgery. *The Medical Forum* (2022) 26(27):104-6. doi: https://doi.org/10.19435/j.1672-1721.2022.27.033.

9. C Xiaoli, M Caiyun, L Shu. The Application Effect of Yoga Exercise Program in Patients After PCI for Acute Myocardial Infarction. *Medical Journal of Chinese People's Health* (2021) 33(15):148-50. doi: https://doi.org/10.3969/j.issn.1672-0369.2021.15.059.

10. L Mei. Observation of the Application Effect of Tai Chi Combined with Ba Duan Jin in the Rehabilitation Nursing of Acute Myocardial Infarction Patients. *Reflexology and Rehabilitation Medicine* (2022) 3(10):28-31. doi: https://doi.org/https://next.cnki.net/middle/abstract?v=Dm4VI7mKrXN2PhkhvDoCIg2v8X6cGZ7_ANW58RhAEgc7BgfCjndLuu480axnhdxRWbtUnAy5AaDCWylmR6pD4r256gUQprZ9ybQRg2bkf2ePs1ltwhF-vLRFzg0S0HSeXj-XcVLI0w-7LSw0GGYYxxIwwN0tdejGRXmu8fabb3C8RumVuFqukvn41JsIKXxFqO1-sDN_kKw=&uniplatform=NZKPT&language=CHS&scence=null.

11. D Xuejun, Y Dongmei, Z Xiaoli. Effect of Taichi on heart function and psychological state in MI patients with heart insufficiency. *Journal of North Sichuan Medical College* (2018) 33(4):545-7. doi: https://doi.org/10.3969/j.issn.1005-3697.2018.04.020.

12. F Li, C Zhiling, L Fang. The Application Effect of "Five-Animal Micro Exercise" on Cardiac Rehabilitation in Patients After Percutaneous Coronary Intervention. *Chinese Journal of Interventional Cardiology* (2021) 29(09):509-14. doi: https://doi.org/ 10.3969/j.issn.1004-8812.2021.09.006.

13. L Meili. The Effect of Ba Duan Jin on Cardiac Function and Exercise Tolerance in Acute Myocardial Infarction Patients with Low LVEF After PPCI Procedure[ Ph.D Thesis]. Shengyang: Liaoning University of Traditional Chinese Medicine (2023).doi: https://doi.org/10.27213/d.cnki.glnzc.2023.000025.

14. Z Yuxuan, L Sanjun, X Wu-mei, L Yuanyuan, C Ganqiang, W Keqin. Effect of Baduanjin exercise on cardiac function and quality of life in pa-tients with heart failure after myocardial infarction. *China Modern Medicine* (2021) 28(25):73-5, 79. doi: https://doi.org/10.3969/j.issn.1674-4721.2021.25.019.

15. L Yansong, X Jingwen, J Haining, X Yinghui, W Yong. The Effect of Ba Duan Jin Combined with Medication on Cardiac Rehabilitation in Acute Myocardial Infarction Patients. *Chinese Journal of Cardiovascular Rehabilitation Medicine* (2018) 27(03):254-8. doi: https://doi.org/10.3969/j.issn.1008-0074.2018.03.04.

16. W Shujuan, M Zhenjun. Observation on the Effect of Ba Duan Jin Combined with Body-Sensing Interactive Games Intervention on Phase II Cardiac Rehabilitation in Acute Myocardial Infarction Patients. *Journal Of Frontiers Of Medicine* (2023) 13(5):90-2. doi: https://doi.org/https://d.wanfangdata.com.cn/periodical/ChlQZXJpb2RpY2FsQ0hJTmV3UzIwMjQwNzA0EhpRS0JKQkQyMDIzMjAyMzA1MTIwMDAwOTAyOBoIM2NvcWIxNTI%3D.

17. Z Shuai, C Jia, S Wei-nan, Z Peng, H Xia. Effects of Baduanjin Combined with Cardiac Exercise Rehabilitation on Cardiac Function,Exercise Endurance and Quality of Life in Patients with Acute Myocardial Infarction after Emergency PCI. *Progress in Modern Biomedicine* (2022) 22(21):4081-5. doi: https://doi.org/10.13241/j.cnki.pmb.2022.21.014.

18. G Wenjing. Clinical Observation of Ba Duan Jin Sequential Therapy on Improving Cardiac Function in Patients with Heart Failure After Acute Myocardial Infarction[Master Thesis]. Jinan: Shandong University of Traditional Chinese Medicine (2023).doi: https://doi.org/10.27282/d.cnki.gsdzu.2023.000043.

19. Z Xiaoyu, H Shiran, L Xiaoqin, Y Jian, L Chang. Discussion on the Role of Ba Duan Jin Exercise Therapy in Cardiac Rehabilitation After PCI in Acute Myocardial Infarction Patients. *China Health Standard Management* (2023) 14(21):137-40. doi: https://doi.org/10.3969/j.issn.1674-9316.2023.21.033.

20. L Lu. The Effect of Improved Ba Duan Jin on Cardiopulmonary Exercise Capacity in Elderly Patients After Cardiac Interventional Surgery for Acute Myocardial Infarction. *Health Medicine Research and Practice* (2023) 20(03):145-8. doi: https://doi.org/10.11986/j.issn.1673-873X.2023.03.035.

21. Y Longyan. The Effect of Simplified Ba Duan Jin on Cardiac Function and Quality of Life in Patients After Stent Implantation for Acute Myocardial Infarction. *YISHOUBAODIAN* (2020)(8):196. doi: https://doi.org/https://www.cqvip.com/doc/journal/987980728.

22. Chen M, Liang X, Kong L, Wang J, Wang F, Hu X, et al. Effect of Baduanjin Sequential Therapy on the Quality of Life and Cardiac Function in Patients with AMI After PCI: A Randomized Controlled Trial. *Evid-Based Compl Alt* (2020) 2020. doi: https://doi.org/10.1155/2020/8171549.

23. Grabara M, Nowak Z, Nowak A. Effects of Hatha Yoga on Cardiac Hemodynamic Parameters and Physical Capacity in Cardiac Rehabilitation Patients. *J Cardiopulm Rehabil* (2020) 40(4):263-7. doi: https://doi.org/10.1097/HCR.0000000000000503.

24. S Xiaofeng, W Huiling. Impact of Aerobic Exercise Intervention on Cardiopulmonary Function and Serum Levels of hs-CRP, IL-6, and BNP in Patients with Myocardial Infarction After PCI Surgery. *Chinese Journal of Public Health Engineering* (2022) 21(04):664-7. doi: https://doi.org/10.19937/j.issn.1671-4199.2022.04.048.

25. P Qi, Y Qin, H Guozhi, X Jun, Y Xiaoju. Value of aerobic exercise in improving cardiac function, vascular endothelial function and quality of life in patients with acute myocardial infarction with heart failure. *China Journal of Emergency Resuscitation and Disaster Medicine* (2023) 18(3):298-301. doi: https://doi.org/10.3969/j.issn.1673-6966.2023.03.006.

26. Z Daliang, Y Xiying, W Lin, Y Lifang. Research on the Improvement of Prognosis in Patients with Acute Myocardial Infarction through Aerobic Exercise. *Chinese Journal of Geriatric Heart Brain and Vessel Diseases* (2016) 18(07):702-5. doi: https://doi.org/10.3969/j.issn.1009-0126.2016.07.009.

27. Y Jian, L XiaoJing, O Ling, L Niansang, M Chao. Study on the Efficacy of Aerobic Exercise in the Rehabilitation of Patients with Acute Myocardial Infarction After PCI Surgery. *Chinese Community Doctors* (2021) 37(28):187-8. doi: https://doi.org/10.3969/j.issn.1007-614x.2021.28.090.

28. Z Daliang, Y Xiying, H Dan, Z Tianshu, W Lin. The impact of aerobic exercise on cardiac function and oxygen metabolism in patients with acute ST-segment elevation myocardial infarction. *Chinese Journal of Physical Medicine and Rehabilitation* (2017) 39(1):69-71. doi: https://doi.org/10.3760/cma.j.issn.0254-1424.2017.01.019.

29. J Yanqiong, Z Chunyan. The impact of aerobic rehabilitation exercise training intensity on myocardial perfusion, cardiopulmonary function, and quality of life in patients with myocardial infarction. *Practical Journal of Cardiac Cerebral Pneumal and Vascular Disease* (2017) 25(04):130-3. doi: https://doi.org/https://next.cnki.net/middle/abstract?v=Dm4VI7mKrXOCI9d6U19q6WFy55oLVvoNAUj_4yWel5qh8sGHVtDFCLBvmZtVOXwDqpxlFzxFDxXH4mIgYAn2bAESzDrHu1pQYx2agH3aVP5raJUJZZ28GGxnhaSNMNV1DoRo6U7BShfS2XE3eF6KNTt1t3UN9K9ENlyWRCmrSOJKhfCFKRbr0RJ9E4d3b4mfb6e7F_DYtNU=&uniplatform=NZKPT&language=CHS&scence=null.

30. Z Guoxia, D Ling, C Xiaojia. Impact of Aerobic Rehabilitation Exercise Combined with Stress Management on Cardiac Function, Psychological Status, and Quality of Life in Patients with Acute Myocardial Infarction. *Nursing Practice and Research* (2021) 18(13):1936-9. doi: https://doi.org/10.3969/j.issn.1672-9676.2021.13.011.

31. J Tonghua, T Ying, Z Yu. Analysis of the Effect of Aerobic Rehabilitation Exercise Combined with Stress Management on Improving Cardiac Function, Psychological Status, and Quality of Life in Patients with Acute Myocardial Infarction. *Science Regimen* (2022) 25(1):133-4. doi: https://doi.org/10.3969/j.issn.1672-9714.2022.01.067.

32. C Junrong, W Peng, L Baoyun, Y Fengling. The application effects of walking-based aerobic rehabilitation exercise training in the rehabilitation treatment of patients with myocardial infarction. *Clinical Research and Practice* (2019) 4(05):162-4. doi: https://doi.org/10.19347/j.cnki.2096-1413.201905068.

33. Y Jian, L Xiaojing, O Ling, L Niansang, M Chao, K Feng, et al. Clinical Study on the Rehabilitation Effect of Treadmill Exercise Combined with Conventional Western Medicine Therapy on Patients with Acute Myocardial Infarction After PCI Surgery. *Medical Innovation of China* (2021) 18(24):59-62. doi: https://doi.org/10.3969/j.issn.1674-4985.2021.24.015.

34. H Hongping, L Ping, D Fang. Application of Intensive Lipid-Lowering Therapy and Aerobic Exercise in the Post-Primary Percutaneous Coronary Intervention (PPCI) Treatment of Patients with ST-Elevation Myocardial Infarction (STEMI). *Chinese Journal of Gerontology* (2024) 44(1):4-9. doi: https://doi.org/10.3969/j.issn.1005-9202.2024.01.002.

35. Y Lulu. Study on the Effectiveness of Guiding Early Functional Exercise for Patients with Acute Myocardial Infarction Undergoing Percutaneous Coronary Intervention (PCI). *Contemporary Medical Symposium* (2019) 17(16):224-5. doi: https://doi.org/https://next.cnki.net/middle/abstract?v=Dm4VI7mKrXN5aLKxCJiB0zowqjEzENyQu7PM0skdnVMgvUefVbsWX-KbYhGO7lMcoZnoqUl_dNyJc-lfJwUVRVh-sruO9SOY2y2cwb92Yr5TE-fKp0RKHkE58O9bx0-kVPS2Z2W_2e-ahWZSJA98x9XJVn2MCnw_L0Z8m_3adY62IumnIOUe6PePIWYSnWnZKnX5_8mMmDw=&uniplatform=NZKPT&language=CHS&scence=null.

36. Q Yinghua, Z Nannan, H Yanyan. Effect of Citalopram, Exercise Therapy Combined with Interpersonal Psychotherapy on Myocardial Infarction Patients with Comorbid Mood Disorders. *Journal of International Psychiatry* (2023) 50(04):802-5. doi: https://doi.org/10.13479/j.cnki.jip.2023.04.010.

37. J Aofeng, Z Fuchun, G Wei, L Zhaoping, Z Wei, L Youwen, et al.. *Chinese Journal of Internal Medicine* (2006)(11):904-6. doi: https://doi.org/10.3760/j.issn:0578-1426.2006.11.008.

38. Muthukrishnan R, Malik GS, Gopal K, Shehata MA. Power walking based outpatient cardiac rehabilitation in patients with post-coronary angioplasty: Randomized control trial. *Physiotherapy Research International : The Journal for Researchers and Clinicians in Physical Therapy* (2021) 26(4):e1919. doi: https://doi.org/10.1002/pri.1919.

39. Gremeaux M, Hannequin A, Laurent Y, Laroche D, Casillas JM, Gremeaux V. Usefulness of the 6-minute walk test and the 200-metre fast walk test to individualize high intensity interval and continuous exercise training in coronary artery disease patients after acute coronary syndrome: a pilot controlled clinical study. *Clin Rehabil* (2011) 25(9):844-55. doi: https://doi.org/10.1177/0269215511403942.

40. Z Hongyue, X Risheng. Impact of Aerobic Exercise-Based Cardiac Rehabilitation on Exercise Tolerance and Quality of Life in Patients with Acute Myocardial Infarction. *Doctor* (2022) 7(21):139-41. doi: https://doi.org/10.19604/j.cnki.dys.2022.21.005.

41. Matos-Garcia BC, Rocco IS, Maiorano LD, Peixoto TCA, Moreira RSL, Carvalho ACC, et al. A Home-Based Walking Program Improves Respiratory Endurance in Patients With Acute Myocardial Infarction: A Randomized Controlled Trial. *Can J Cardiol* (2017) 33(6):785-91. doi: https://doi.org/10.1016/j.cjca.2016.12.004.

42. Peixoto TCA, Begot I, Bolzan DW, Machado L, Reis MS, Papa V, et al. Early Exercise-Based Rehabilitation Improves Health-Related Quality of Life and Functional Capacity After Acute Myocardial Infarction: A Randomized Controlled Trial. *Can J Cardiol* (2015) 31(3):308-13. doi: https://doi.org/10.1016/j.cjca.2014.11.014.

43. Cai M, Wang L, Ren Y. Effect of exercise training on left ventricular remodeling in patients with myocardial infarction and possible mechanisms. *World Journal of Clinial Cases* (2021) 9(22):6308-18. doi: https://doi.org/10.12998/wjcc.v9.i22.6308.

44. Jiang M, Hua M, Zhang X, Qu L, Chen L. Effect analysis of kinetic energy progressive exercise in patients with acute myocardial infarction after percutaneous coronary intervention: a randomized trial. *Ann Palliat Med* (2021) 10(7):7823-31. doi: https://doi.org/10.21037/apm-21-1478.

45. Myers J, Wagner D, Schertler T, Beer M, Luchinger R, Klein M, et al. Effects of exercise training on left ventricular volumes and function in patients with nonischemic cardiomyopathy: Application of magnetic resonance myocardial tagging. *Am Heart J* (2002) 144(4):719-25. doi: https://doi.org/10.1067/mhj.2002.124401.

46. Belardinelli R, Paolini I, Cianci G, Piva R, Georgiou D, Purcaro A. Exercise training intervention after coronary angioplasty: The ETICA trial. *J Am Coll Cardiol* (2001) 37(7):1891-900. doi: https://doi.org/10.1016/S0735-1097(01)01236-0.

47. Giallauria F, Lucci R, De Lorenzo A, D'Agostino M, Del Forno D, Vigorito C. Favourable effects of exercise training on N-terminal pro-brain natriuretic peptide plasma levels in elderly patients after acute myocardial infarction. *Age Ageing* (2006) 35(6):601-7. doi: https://doi.org/10.1093/ageing/afl098.

48. Giallauria F, Galizia G, Lucci R, D'Agostino M, Vitelli A, Maresca L, et al. Favourable effects of exercise-based Cardiac Rehabilitation after acute myocardial infarction on left atrial remodeling. *Int J Cardiol* (2009) 136(3):300-6. doi: https://doi.org/10.1016/j.ijcard.2008.05.026.

49. Zheng H, Luo M, Shen Y, Ma Y, Kang W. EFFECTS OF 6 MONTHS EXERCISE TRAINING ON VENTRICULAR REMODELLING AND AUTONOMIC TONE IN PATIENTS WITH ACUTE MYOCARDIAL INFARCTION AND PERCUTANEOUS CORONARY INTERVENTION. *J Rehabil Med* (2008) 40(9):776-9. doi: https://doi.org/10.2340/16501977-0254.

50. Koizumi T, Miyazaki A, Komiyama N, Sun K, Nakasato T, Masuda Y, et al. Improvement of left ventricular dysfunction during exercise by walking in patients with successful percutaneous coronary intervention for acute myocardial infarction. *Circ J* (2003) 67(3):233-7. doi: https://doi.org/10.1253/circj.67.233.

51. C Fubing, W Yulong, X Diwen, S Linlin, Z Binhua. The efficacy of aerobic exercise based on exercise stress test evaluation on cardiac function and psychological status in patients with myocardial infarction. *Chinese Journal of Cardiovascular Rehabilitation Medicine* (2016) 25(03):229-34. doi: https://doi.org/10.3969/j.issn.1008-0074.2016.03.01.

52. Basati F, Sadeghi M, Kargarfard M, Yazdekhasti S, Golabchi A. Effects of a cardiac rehabilitation program on systolic function and left ventricular mass in patients after myocardial infarction and revascularization. *J Res Med Sci* (2012) 17:S28-32. doi: https://www.researchgate.net/publication/286556594_Effects_of_a_cardiac_rehabilitation_program_on_systolic_function_and_left_ventricular_mass_in_patients_after_myocardial_infarction_and_revascularization.

53. M Jiwen, H Tingting. The Effect of High-Intensity Interval Training as a Vitality Enhancement Intervention Combined with Continuity Care After PCI for Acute Myocardial Infarction. *Xinxueguanbing Fangzhi Zhishi* (2022) 12(10):87-90. doi: https://doi.org/10.3969/j.issn.1672-3015(x).2022.10.026.

54. Y Hua, X Juan, P Hong, Y Ping. The Effect of High-Intensity Interval Training on Cardiac Rehabilitation After Percutaneous Coronary Intervention in Acute Myocardial Infarction Patients. *Journal of Xinxiang Medical University* (2021) 38(07):645-8. doi: https://doi.org/10.7683/xxyxyxb.2021.07.009.

55. Z Yuhua, T Di, L Yi, L Lilan, R Yugang. Effects of Different Intensity Aerobic Exercise on Cardiopulmonary Function,Vascular Endothelial Function and Quality of Life in Patients with Cardiac Dyfunction after Acute Myocardial Infarction. *Progress in Modern Biomedicine* (2024) 24(18):3549-53, 3579. doi: https://doi.org/10.13241/j.cnki.pmb.2024.18.028.

56. Ha-Yoon Choi M, Hee-Jun Han M, Ji-won Choi PHJM, Kyung-Lim Joa M. Superior Effects of High-Intensity Interval Training Compared to Conventional Therapy on Cardiovascular and Psychological Aspects in Myocardial Infarction. *Annals of Rehabilitation Medicine* (2018) 42(1):145-53. doi: https://doi.org/10.5535/arm.2018.42.1.145.

57. D'Andrea A, Carbone A, Ilardi F, Pacileo M, Savarese C, Sperlongano S, et al. Effects of High Intensity Interval Training Rehabilitation Protocol after an Acute Coronary Syndrome on Myocardial Work and Atrial Strain. *Medicina-Lithuania* (2022) 58(3). doi: https://doi.org/10.3390/medicina58030453.

58. P Jing, W Qiulin, W Peijian, H Ju, H Jixin, Z Peng. The Effect of Exercise Training on Prognosis in Female Patients After Percutaneous Coronary Intervention for Acute Myocardial Infarction. *Practical Journal of Cardiac Cerebral Pneumal and Vascular Disease* (2017) 25(12):109-13. doi: https://doi.org/10.3969/j.issn.1008-5971.2017.12.029.

59. J Anxia, D Yanjun. Study on the Effect of Exercise Therapy Combined with Rehabilitation Relaxation Training on Improving Quality of Life in Acute Myocardial Infarction Patients. *China Practical Medical* (2021) 16(26):189-91. doi: https://doi.org/10.14163/j.cnki.11-5547/r.2021.26.071.

60. D Xiaoshuang, L Ting, D Xiaoting. The Effect of Aerobic Exercise and Resistance Training Rehabilitation on Cardiac Function and Quality of Life in Patients After Interventional Treatment for Acute Myocardial Infarction. *Inner Mongolia Medical Journal* (2023) 55(1):112-4. doi: https://doi.org/10.16096/J.cnki.nmgyxzz.2023.55.01.032.

61. H Huamei, G Cheng, W Linxiu, W Xiaoyun, Z Liti, H Wenting, et al. The Effect of Resistance Training Combined with Aerobic Exercise on Rehabilitation Outcomes in Patients After PCI for Myocardial Infarction. *Journal of Minimally Invasive Medicine* (2023) 18(05):564-8. doi: https://doi.org/10.11864/j.issn.1673.2023.05.03.

62. W Jie. The Application Effect of Resistance Training Combined with Aerobic Exercise in Post-PCI Rehabilitation of Acute Myocardial Infarction Patients. *Medical Journal of Chinese People's Health* (2022) 34(02):76-8. doi: https://doi.org/10.3969/j.issn.1672-0369.2022.02.024.

63. Schmid J, Anderegg M, Romanens M, Morger C, Noveanu M, Hellige G, et al. Combined endurance/resistance training early on, after a first myocardial infarction, does not induce negative left ventricular remodelling. *European Journal of Cardiovascular Prevention & Rehabilitation* (2008) 15(3):341-6. doi: https://doi.org/10.1097/HJR.0b013e3282f5dbf5.

64. C Liping, F Jiaozhu, W Shaoqiong, W Yongjuan, L Miaochun, X Weiping, et al. The Effect of Progressive Kinetic Exercise on Improving Early Exercise Function in Patients After Percutaneous Coronary Intervention for Acute Myocardial Infarction. *Chinese Clinical Nursing* (2020) 12(01):22-5. doi: https://doi.org/10.3969/j.issn.1674-3768.2020.01.006.

65. T Yao, W Ping, Y Ping. The Effect of Aerobic and Resistance Exercise Guidance on Elderly Acute Myocardial Infarction Patients After PCI. *Contemporary Medical Symposium* (2019) 17(24):10-1. doi: https://doi.org/https://next.cnki.net/middle/abstract?v=Dm4VI7mKrXNd2HI6_xX-9G96z40jyXPRBJRvzxJ3g-lxKCfu8viRDXMbIqGtmTAjJM6MMFKtiMYqxdVL4rR1XFtGEpK6qCcX7KpON0KrvN7Frb2AAf1Sazhh5B5UnLbfgMhbZKbzCyw2VUJtRphJnksKqTILrOabi-DoaqIYQYcwRt9EpPU00DJhOxYf7r8bje2Z-KWr630=&uniplatform=NZKPT&language=CHS&scence=null.

66. F Zhiling. The Application Effect of Progressive Resistance Training Combined with Multidisciplinary Collaborative Intervention in Acute Myocardial Infarction Patients. *Xinxueguanbing Fangzhi Zhishi* (2023) 13(18):93-6. doi: https://doi.org/10.3969/j.issn.1672-3015(x).2023.18.031.

67. X Jiahui, Q Jie. The Effect of Progressive Resistance Training Combined with Mindfulness Intervention on Postoperative Rehabilitation in Acute Myocardial Infarction Patients. *Chinese Journal of Convalescent Medicine* (2022) 31(12):1287-90. doi: https://doi.org/10.13517/j.cnki.ccm.2022.12.014.

68. C Meng, S Xuan, F Yaxing, S Pei. The Application Effect of Progressive Muscle Relaxation Training Combined with Aerobic-Resistance Exercise Training in Post-Intervention Rehabilitation Nursing of Acute Myocardial Infarction Patients. *Journal Of Frontiers Of Medicine* (2024) 14(20):71-3. doi: https://doi.org/https://d.wanfangdata.com.cn/periodical/ChlQZXJpb2RpY2FsQ0hJTmV3UzIwMjQwNzA0EhpRS0JKQkQyMDI0MjAyNDA4MDIwMDAwNDQ3MBoIM2NvcWIxNTI%3D.

69. Y Wenfeng. The Effect of Combined Resistance and Aerobic Exercise on Heart Rate Variability During the Rehabilitation Period in Acute Myocardial Infarction Patients. *Xinxueguanbing Fangzhi Zhishi* (2023) 13(13):85-7. doi: https://doi.org/10.3969/j.issn.1672-3015(x).2023.13.028.
